# Real-Time Structural Illumination with Hyperspectral Images: A Tunable Projection–Capture Synchronizer for Three-Phase Demodulation on Embedded Heterogeneous Computing Platforms

**DOI:** 10.3390/s26134159

**Published:** 2026-07-01

**Authors:** Pallab Sutradhar, Alberto Martín-Pérez, Fahima Chowdhury, Yingying Gao, Rubén Rodrigues, Alejandro Martinez de Ternero, Pedro J. Lobo, Eduardo Juarez

**Affiliations:** 1 Centro de Electrónica Industrial y Sistemas Multimodales (CEIMM), Universidad Politécnica de Madrid (UPM), 28031 Madrid, Spain; a.martinp@upm.es (A.M.-P.); a.mruiz@upm.es (A.M.d.T.); pedro.lobo@upm.es (P.J.L.); 2 Aspire Institute Inc., 255 Main Street, Cambridge, MA 02142, USA

**Keywords:** real time, structured illumination, three-step phase shift, three-phased demodulation, hyperspectral imaging, SoC, FPGA, synchronization, demodulation, SFDI

## Abstract

Structured illumination (SI) involves projecting controlled spatial light patterns onto a scene or sample and processing the captured response to recover information such as the phase, surface shape, or optical contrast. Three-Step Phase Shifting (TPS), also known as three-phase demodulation (TPD) in the spatial frequency domain imaging (SFDI) field, is a common SI workflow in which three phase-shifted sinusoidal patterns are projected and captured sequentially for one demodulation. To achieve acceptable demodulation quality, all three phase-shifted patterns must be captured correctly. However, achieving this quality introduces a trade-off since each phase must be captured at the correct time, increasing acquisition time and requiring precise projector–camera synchronization. In real-time TPD-based SI, low pattern-generation throughput, synchronization uncertainty, and often bulky desktop implementations remain major limitations. Therefore, this work investigates, designs, and validates a deterministic, low-latency, and portable projection–capture synchronization system for TPD-based SI. First, a Hyperspectral (HS) Python-based SI (HSPy-SI) system, representative of common state-of-the-art (SOTA) implementations, is evaluated. It uses an HS snapshot camera and a fixed-delay desktop synchronizer. Then, the proposed HyperSI system is introduced as a real-time projection–capture synchronizer implemented as a bench-top prototype on heterogeneous embedded platforms: a Single-Board Computer (SBC) and a System-on-Chip (SoC) board. Its core contribution is a tunable parameter, W (Frame Count to Wait), which counts frame-generation interrupts before capture and reduces the delay-search space. Compared with the SOTA, HyperSI achieves over 8× higher pattern-generation throughput, increases polarized acquisition by 7× to nearly 4 FPS, reaches about 12 FPS without polarizers, and reduces waiting time by 88×.

## 1. Introduction

SI is an imaging framework that projects controlled spatial light patterns onto a scene or sample. The captured pattern response is then processed to extract the desired information, such as the surface shape, phase, or optical contrast. SI is widely used because it enables non-contact, wide-field measurement with quantitative information about the target. Common applications based on SI include fringe projection profilometry to extract 3D surface information from an object [1] and SFDI for optical-property imaging [2].

In fringe projection profilometry, sinusoidal fringe patterns are projected onto an object. The deformation of these fringes is used to recover the surface shape of the object [1]. In SFDI, sinusoidal patterns with different spatial frequencies are projected onto a turbid sample, and measured reflectance is used to estimate absorption and reduced scattering properties [2,3]. Although these fields measure different physical quantities, both use controlled illumination patterns and phase-based decoding. A common method used in these SI fields is TPS, also known as TPD in SFDI [2]. In TPD (i.e., TPS), three sinusoidal patterns projected onto a sample or scene are captured with phase shifts of 0, 2π/3, and 4π/3. These three images are then combined to remove background brightness, and the useful fringe variation is kept for the recovery of the phase or the amplitude of the spatially modulated reflectance [4,5].

As discussed earlier, SFDI is an SI technique in which the capture of three phase-shifted patterns (i.e., TPD) is one of the most important acquisition steps for optical-property recovery. Real-time projection and acquisition for TPD are often desired for use cases in fields such as medicine (e.g., in surgical settings) [6]. TPD requires multiple sequential images for a single measurement, which increases acquisition time and makes the result highly dependent on accurate projection–capture synchronization. In TPD, each camera capture must be synchronized with its corresponding projected phase pattern. If the camera is triggered too early or is out of sync with the projector phase change, the captured images may not correspond to the intended phase shift. This phase–capture mismatch reduces demodulation accuracy and can introduce artifacts (Section 2.2). Hence, TPD-based SI systems impose strict requirements on projection–capture synchronization, system latency, and deterministic timing. In addition to timing performance, SI systems also require a suitable system-level design. A real-time SI platform should support high-frame-rate acquisition with low latency while maintaining a compact, portable, and modular form factor. Such a design improves flexibility, simplifies integration, and supports deployment in space-constrained experimental or applied environments. Compatibility with standard interfaces, such as USB-based cameras and HDMI-driven projectors, is also important because it improves interoperability and reduces system complexity. Motivated by these performance and design requirements, the primary objective and contribution of this study is to investigate and develop a real-time SI system for the TPD method. The proposed work focuses on deterministic and low-latency synchronization between projection and capture while maintaining a modular and compact system architecture. Therefore, the scope of this study is explicitly limited to the system-level design, implementation, and validation of the synchronization framework.

In this study, the SOTA SFDI literature is particularly relevant for three main reasons. First, SFDI is a well-established SI technique in which conventional TPD is commonly used to achieve high-fidelity optical-property recovery. However, this study does not focus on quantitative optical-property extraction and analysis. Such analysis requires a fully validated and stable SI acquisition system as the first necessary step. Therefore, this study first establishes and validates the synchronization-based SI platform, while optical-property analysis is reserved for future work. Second, this work begins by studying an existing SOTA system: the HSPy-SI system [7]. This system was developed for SI-based imaging and was designed to accelerate TPD by using an HS snapshot camera. The system is briefly introduced later in this section and discussed in further detail in Section 3. In this study, it is used as a benchmark to evaluate the runtime performance of the proposed SI system. Third, the SFDI field has already introduced real-time alternatives to conventional TPD-based imaging, such as SSOP [8]. SSOP uses only one sinusoidal image instead of three phase-shifted images. This reduces the acquisition requirement and enables real-time imaging. However, this speed improvement comes with important trade-offs. Since SSOP estimates modulation from a single image, it can suffer from lower spatial resolution, reconstruction artifacts, edge artifacts, and reduced image quality compared with conventional TPD-based SFDI [8,9,10].

Therefore, the SOTA SFDI literature provides a suitable comparison set for this study. Beyond the primary benchmark (i.e., HSPy-SI system), reported SSOP results from the literature are also considered as a secondary reference point. This additional comparison places the proposed system within the broader context of real-time SFDI methods. Since SSOP is not implemented in this study, the comparison is limited to previously published results by other authors (Table 2). And finally, the experimental results obtained using the proposed system are used to discuss the trade-off between its acquisition speed and demodulation quality (Figure 20 in Section 5.5.14).

The development of SFDI has been accompanied by the evolution of different hardware and implementation designs, tailored to meet the different requirements of specific applications. Regardless of the application, SFDI instruments require three basic components: a light source, a spatial light modulator to generate light patterns, and a camera to capture images. Furthermore, most SFDI systems use two crossed linear polarizers between the light source and the detection system to reduce specular light. Digital micromirror devices (DMDs) are the main technology used to project spatial frequency patterns for SFDI applications; these devices are composed of two-dimensional arrays of mirrors that can be individually controlled to an on- or off-state [11]. In the on-state, the mirrors reflect the incoming light through projection optics, thereby providing light in the projection. In contrast, in the off-state, light is directed elsewhere. Most SFDI systems use DMDs [2,12,13], but they take time to switch their micromirrors on and off, so switching an 8-bit sine-wave pattern typically takes 40 ms [14]. This process can introduce variable projection latency, which in turn affects the total data acquisition time. Lowering the bit depth of the patterns can increase the DMD frame rate [15]. As an alternative, applying Fourier domain demodulation to coherent SFDI achieved 50 FPS in real time by using static film masks with printed sine waves instead of a DMD and without an HS camera [16,17,18]. For the detection systems used for SFDI systems, monochromatic cameras are commonly utilized to detect light intensity. These typically use spectral filters such as liquid crystal tunable filters (LCTFs) in conjunction with LED illumination systems to acquire images at specific wavelengths [19]. Although they are simple to use, the sequential band acquisition of LCTFs can limit temporal resolution. Thus, to reduce the acquisition time, researchers often decide to reduce the number of wavelengths to measure, taking ≈50 s [19], 13 s [20], 12 s [21,22], and even 3.6 s [23] to gather the desired wavelengths and spatial frequencies. To further increase the spectral information measured, another approach consists of using HS line scan cameras, but it can take ≈5 s to scan one phase [24]. To address scanning issues, HS snapshot sensors were used to capture spatial and spectral information in a single exposure time. Although HS snapshot sensors have not been extensively reported when applied to SFDI, Strömberg et al. [25] developed an SFDI system to evaluate HS snapshot cameras. However, their study revealed that the performance of the HS snapshot camera in getting optical properties was highly dependent on illumination and wavelength selection.

In conjunction with the interest of the research community, the development of SFDI has led to the emergence of commercial systems based on SFDI. The first company to develop a commercial research SFDI system was Modulim (Irvine, CA, USA), previously known as Modulated Imaging Incorporated, whose SFDI system for research purposes was named Reflect RS^TM^, whose first prototype was used for a preclinical study with porcine models [26]. The Reflect RS^TM^ can be programmed to measure at any spatial frequency but typically measures 10 wavelengths at five spatial frequencies, and it also accounts for height- and angle-dependent reflectance changes using well-known techniques reported in the literature [27]. The projection and acquisition of all these spatial frequencies for each wavelength in three phases takes ≈54 s for a complete data acquisition. Recently, Modulim commercialized a faster SFDI system named Clarifi^®^; however, it uses five LEDs for homogeneous illumination and only a single spatial frequency of 0.12 mm^−1^ at 850 nm, requiring approximately 10 s to acquire all data [28]. The utilization of LEDs for illumination in SFDI-based systems has become a prevalent practice among researchers in this field. Moreover, open-source online resources have recently been published on the subject of designing a low-cost SFDI system with three LEDs [29].

Despite the progress in commercial and low-cost SFDI systems, conventional TPD-based acquisition remains limited by the need to capture multiple phase-shifted images for each wavelength and spatial frequency. As the numbers of wavelengths and spatial frequencies increase, the total acquisition time also increases, which limits real-time performance and increases sensitivity to synchronization errors. To overcome this limitation, SSOP evolved as an alternative to conventional TPD-based SFDI systems. Early proof-of-concept studies showed that SSOP could achieve real-time optical-property imaging, operating at rates faster than 25 images per second, while maintaining high accuracy, with a margin of error less than 10% compared to SFDI [6]. However, this speed advantage comes with important trade-offs compared with the conventional TPD-based method. Since SSOP relies on only one captured image instead of multiple phase-shifted measurements, it initially suffered from reduced image resolution and visible reconstruction artifacts [8,9]. Although the method has been progressively refined over time to improve reconstruction quality and overall performance, current SOTA SSOP implementations still exhibit edge artifacts, provide lower spatial resolution than conventional SFDI, and do not incorporate sample profile correction [10]. These limitations reduce its suitability for clinically demanding applications, where high reconstruction fidelity and robust handling of tissue geometry are important. Although additional correction and compensation strategies can be introduced to mitigate some of these issues [6], they also increase computational overhead and add further algorithmic and system-level complexity. A temporally modulated SSOP variant has also been reported, achieving a raw video acquisition rate of 55.6 frames/s by recording a continuous sequence and separating wavelength channels through temporal FFT before applying SSOP reconstruction [30]. This improves speed relative to conventional TPD-based SFDI because it avoids sequential phase-shifted captures during acquisition. However, the gain in frame rate comes with a trade-off: the method depends on multi-frame temporal demodulation and subsequent SSOP processing, rather than delivering a direct high-quality single-frame optical-property estimate. As a result, despite faster acquisition, its applicability remains limited by reduced reconstruction fidelity, motion sensitivity, and lower image quality than conventional TPD-based SFDI.

Desktops are commonly used to generate patterns or to connect detection and projection systems [12,26,29], and microcontroller units (MCUs) have even been employed to interface the camera with the DMD [31] or to actuate mechanical elements for static-film displacement [16,17]. The absorption-reduced surface fluorescence imaging (ARSFi) method employed high-spatial-frequency SFDI using a LightCrafter evaluation module in pattern-sequence mode, reaching up to 19 FPS; however, it did not use an HS camera and remained dependent on the specific Digital Light Processing (DLP) projector model [32]. Importantly, and to the best of our knowledge, previous studies have not explored the use of an SBC-SoC development board combination to achieve real-time projection–capture synchronization. Furthermore, embedded boards offer a smaller form factor than desktop-based control hardware, which can improve system portability. They can also preserve interoperability through standardized interfaces (e.g., USB, HDMI) that simplify integration. One way to reduce the time taken to acquire spatial and spectral information is to use HS snapshot cameras and broadband illumination with lamps. This would eliminate the need for the spectral filtering done in LCTF systems and would even project the same pattern at different wavelengths using LEDs sequentially, thereby reducing acquisition time. Moreover, another fundamental aspect of correctly acquiring patterns in SFDI is ensuring that the camera and projector are synchronized correctly, which ensures the acquisition of data without artifacts for further data processing.

**Figure 1:**
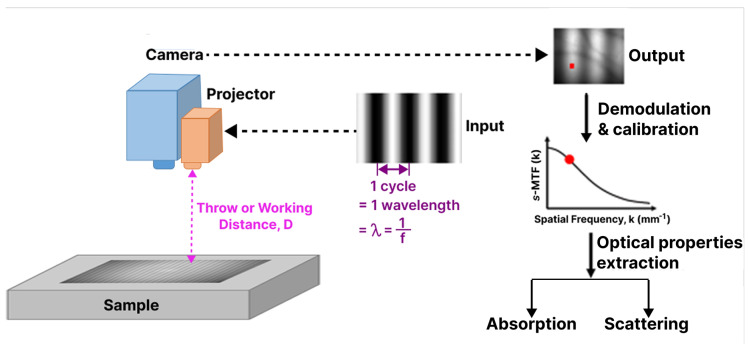
Schematic for projecting modulated pattern [3].

This research begins by studying an existing SI system known as the HSPy-SI system. It is a desktop-based projection–capture synchronization system comprising a DLP projector [33] and a Ximea HS snapshot camera [34] submodule. As the primary contribution, this study introduces HyperSI, a newly proposed synchronization system that reuses the projector and camera submodules from the existing setup and has been designed to address the limitations of the HSPy-SI system. The HSPy-SI system is considered SOTA, as it was developed in accordance with the foundational methodologies established by leading research in the SFDI field. It follows the conventional TPD method (Section 2, Figure 1) originally introduced by Cuccia et al. [12] using a DMD-based DLP projector. It can measure multiple wavelengths at a given spatial frequency, as in the early prototypes of SFDI commercial systems [26], and employs an HS snapshot camera instead of monochromatic cameras to avoid spectral filtering through external elements such as LCTFs, enabling faster acquisition of projected patterns at multiple wavelengths within one exposure time, as done by Strömberg et al. [25]. The HSPy-SI system uses the DLPLCR4500EVM projector in video mode with 24-bit RGB pattern HDMI input [33] from its own pattern generator. DLP projectors that support video mode with HDMI pattern streaming are highly versatile for projection–capture synchronization because they operate using a standardized high-quality 24-bit RGB frame-based pipeline [33,35,36,37]. Hence, they can work with maximal default settings for the video mode with minimal user intervention (e.g., only disabling gamma correction). HDMI input ensures consistent timing, format, and resolution between devices, eliminating the need for model-specific memory mapping or custom command protocols. All HDMI specifications (e.g., 1.1, 1.3, 1.4, 2.2) include an embedded VSync signal, which allows an external frame generator to lock to the frame rate of the projector. VSync is a signal that indicates the end of one frame and the start of the next in a video stream; this ensures predictable and repeatable pattern timing. Furthermore, unlike the pattern sequence mode of the projector, the HDMI video mode streams patterns continuously, supporting real-time synchronization for structured light and other fast projection–capture applications. However, different projector models offer varying default sets of supported pattern resolutions due to the specific versions of the DMD and controller they use. For example, DLPLCR4500EVM supports 912×1140 and 1280×800 [33], DLP3000DMD supports 608×684 [35], and models such as DLPLCR900EVM [36], DLPLCR90EVM and DLPLCR65EVM [37] support up to 1920×1080. These resolution differences can be accommodated by the pattern frame generator within the synchronizer, as handled by the HSPy-SI frame generator for the 912×1140 resolution. Therefore, a projection–capture synchronizer can be considered DLP-projector-agnostic for projector models that support video mode with a continuous high-quality 24-bit RGB pattern stream via the HDMI interface. Furthermore, the synchronizer can be considered snapshot-camera-agnostic when using cameras with a USB interface, since they can be integrated by managing the appropriate drivers. Vendors such as Ximea provide their own drivers to enable high-speed capture and advanced features. Alternatively, UVC (USB Video Class)-compliant snapshot cameras represent a more general USB-supported option, as they rely on a standardized driver layer on top of the core USB driver to ensure consistent basic functionality across different imaging systems. However, the Ximea HS snapshot camera is chosen as the candidate in this work because the SOTA HSPy-SI system uses the same model.

Although the HSPy-SI system provides a solid foundation and adheres to established SFDI methodologies, it is constrained by its inability to support continuous real-time acquisition. It is limited to performing only three modulated captures followed by a demodulation per cycle. Furthermore, it cannot achieve frame-accurate projection–capture synchronization due to its lack of dynamic control over the individual pattern frame generation. These factors increase system complexity and introduce real-time performance bottlenecks. In addition, the desktop form factor reduces the overall portability of the system. Hence, these limitations motivate the development of a high-performance, real-time bench-top SI synchronizer system, whose contributions are as follows: (i) providing a methodology to generate high-quality 24-bit RGB modulated patterns with high performance, such as ≈60 Hz; (ii) supporting model-agnostic frame transport to DLP projectors over HDMI in video mode; (iii) enabling camera-agnostic acquisition via standard USB snapshot cameras (i.e., USB3 HS snapshot cameras); (iv) achieving frame-accurate projection–capture synchronization using a tunable synchronization parameter; and (v) maximizing both demodulation quality (i.e., free of artifacts or noise) and runtime performance. A review of existing SOTA approaches [14,16,17,18], including the HSPy-SI system, reveals several strategies that have been employed to address real-time performance challenges in SFDI. In addition, further research was conducted to explore additional methods to improve real-time performance. To achieve high-quality pattern generation, projector and camera agnosticism, accurate synchronization, and good demodulation and runtime performance, the following methods are considered candidates for the proposed system:

1. Load the patterns into the flash memory of the DLP projector and trigger the change of patterns using a computer or an MCU [19,29,38].
2. Reduce the bit depth of the patterns on the DLP projector [14].
3. Use static film masks with printed sine waves instead of using a DLP projector [16,17,18].
4. Introduce a fixed-time waiting period between pattern changes and HS camera capture, as done in the SOTA HSPy-SI system.
5. Introduce a tunable parameter, W = Frame Count to Wait, to count the number of generated pattern frames using interrupts. After W pattern frames are counted, the HS camera is triggered to capture an image. Hence, the W tells the system that how many projected pattern frames to wait before triggering the HS camera. Then, the optimal value of W is selected, so the camera captures only after projector-buffer latency and OS-related delay have settled, ensuring that the intended pattern of a particular phase is projected correctly.

The first option introduces dependency on the chosen DLP projector model. Rather than taking a pattern input stream through a common interface (e.g., HDMI), this option relies on custom management (i.e., loading pattern images into the memory) and sequence control of the patterns. This type of pattern-generation management varies between projector models and requires maximal user intervention with a high learning curve. Hence, the synchronizer becomes more projector-model-dependent.

The second option leverages the opportunity to improve performance by reducing the bit depth of the generated patterns. Reduced bit depth introduces quantization noise that degrades the fidelity of sinusoidal patterns. As a result, the spatial frequency observed in the captured modulated images can deviate from the nominal value specified as the experimental parameter. DLP projector models use the Most Significant Bit (MSB) bit-planes to project maximum brightness and the Least Significant Bit (LSB) to project darker regions of the pattern through the micromirrors (more details will be discussed in Section 4.1). Hence, reducing the bit depth can significantly affect the quality of the generated patterns.

The third option completely eliminates the possibility of using a DLP projector. Furthermore, it must ensure an accurate mechanical positioning and trigger changes of the static films, along with the correct fixed waiting period.

In the fourth option, setting a fixed-time waiting period for changing patterns before taking an HS camera capture can achieve projection–capture synchronization. This option assumes that the DLP projector is already synchronized at the frame level with the pattern generator. If not, the synchronizer must be manually tuned by finding an optimal waiting period in seconds or milliseconds. Since the SOTA HSPy-SI system lacks frame-level synchronization, it relies on this manual tuning approach. However, it is challenging and time-consuming due to the large search space.

In addition, none of the first four options supports event-based triggering (e.g., interrupt-driven capture). As a result, they all rely on manually setting a fixed delay to align camera capture with projection, making precise synchronization difficult. Furthermore, the first and second options depend on the chosen DLP projector model; therefore, they are not suitable for a projector-model-agnostic synchronizer. The third option removes the possibility of using a DLP projector.

Finally, the fifth option implicitly uses the configurable fixed-time waiting period to compensate for frame latency caused by the DLP projector but offers more simplicity and flexibility through an interrupt-based tunable parameter, W = Frame Count to Wait.

After taking into account the limitations of options one through four, the fifth option has been explored and is thus proposed the methodology for a heterogeneous embedded board-based synchronizer, the HyperSI system. This system follows the same conventional TPD method [3,12] as the SOTA HSPy-SI system. An SBC and an SoC development board were used to develop the proposed HyperSI system. The SoC board was programmed to generate high-quality 24-bit RGB patterns for the three phases at any specific spatial frequency and send them to the DLP projector through the HDMI interface at ≈60 Hz. The DLP projector accepts and projects those patterns in video mode that require minimal user intervention (i.e., enabling video mode and disabling gamma correction). The SBC serves as the master controller; it triggers the HS snapshot camera and commands pattern changes over General-Purpose Input/Output (GPIO). Hence, the HyperSI system implements projection–capture synchronization through an SBC and SoC, whereas the SOTA HSPy-SI system uses a desktop computer for the same synchronization purpose. To improve the data acquisition rate and projection–capture synchronization, the HyperSI system introduces a tunable parameter, W = Frame Count to Wait, to compensate for the delay caused by the DLP projector. DLP projectors use precise frame-based control to project patterns, which inherently introduces frame latency due to buffering, synchronization, and bit-plane processing. As a result, any new pattern must pass through a full frame cycle (e.g., VSync-triggered buffer swap) before being fully stabilized and displayed on the DMD. The SoC can be programmed to detect the generation of an individual frame accurately through the arrival of frame-generation interrupts. Thus, the arrival period of each interrupt refers to the Time per Frame (TF), which can be measured in milliseconds. Hence, a configurable camera delay has been implemented through the counting of interrupts of each frame generation for a particular pattern phase in the SoC. Therefore, a configurable delay is applied before camera capture that can be measured in milliseconds as W×TF. After W frame interrupts have been counted, the SBC executes the capture by the HS snapshot camera. Thus, the camera delay period equals the total count of W frames; i.e., delay=W×TF. Instead of tuning a fixed waiting period in milliseconds or seconds, the proposed methodology uses a configurable delay based on the frame count of the generated pattern. Since the DLP projector displays patterns in each frame and the SBC (via the SoC) generates and changes patterns in each frame, frame-accurate synchronization can be achieved. This approach drastically reduces the search space and avoids both over- and under-optimization of the system. So finally, it is necessary to find an optimal W number of frame counts until the DLP projector stabilizes the pattern on the projection surface; i.e., until no artifact is visible in the demodulated HS image captures. Thus, W solves the projection–capture synchronization problem by implicitly compensating for the projector latency and also removes the dependence on any particular DLP projector model. Some of the aforementioned SOTA techniques perform sequential image acquisition band-by-band. The proposed methodology can also be adapted for these use cases by simply changing the camera (with USB 2 or 3) and/or projector (with HDMI). This is possible because projection–capture synchronization is not tightly coupled with any particular type of camera or projector model. The proposed system will be explored and compared with the existing SOTA HSPy-SI system. Then, this work will also evaluate the demodulation outputs and the system runtime performance of the proposed system.

The remainder of the paper is organized as follows. Section 2 explains the background of this research work with respect to the SI technique. Section 3 briefly describes the SOTA HSPy-SI system. A detailed description of the proposed HyperSI system is given in Section 4. Section 5 presents the results obtained from the experiment. Finally, Section 6 briefly discusses the results of the experiment, and Section 7 suggests ideas for future opportunities from this research work.

## 2. Background

Figure 1 illustrates how light is spatially modulated and projected onto a sample and how the resulting images are subsequently analyzed to recover the desired information. A common approach is the TPD method [3,12], in which three sinusoidal patterns are projected with phase shifts of 0°, 120°, and 240°. Capturing images at these phase steps allows the system to cancel out constant background illumination and noise while isolating the modulated component of the signal. This improves the accuracy of demodulation and ensures robust retrieval of the diffuse reflectance, which is essential for quantifying the optical properties of the tissue.

### 2.1. Modulated Pattern Generation

Cuccia et al. [12] introduced Equation (1) as the generalized form of fringe illumination (i.e., the repetition of bright–dark cycles) in the SFDI technique. This equation is used as the baseline, from which the implementation for 2D fringe pattern generation will be derived afterwards.

(1) $$S=\frac{B}{2}[1+M_{0}\cos(2\pi \times x\times f+P)]$$

Here, *S* represents the projected illumination intensity. *x* can be interpreted as time (in the time domain) or as a spatial coordinate (in the spatial domain), where it represents a wave in space rather than in time. Since SFDI deals with spatial frequencies (e.g., cycles per millimeter) rather than temporal frequencies (e.g., cycles per second), *x* is interpreted as the spatial coordinate of the pixels. *B* denotes the source intensity level, and $M_{0}$ is the modulation depth. *f* is the spatial frequency, expressed in cycles per unit length (i.e., ${\mathrm{mm}}^{-1}$), which determines the spacing of the fringes. *P* is the phase that governs the uniform shift of the generated fringes.

Since Equation (1) is formulated in the context of the spatial pixel domain, this study has derived an equivalent expression for practical implementation. In a hospital or diagnostic environment, the projector may be placed at an arbitrary distance from the projection surface to cast the fringe pattern onto the target. This distance, known as the throw distance or working distance *D*, specifies the distance between the projector lens and the projection surface. *D* can be measured manually or automatically using a Time-of-Flight (ToF) sensor. The derived expression should dynamically adjust for *D* so that the projected fringe wavelength λ (Figure 1) for the given *f* remains invariant with respect to changes in distance *D*. Here, the physical size of each cycle, i.e., λ, can be measured as $\frac{1}{f}$ mm.

**Figure 2:**
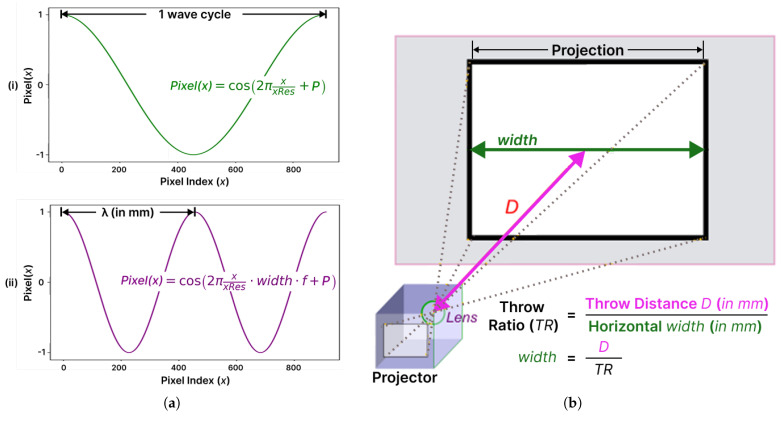
(**a**) Two wave cycles are shown for illustration: (**i**) using Equation (2), one full cosine cycle is mapped to pixel indices, representing the initial form of the equation; (**ii**) after scaling the input of the cosine function by the spatial frequency *f*, the expected number of cycles (i.e., fringes) per unit length is generated. This ensures that the physical wavelength $\lambda =\frac{1}{f}$ remains unchanged, regardless of changes in the projection distance *D*. For example, even at two different arbitrary distances (D=455 mm and 545 mm) with $f=0.122\;{\mathrm{mm}}^{-1}$, the projected wavelength computed by Equation (4) remains similar (approximately λ≈8.20 mm). (**b**) An illustration of how the projector throw ratio (TR) is calculated: the TR is defined as the ratio between the projection distance *D* and the image width *W* on the projection surface, i.e., $TR=\frac{D}{W}$ [39]. This specification is typically provided by projector manufacturers and is used to determine the projected image size at a given distance.

To begin with, a simple cosine function Pixel(x) is considered the starting point of the derivation, expressed in Equation (2). The key stages of this derivation are illustrated in Figure 2a.

(2) $$Pixel\left(x\right)=\cos\left(2\pi \times \frac{x}{xRes}+P\right)\;for,x\in \left[0,\;xRes-1\right]$$

Similarly to Equation (1), *x* in Equation (2) represents the pixel index, ranging from 0 to xRes−1, where xRes denotes the horizontal resolution of the projector. Dividing *x* by xRes normalizes the pixel coordinates to the interval [0,1], which ensures that the cosine function produces exactly one full cycle across the horizontal resolution of the entire projection (plot (i) in Figure 2a). Although a similar formulation could be written using the vertical resolution (yRes), the analysis is simplified by considering only the horizontal resolution xRes [12].

However, to use the cosine wave generated by Equation (2), it must be mapped onto a physical space (i.e., projection surface or tissue). In this mapped form, the wavelength λ of each cycle should be deterministically controlled by the desired spatial frequency *f* in ${\mathrm{mm}}^{-1}$, where $\lambda =\frac{1}{f}$ mm. Therefore, this mapping must be independent of the measured *D*. To achieve this, the term $\left(2\pi \times \frac{x}{xRes}\right)$ in Equation (2) must be scaled by the physical projection width. This width depends on the focal length and the projection angle of the projector’s lens system. However, measuring these parameters repeatedly for different camera setup heights is time-consuming and impractical in both experimental and real-world application environments. To mitigate this issue, the projector throw ratio (TR) can be used. This is a ready-to-use specification provided by manufacturers that accounts for the internal optics of the projector, as explained in Figure 2b [39]. The formula given in Figure 2b can be used to calculate the dynamic projected width with respect to the given *D*. Therefore, Equation (2) can be written as Equation (3).

(3) $$Pixel\left(x\right)=\cos\left(2\pi \times \frac{x}{xRes}\times width+P\right)\;\mathrm{where},width=\frac{D}{TR}$$

Next, the term $\left(2\pi \times \frac{x}{xRes}\times width\right)$ in Equation (3) needs to be scaled by the spatial frequency *f* to generate more wave cycles (i.e., fringes) across the projection width, since *f* defines how many cycles fit into each mm of physical space. Hence, Equation (3) can be written as Equation (4) and is illustrated in plot (ii) in Figure 2a.

(4) $$Pixel\left(x\right)=\cos\left(2\pi \times \frac{x}{xRes}\times width\times f+P\right)$$

The cosine expression in Equation (4) produces values within [−1,1]. However, pixel intensities must have non-negative values within a displayable range. To apply this equation for pattern generation in practice, the output must be shifted and scaled to the range [0,B], where *B* represents the maximum brightness (i.e., 255 for 8-bit pixel values). This is done by first shifting the cosine output to [0,1] (by adding 1 and dividing by 2) and finally scaling the cosine expression in Equation (4) by *B*. This adjustment ensures that the generated fringe patterns have valid pixel intensity values, as expressed in Equation (5).

(5) $$Pixel\left(x\right)=\frac{B}{2}\left(\cos\left(2\pi \times \frac{x}{xRes}\times \frac{D}{TR}\times f+P\right)+1\right)\;\mathrm{where},\frac{D}{TR}=width$$

Hence, the final expression in Equation (5) ensures that the wavelength of the generated fringe patterns remains invariant to the throw distance (i.e., measured by ToF sensor). Therefore, both the SOTA HSPy-SI and the proposed system use Equation (5) for pattern generation.

### 2.2. Demodulation

In the field of SFDI, the demodulation procedure is critical for transforming raw imaging data into diffuse reflectance, which serves as the basis for subsequent analysis. The theory behind SFDI has been extensively reported in the literature [3,12,14]. Briefly, the first step involves the acquisition of at least two spatial frequencies, of which one should be acquired at a low spatial frequency and the other at a high spatial frequency, $f_{x}$. Typically, these are acquired at $f_{x}=0.00\;{\mathrm{mm}}^{-1}$, which means that no pattern is projected and only homogeneous illumination is used, and at $0.10\leq f_{x}\leq 0.20\;{\mathrm{mm}}^{-1}$ [12,22,23,26]. With both spatial frequencies, the spatially resolved modulation transfer function (s-MTF) or, equivalently, the diffuse reflectance for every spatial frequency can be reconstructed to estimate $\mu_{a}$ and $\mu_{s}^{\prime }$ [12]. Acquiring a spatial frequency typically involves projecting a 2D sine-wave pattern three times, displaced from each other by 2π/3. Importantly, only light intensity variations in space must be confined to the *x*- or *y*-axes, with the *x*-axis being the prevailing choice in the existing literature. After acquiring the three projected 2D sine waves, $I_{1}\left(x\right)$, $I_{2}\left(x\right)$, and $I_{3}\left(x\right)$, with the camera, Equation (6) is used to demodulate the signals and extract the envelope of the reflected intensity captured by the camera $M_{AC}\left(f_{x},x\right)$ at a specific $f_{x}$ for each pixel in the image. Both the SOTA HSPy-SI and the proposed system use Equation (6) to perform demodulation.

(6) $$M_{AC}\left(f_{x},x\right)=\frac{\sqrt{2}}{3}\sqrt{{\left[I_{1}\left(f_{x},x\right)-I_{2}\left(f_{x},x\right)\right]}^{2}+{\left[I_{2}\left(f_{x},x\right)-I_{3}\left(f_{x},x\right)\right]}^{2}+{\left[I_{3}\left(f_{x},x\right)-I_{1}\left(f_{x},x\right)\right]}^{2}}$$

The demodulation process is complex when building an SI system, especially with the three-pattern projection approach [29]. Various factors may cause an erroneous demodulated image, characterized by the presence of artifacts within the resulting $M_{AC}\left(f_{x},x\right)$ image. The three-pattern projection approach is sensitive to motion artifacts, minor movements of the sample, and the imaging system. These can result in residual patterned structures in the demodulated images due to errors during acquisition of the three phase-shifted patterns. Such errors are attributed to variations in ambient or stray light, fluctuations in light intensity across phases, and the nonlinear response of the projector [14], with the latter resulting in the projection of non-pure sinusoidal patterns. This nonlinearity further distorts the demodulation process and reduces the quality of the resulting images [14]. Any of the issues described above can introduce higher-order harmonics into the acquired data, further distorting the demodulation process and thus reducing the quality of the demodulated images. More specifically, these harmonics generated a demodulated image with a residual pattern at a frequency that is three times that of the illumination pattern [14].

### 2.3. Preprocessing of the Demodulated HS Image

Demodulated HS snapshot image captures need to be preprocessed by white calibration [40] and spectral correction [41], as required by the camera manufacturer. The calibration step ensures data reproducibility and corrects for variations in lighting conditions. After calibration, it is essential to perform the spectral correction. The raw energy measured by each of the spectral filters must be corrected before any further processing. In practice, real filters cannot produce a single pure harmonic, which results in secondary harmonics. Moreover, adjacent pixels also exhibit crosstalk from energy leakage at their boundaries. Therefore, a spectral correction is needed as the next step after calibration [42].

Equation (7) performs the HS image calibration [43]. Here, $Img^{\prime }$, Img, $W_{ref}$ and $D_{ref}$ represent the calibrated HS image, the source HS image, and the white and dark reference images, respectively. And Equation (8) computes the spectrally corrected [43] pixel vector $P_{i}^{\prime }$, where $P_{i}$ is the spectral vector of pixel *i* (with each component of the vector representing the intensity of one spectral band) of the HS image, and *C* represents the correction matrix provided by the camera manufacturer.

(7) $$Img^{\prime }=\left(Img-D_{ref}\right)/\left(W_{ref}-D_{ref}\right)$$

(8) $$P_{i}^{\prime }=P_{i}*C$$

### 2.4. Phase-Plot Smoother

Phase plots were used in this research to verify and confirm the correct demodulation by showing continuous spatial variations across three phases. After applying a smoothing algorithm, a phase plot can show clearer symmetry and periodicity that match the projected patterns. Visual cues from a smooth phase plot facilitate quick detection of misalignment in phase shifts that may cause artifacts in demodulation. To illustrate a smooth phase plot from raw pixel-intensity vectors extracted from image rows, an unweighted sliding average smoothing algorithm [44] can be applied, reducing random fluctuations and highlighting the underlying phase shift in captured modulated HS images. Equation (9) is used to smooth the phase plots, where $P_{j}$ represents the *j*-th pixel of an image row and is replaced by the mean of its adjacent neighbors *w*. Here, *w* is a user-defined smoothing width, chosen as an odd positive integer to ensure a symmetric window. $C_{i}$ denotes the original value of the *i*-th pixel.

(9) $$P_{j}\;=\;\frac{1}{w}\sum_{i=j-\frac{w-1}{2}}^{\;j+\frac{w-1}{2}}C_{i}$$

## 3. SOTA HSPy-SI System

The desktop-based SOTA HSPy-SI system implements the three-phase pattern projection using a DMD-based DLP projector. It can capture multiple wavelengths using the HS snapshot camera at any chosen spatial frequency. This contributes to faster HS image acquisition. This system considers the incorporation of polarizers into both the DLP projector and the HS snapshot camera. These polarizers eliminate specular reflections, ensuring that only the diffuse reflectance of the scene is captured.

### 3.1. HSPy-SI Components

This section briefly reviews the components used and their specifications. The desktop (i.e., used as synchronizer) has an approximate form factor (i.e., dimensions) of 400 mm × 165 mm × 370 mm (length × width × height). The details can be found in Appendix A.1.

### 3.2. HSPy-SI System Design

This section expands on the description of the HSPy-SI system design. The details can be found in Appendix A.2.

### 3.3. Operational Workflow

This section presents the operational workflow. The details can be found in Appendix A.3.

### 3.4. Capture Methodology

This section describes the capture methodology used for SOTA HSPy-SI image acquisitions. The details can be found in Appendix A.4.

## 4. Materials and Methods

**Figure 3:**
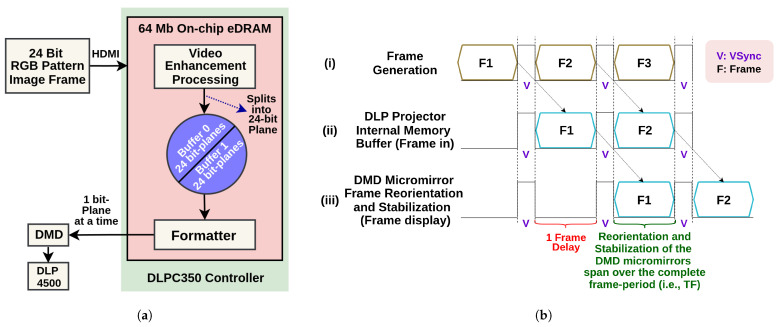
The internal frame processing and timing behavior of the employed DLPLCR4500EVM DLP projector are illustrated as follows: (**a**) Internal frame processing managed by the DLPC350 controller in video mode. (**b**) The one-frame display delay in the DLP projector caused by the internal dual-buffer mechanism. Row (**i**) shows frame generation with VSync pulses; row (**ii**) depicts the DLP internal memory buffer receiving frames at each VSync rising edge, as indicated by diagonal arrows from the frame-generation timeline; and row (**iii**) shows DMD micromirror reorientation and stabilization occurring with a one-frame delay (marked in red); thus, the first frame F1 requires the entire frame period (1 TF) to reorient and stabilize.

In an ideal scenario, when a pattern frame for a specific phase is changed and projected, the HS camera connected to the computer should immediately capture that updated pattern on the projection surface. However, in practice, this is not the case. The DLP projector introduces latency when rendering a pattern frame for two reasons: (i) the dual-buffer architecture and (ii) the time required for micromirror reorientation and stabilization (Figure 3a,b). As a result, immediately after a phase change, the HS camera is unable to capture the correct pattern projected on the projection surface. To address this issue and achieve reliable projection–capture synchronization, this study proposes the HyperSI system as its primary contribution.

Section 4.1 briefly discusses how the projector introduces latency. Section 4.2 explains how the novel W = Frame Count to Wait parameter has been integrated with the projection–capture synchronization solution. Section 4.3 lists the components used for the proposed system. Section 4.4 illustrates the system design of the proposed methodology. Section 4.5 outlines the methodology used to develop and evaluate the proposed system. Section 4.6 and Section 4.7 describe the development flow in the SoC and SBC environments, respectively. Finally, Section 4.8 explains the implementation of projection–capture synchronization.

### 4.1. Latency Caused by the DLP Projector to Refresh a Pattern Frame

**Figure A4:**
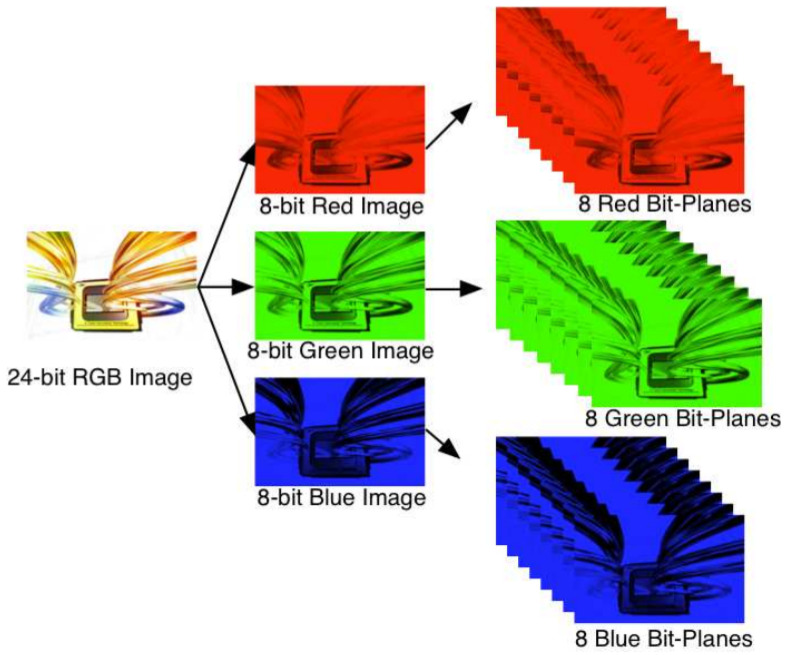
Decomposition of a 24-bit RGB image into three color channels: red, green, and blue. Each of those is further divided into 8 individual bit-planes [45].

Figure 3a shows an abstract overview of how the DLPC350 proprietary controller manages the pattern stream from an input 24-bit RGB parallel interface to the DMD within the DLP^®^ LightCrafter^™^ E4500 MKII^™^ projector used in this experiment. Each complete pattern frame projected by the projector, whether retrieved from internal flash memory or generated by an external pattern source, is expressed in the projector datasheet as an n-bit frame. For example, a frame containing three 8-bit channels (RGB) is classified as a 24-bit frame [45]. The DLP projector has two modes: video and pattern sequence. The video mode has been selected because it works with minimal user intervention for the default settings. A 24-bit RGB pattern is received through HDMI and passed to the Video Enhancement Processing (i.e., Video Processing) block. This block performs various processing tasks (such as Degamma, Primary Color Correction, Chroma Interpolation, Scalar, Overlap Color Processing) on the input digital image. Internally, the DLPC350 stores two 24-bit frames in its memory, resulting in a 48-bit frame display buffer that works like a ping-pong buffer. It allows the DLPC350 to send one 24-bit frame to the DMD micromirror array, while the second buffer is filled with the new frame streamed through the 24-bit parallel RGB (i.e., HDMI) or Flat Panel Display (FPD)-Link interface. After the display of the previous frame is completed, the VSync pulse coming with each frame through HDMI triggers a buffer swap, and the newly filled frame from the other 24-bit frame buffer is delivered to the DMD, one bit-plane at a time (further explanation given in Appendix B.1, Figure A4). Subsequently, the DMD sends each of the received bit-planes to the micromirrors. The DLPC350 controller intertwines and interleaves bit-planes, per-color time slots, and color frames to improve image quality. Finally, the controller uses a frame period to initialize and stabilize a pattern, as illustrated in row (iii) of Figure 3b. This figure also simplifies the 48-bit frame buffer shown in Figure 3a by representing it as a timeline DLP Projector Internal Memory Buffer (Frame in) in row (ii). Row (i) shows frame generation with VSync pulses, with the first frame F1, then F2, and so on. Row (ii) shows the DLP internal buffer that receives a new frame F1 at each VSync rising edge (diagonal arrows indicate transfer). Row (iii) shows DMD micromirror reorientation and stabilization with a one-frame delay (marked in red); the first F1 frame then requires the full frame period (1 TF) to settle. Thus, a frame period is spent in the buffer; therefore, a one-frame display latency always exists between the received pattern input and the output image rendered by the DMD micromirror array. Another frame period is spent reorienting and stabilizing the micromirrors to project a stable frame.

The way in which the controller utilizes an entire frame period to initialize and stabilize a frame after it is released from the buffer is further detailed in Appendix B.1.

### 4.2. Projection–Capture Synchronization with W Parameter

The SOTA HSPy-SI system uses a fixed waiting time to compensate for DLP projector latency when capturing a modulated HS image at the correct pattern phase. The DLP LightCrafter 4500 projector is driven by the DLPC350 controller, which internally implements a dual-frame ping-pong buffer architecture when operating in video-streaming mode [33,45]. In this architecture, one frame is projected on the Digital Micromirror Device (DMD) while the next incoming frame from the HDMI interface is simultaneously buffered. Consequently, the optically projected frame corresponds to the previously transmitted HDMI frame, introducing an inherent pipeline latency of approximately one frame period (i.e., TF) between frame transmission and optical projection (Section 4.1). This deterministic one-frame delay requires that the camera capture be delayed appropriately so that the acquisition occurs when the intended SI pattern is fully stabilized on the projection surface.

However, finding the exact frame-based delay before camera capture is difficult in the SOTA HSPy-SI implementation because the system relies on millisecond-scale waiting times that cannot account for factors such as precise frame-generation timing, dispatch latency during phase switching, and runtime overhead introduced by the operating system (OS). As a result, selecting an appropriate delay requires extensive trial and error across a large search space. To address this limitation, the proposed system introduces a frame-based synchronization parameter W = Frame Count to Wait. Instead of specifying a delay in milliseconds, W defines the number of generated frames for which the system waits before triggering the camera capture. Since the frame-generation process occurs at a known Frame Refresh Rate (FRR), this approach effectively provides a deterministic compensation for the projector pipeline latency. Thus, the delay applied before camera capture becomes proportional to the number of frame interrupts detected by the frame generator.

Accurate control of individual pattern-frame generation at a typical 60 Hz FRR is difficult to achieve on consumer-grade hardware such as SBCs or desktop PCs. On these platforms, the video output is abstracted and managed by multiple layers of the OS, including user applications, shell environment, system libraries, runtime components, system call interfaces, kernel services, hardware abstraction layers, and finally the hardware itself. This layered architecture prevents deterministic control over individual frame generation. Although some MCU evaluation boards support video output at resolutions such as 1024×768 [46], 480p [47], 1280×800 [48], and 480×320 to 800×480 [49], most of these boards lack an integrated HDMI interface and do not support the resolution requirements of the selected DLP projector. For example, the RP2040-PiZero includes a DVI interface capable of driving HDMI displays, but its maximum resolution is limited to 640×480 [50].

**Figure 4:**
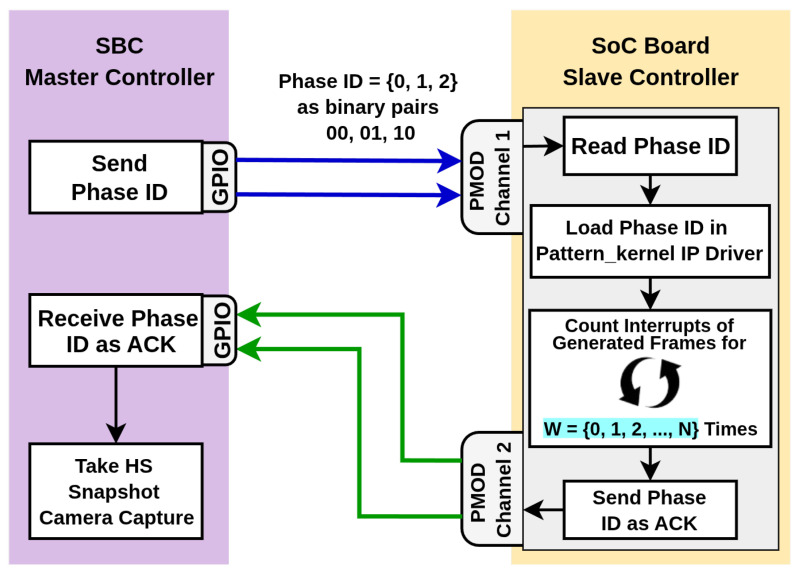
The core design principle of the projection–capture synchronizer for achieving frame-level synchronization is illustrated. An SBC (master) and an SoC (slave) communicate via GPIO or equivalent ports (e.g., PMOD) in half-duplex mode. The SBC triggers the HS snapshot camera and commands pattern changes on the SoC, while the SoC detects individual frame generation through interrupts. A tunable parameter W (Frame Count to Wait) compensates for the DLP projector delay; after W frame interrupts, the SoC acknowledges the SBC to execute the camera capture.

**Figure 5:**
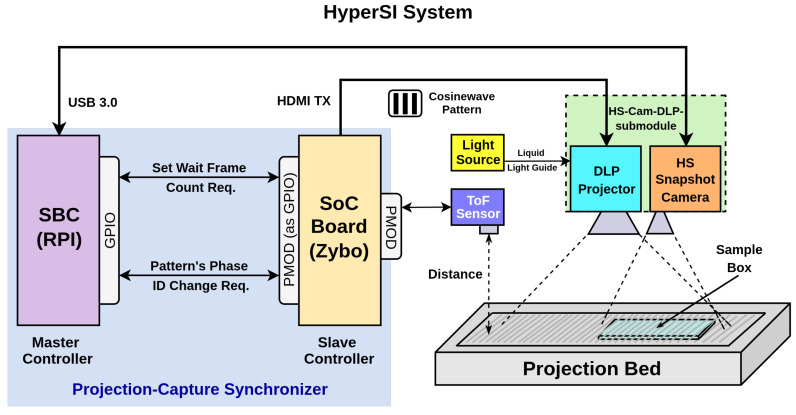
Schematic of HyperSI system design.

Based on these design considerations, a heterogeneous embedded architecture was selected, as shown in the proposed solution concept and the final system design in Figure 4 and Figure 5, respectively. An SoC development board with an HDMI transmitter (TX) peripheral is used for high-performance pattern generation and frame dispatch to the DLP projector. Typically, such SoC platforms contain programmable logic (PL) and an embedded processor. The PL is responsible for generating the SI patterns, while the processor controls the pattern-generation process. The SoC board must also provide PMOD interfaces to connect auxiliary sensors, such as a ToF sensor used to measure the distance between the projector and the projection target. The measured distance can then be provided to the pattern generator implemented in the PL.

To operate the HS snapshot camera, a USB3 interface is required. However, most SoC development boards lack a USB3 interface to reduce manufacturing costs, manage limited board space, and satisfy the low-bandwidth requirements of typical peripherals. Therefore, an external computing device with a USB3 interface is required to control the camera. An SBC with USB 3.0 connectivity is suitable for this purpose because of its compact form factor and suitability for space-constrained environments. Since the SBC controls camera acquisition, it also orchestrates pattern changes on the SoC board. Communication between the SBC and the SoC is therefore established through GPIO pins following a half-duplex communication protocol. In this architecture, the SBC acts as the master controller, responsible for camera triggering and pattern phase switching, while the SoC operates as the slave controller, responsible for deterministic frame generation.

Within this proposed design architecture, as illustrated in Figure 4, the SoC uses the parameter W to control the generation and counting of individual pattern frames through frame-synchronous interrupts. The parameter W can be explained using an example scenario. The DLPC350 controller requires at least two consecutive input frames of the same pattern (e.g., phase 0) before the micromirrors stabilize and the corresponding pattern is fully projected onto the target surface (Section 4.1). If the SoC generates pattern frames with a period of approximately 17.00 ms (corresponding to a 60 Hz FRR), setting W=2 results in a capture delay of approximately 2×17.00=34.00 ms before the SBC triggers the HS image acquisition. In this way, the frame-based delay mechanism directly compensates for the one-frame projection latency introduced by the DLPC350 display pipeline while ensuring that the camera captures the stabilized pattern phase. Compared to millisecond-based delays, the W-based synchronization approach significantly reduces the search space for delay tuning (i.e., compensation) and provides deterministic control over the number of stable projected frames observed by the camera. This parameter is critical for validating frame-level synchronization while performing the modulated captures and their effect on subsequent demodulation quality.

Section 5.5.3 describes the frame-level synchronization validation procedure used to verify the correct temporal alignment between pattern projection and camera capture. In that section, the Change Pattern Time (CPT) is formally defined using Equation (10), where the capture delay is expressed as the sum of the static delay (SD, i.e., delay introduced for GPIO communication overhead), and sequential frame interrupts are counted according to the configurable parameter W. Since each interrupt interval $I_{i}$ approximately equals the frame period TF, the parameter W directly determines the number of frame periods that must elapse before initiating capture. Furthermore, Equation (11) derives the timing of the first interrupt $I_{1st}$ from the measured CPT values, enabling verification of the temporal relationship between frame generation and capture triggering.

The parameter W therefore plays a central role in both defining and validating the synchronization delay. By controlling the number of frame interrupts counted before capture, W effectively compensates for the deterministic projection latency introduced by the DLP projector pipeline described in Section 4.1. Validating the CPT and the corresponding $I_{1st}$ values ensures that the capture is triggered only after the intended pattern frame has propagated through the projector pipeline and stabilized on the projection surface. This verification step is essential for guaranteeing correct phase alignment between the projected pattern and the captured image, which directly affects the quality of the modulated captures and the accuracy of the subsequent demodulation process.

### 4.3. HyperSI Components

The HyperSI system utilizes a Raspberry Pi (RPI) 4 Model B as the SBC and a Digilent Zybo Z7-20 development board as the SoC platform. The used RPI 4 Model B has an approximate form factor (i.e., dimension) of 90 mm × 53 mm × 22 mm (length × width × height), while the used Zybo Z7-20 SoC evaluation board has an approximate form factor of 122 mm × 88 mm × 15 mm. The HS-Cam-DLP-submodule, illustrated in Figure 5, houses the HS snapshot camera and the DLP projector. This submodule, originally used in the SOTA HSPy-SI system, is reused in the proposed design to maintain HW consistency. It integrates the HS snapshot camera, the DLP projector, polarizers, a light source, and a liquid light guide (LLG). More details are provided in Appendix B.2.

### 4.4. HyperSI System Design

Figure 5 summarizes the concept of the complete system. Two heterogeneous embedded boards, an RPI as the SBC and a Zybo z7-20 development board as the SoC, are used to achieve projection–capture synchronization. The SoC board generates cosine-wave patterns for three different phases in real time with 1 Pixel-per-Clock (PPC) performance and controls the ToF sensor. The distance between the DLP projector and the projection surface can be measured using that ToF sensor and passed to the parameterized pattern-generator IP. The ToF sensor should be positioned as far as possible from the camera’s Field of View (FOV) so that the infrared light emitted by the sensor does not affect the reflected light coming out of the target sample box toward the camera. A light source feeds the infrared light to the DLP projector through an LLG so that the patterns are visible on the projection bed. An SBC-based system controls the HS camera to capture the HS image. It also controls the change (i.e., shifting) of the projection of the cosine-wave pattern among the 0°, 120°, and 240° phases in the SoC board. For simplicity, these respective phases are denoted by phase IDs = {0,1,and2} consecutively, i.e., P0, P1, and P2. This change is performed through the GPIO interface, which follows a half-duplex communication mode. After HS image capture of the projection surface for a particular phase of the pattern, the SBC tells the SoC to switch to the next pattern phase through GPIO signaling. Since the SBC controls the pattern projection on the SoC board, it acts as the master controller, while the SoC board serves as the slave controller.

### 4.5. Development Methodology

**Figure 6:**
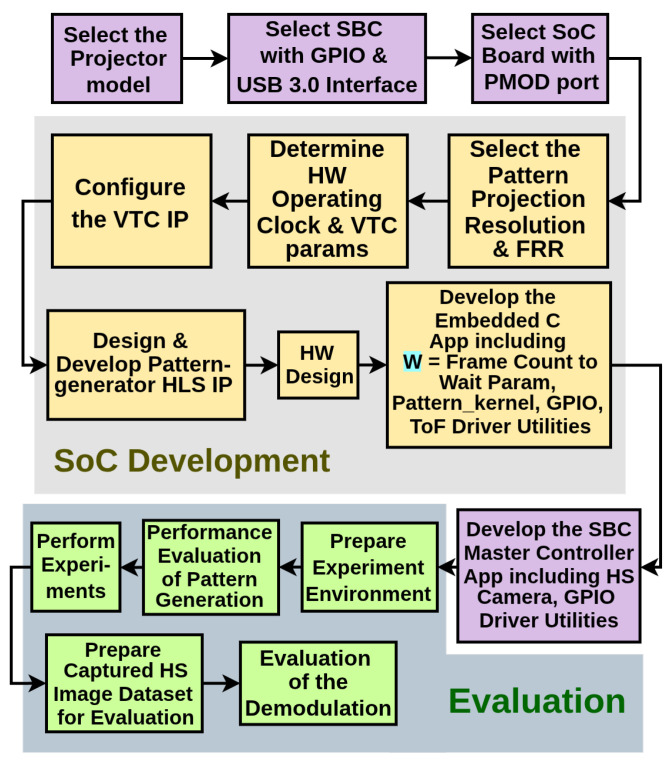
Schematic of the development methodology of the proposed system.

Figure 6 illustrates the complete development methodology, including the evaluation framework for the proposed system.

**Select the projector model:** A DLP projector evaluation model should be selected that supports video mode with continuous high-quality 24-bit RGB pattern streaming through the HDMI interface. Since the SOTA HSPy-SI system uses the DLPLCR4500EVM, which meets all the required specifications, the same projector has been selected for the proposed design. This choice also ensures consistency in capture experiments, allowing a fair comparison between the two systems.

**Select an SBC with GPIO and a USB 3.0 Interface:** A Raspberry Pi (RPI) 4 Model B is used as the SBC-based system. The board provides two USB 3.0 ports, making it a suitable candidate for interfacing with and controlling an HS camera. A standard 40-pin GPIO header allows GPIO communication with other systems.

**Select an SoC Board with a PMOD port:** The Digilent Zybo Z7-20 development board model uses the AMD Xilinx Zynq-7020 SoC. The board has 6 PMOD ports that can be used for the GPIO interface with external systems (i.e., with an SBC).

**Select the Pattern Projection Resolution and FRR:** In the proposed design, the SoC is responsible for generating the pattern frames. Hence, its core hardware (HW) components (i.e., pattern-generator and Video Timing Controller (VTC) parameters) that are directly responsible for frame generation must be configured to match the required resolution and FRR (i.e., a non-standard resolution 912×1140 @ 60 Hz FRR) of the chosen DLP projector (Section 4.6.1).

**Determine the HW operating clock frequency and VTC parameters:** Since the HW has been designed from scratch, the core operating clock frequency (in MHz) must be determined to configure the entire HW system. Additionally, the VTC parameters need to be calculated to ensure the proper display of patterns across devices such as the DLP projector or a standard monitor. This process is particularly challenging because the frames must be generated for a non-standard resolution. To compute the core HW clock and VTC parameters, two key parameters must be determined: the pixel clock and the PPC requirement of the HDMI IP. The first step involves determining the pixel clock based on the selected resolution and FRR. To ensure consistent and reliable frame timing across Video Electronics Standards Association (VESA)-compliant devices, the pixel clock was calculated using the VESA standard. In addition, this standard also provided the required core VTC parameters. Next, the PPC count requirement was obtained (i.e., 1 PPC) from the HDMI IP provided by the board vendor. Finally, using the pixel clock and the PPC value, the clock frequency of the core HW was determined (Section 4.6.2).

**Configure the VTC IP:** The VTC IP provided by the embedded development software (SW) vendor (i.e., AMD Xilinx; v2022.2) needs to be configured in generation mode because all the parameters were pre-defined for a given resolution and FRR. The VTC IP provides the video sink (i.e., AXI4-Stream to Video Out IP (AXI4S-VOut)) with all pre-calculated required timing parameters: active frame size, blanking intervals, sync pulse widths/polarities, and frame size. These parameters let the sink control the flow of pixels with TREADY, add the right HSync/VSync signals, and produce a stable video output for HDMI (Section 4.6.3).

**Design and develop the pattern-generator HLS IP:** In high-performance frame generation, the video pipeline must remain tightly in sync with the timing of the AXI4S-VOut IP sink. Designing the pattern-generator IP for 1 PPC ensures deterministic behavior, where each handshake cycle corresponds to exactly one pixel. Furthermore, at least 1 PPC is necessary to guarantee that the generator can always keep up with the pixel clock expected by the video sink (i.e., AXI4S-VOut IP), maintain proper AXI4-Stream handshaking, and avoid underflows or sync errors. A methodology for developing a parameterized pattern generator (i.e., Pattern_kernel) capable of 1 PPC is presented using a dataflow model of computation (MoC) implemented through the HLS development approach (Section 4.6.4). In particular, this approach supports pattern generation at any given resolution.

**Develop the HW design:** The HW design for the SoC has been developed by integrating all essential IP cores required for synchronized pattern generation and video output. This includes the Zynq-7000 PS IP, a custom-developed pattern-generator IP, the VTC IP, the AXI4S-VOut IP, and interrupt-enabled AXI GPIO IPs. Together, these components enable the frame-accurate control and synchronization necessary for real-time SI operation (Section 4.6.5).

**Develop the embedded SoC controller application:** The embedded SoC controller application is responsible for generating parameterized patterns and implementing interrupt-based delays based on the W parameter. It also manages the ToF sensor for distance measurement, which is used as input to the pattern generator. However, these operations are not autonomously controlled. Instead, the SoC application functions as a standalone slave, receiving commands via GPIO communication from the SBC master controller. This setup enables centralized control through the master application (Section 4.6.6).

**Develop the SBC master controller application:** The primary responsibility of the SBC application is to perform HS image captures and coordinate phase changes on the SoC board through GPIO in a synchronized manner (Section 4.7). Furthermore, Section 4.8 outlined the projection–capture synchronization mechanism, which involves coordination between the embedded application running on the SoC board and the master controller application on the SBC.

**Prepare the experimental environment:** SI-based techniques such as SFDI require a controlled experimental environment, including stable ambient lighting and consistent projector conditions, to ensure reliable HS image acquisition (Section 5.1). To maintain consistency in the evaluation, identical pattern-generation parameters were applied to both the SOTA HSPy-SI system and the HyperSI system (Section 5.1.1). The HS snapshot camera was then configured (Section 5.1.2). As the SOTA HSPy-SI synchronizer was developed solely for use with polarizers, and the proposed HyperSI supports both polarizer and no-polarizer settings, optimal camera exposure times must be determined for each setting to prevent overexposure.

**Evaluate pattern-generation performance:** The pattern-generation performance of the proposed HyperSI system was evaluated with respect to the accuracy at the pixel level and the speed of frame generation (Section 5.2). For a fair comparison, identical pattern-generation parameters were applied to the SOTA HSPy-SI system and the HyperSI system. The generated patterns from both systems were compared to verify whether the HyperSI implementation preserves the integrity of the pixel value (Section 5.2.1). In addition, the FRR was measured to assess the execution speed achieved by the implementation of the HW-accelerated real-time pattern-generator kernel relative to the SW-based SOTA system (Section 5.2.2). These analyses determine whether the HyperSI system fulfills the objective of producing high-quality 24-bit RGB modulated patterns at ≈60 Hz performance.

**Perform experiments:** Two settings were defined based on the exposure times determined for the camera polarizer and no-polarizer configurations. However, the proposed system includes an additional configuration parameter, W. Taking all of these factors into account, the complete scheme of experimental capture configurations was established (Section 5.3). Before the final target samples were captured, three acquisition steps were required. First, modulated white reference images were captured and demodulated to support preprocessing of both reference and final target sample images. Second, the target sample was prepared for acquisition (Section 5.3.1). Lastly, the reference and final modulated target samples were captured (Section 5.3.2), with the reference captures serving as a baseline to evaluate the final results.

**Prepare captured HS image dataset for evaluation:** Since each HS camera capture contains 24 spectral bands, analyzing and evaluating all of them would be overwhelming. Therefore, a smaller subset of bands was first selected (i.e., 7 bands) based on spectrometer energy reflectance experiments (Section 5.4.1). This subset was then preprocessed, demosaiced, and used to generate raw and smoothed phase plots. From these, four bands with better signal quality and contrast were finally selected for further evaluation (Section 5.4.2).

**Evaluate demodulation:** Evaluation criteria must be defined to assess demodulated images produced by the HSPy-SI and HyperSI systems (Section 5.5.1). The evaluation of the SOTA HSPy-SI captures was straightforward, as it has no configurable delay and supports only the polarizer setting (Section 5.5.2). In contrast to the SOTA system, the captures performed by the proposed system are directly influenced by the interrupt-based tunable parameter W, which controls the number of frame-based interrupts used to delay camera capture. To achieve frame-level synchronization, the interrupt arrival period must match the frame period, i.e., I≈TF. Therefore, a detailed experimental analysis was performed to measure the actual interrupt arrival periods and examine their timing behavior with respect to the W parameter (Section 5.5.3). The results confirmed that *I* consistently matched with TF and also validated the design choice (Section 4.8) of the first interrupt originating from the frame of the previous phase. These findings are critical for ensuring the accuracy of W, which determines the synchronization delay used to compensate for projector latency. The frame-sequence timeline has been introduced to establish a phase-synchronization and projection analysis framework that ensures that the demodulation evaluation accounts for every key event occurring during the experimental capture. By integrating the timing of SoC interrupts, the DLP dual-buffer behavior, the DMD micromirror stabilization process, and camera frame perception, the timeline provides a unified representation of how all components interact across phases for any W configuration (Section 5.5.4). Real-time-modulated captures, along with their corresponding demodulated outputs for a range of positive W values (0–3), were evaluated using the same frame-sequence timeline concept (Section 5.5.5, Section 5.5.6, Section 5.5.7, Section 5.5.8, Section 5.5.9, Section 5.5.10, Section 5.5.11 and Section 5.5.12). The equations for the minimum required W have been derived to guarantee proper phase alignment and stable projection timing between the SoC and the DLP projector (Section 5.5.13). Finally, the runtime performance for both systems was analyzed (Section 5.5.14).

### 4.6. Develop the HW/SW System in the SoC Environment

The core responsibilities of the HW/SW system are to generate and stream the pattern through HDMI for configurable parameters (i.e., distance, phase, spatial_frequency, throw_ratio, brightness), to control the ToF sensor for distance measurement, and to feed the distance value to the IP for pattern generation. The RPI SBC controls the pattern generation for particular phase IDs, i.e., 0, 1, and 2, representing 0°, 120°, and 240° phases in the SoC-based system, through the GPIO half-duplex communication mode. This is why the Zybo SoC board acts as a slave controller with respect to the RPI SBC.

The use of proprietary AMD Xilinx SW for developing the HW/SW pattern projection application is further described in the Additional Materials in the introductory part of Appendix B.4. The development of the embedded SoC board application follows the SoC Development block of the development methodology given in Figure 6. These steps will be described in detail in the following sections.

#### 4.6.1. Select the Pattern Projection Resolution and FRR

A 912×1140 pattern projection at a 60 Hz FRR has been determined. The details can be found in Appendix B.4.1.

#### 4.6.2. Determine HW Operating Clock Frequency and VTC Parameters

The HW operating clock frequency of 86 MHz and the VTC parameters listed in Table A2 have been calculated for the given pattern resolution of 912×1140 @ 60 Hz. This clock rate will be used as the primary clock for the complete HW design. The details of the calculation method can be found in Appendix B.4.2.

#### 4.6.3. Configure the VTC IP

The VTC IP provided by the embedded development SW vendor (i.e., AMD Xilinx) needs to be configured in generation mode because all the parameters were pre-defined for a given resolution and FRR. The details can be found in Appendix B.4.3.

#### 4.6.4. Design and Develop Pattern-Generator HLS IP

AMD Xilinx Vivado SW includes a built-in pattern-generation IP, but it proved to be unsuitable for this specific application for two main reasons. First, it does not incorporate all the necessary elements to produce the required cosine-wave pattern. Second, it is entirely write-protected, preventing any modifications or enhancements. To overcome these limitations and deliver the desired functionality, an HLS-based methodology has been used to create a custom IP.

Since the HDMI IP used requires a 1 PPC rate for the pixel feed, the pattern-generator HLS IP (i.e., Pattern_kernel()) has been structured to allow dataflow modeling through function, loop, and stream pipelining techniques to achieve 1 PPC performance. This IP generates the pattern with a resolution of 912×1140, where the width and height are, respectively, 912 and 1140, as required by the DLP projector. Since the entire HW design has to operate at an 86 MHz clock frequency, the top-level function Pattern_kernel() (i.e., IP kernel) is synthesized as a real-time video-streaming HW IP with an 86 MHz clock.

The AXI4-Lite slave interface exposes the scalar arguments tof_distance, phase, spatial_frequency, throw_ratio, and brightness and the implicit return of the Pattern_kernel IP as memory-mapped registers accessible from the PS (i.e., ARM CPU host). These arguments are therefore designed as runtime parameterizable controls rather than fixed constants, enabling the embedded application in the PS to configure the IP dynamically for different experimental requirements. As a result, the pattern generator functions as a fully controllable parameterized IP core, allowing parameters such as spatial_frequency, tof_distance, and phase, throw_ratio (i.e., to adjust other kinds of HDMI video-mode-supported DLP projector throw ratios) to be adjusted for future experiments without redesigning the HW IP.

In the present study, experimental validation was conducted at a single spatial frequency of $0.122\;{\mathrm{mm}}^{-1}$. The proposed pattern-generator HW IP was intentionally designed as a parameterized and deterministic real-time video-streaming block, in which the exposed runtime arguments, including spatial_frequency, do not alter the underlying throughput of the synthesized design. The current HW IP has been developed and tested using fixed-point arithmetic with sufficient design precision and was parameterized to support spatial frequency values over the range from 0.001 to 0.999 ${\mathrm{mm}}^{-1}$ for future experimental studies. Since IP synthesis maintained 1 PPC operation at the supplied 86 MHz clock for a fixed projector resolution of 912×1140, the generated video stream remains timing-deterministic for a given HW design, independent of the selected spatial frequency parameter within the supported configuration range. In addition, while running in video mode, the DLP projector locks the frequency of the incoming HDMI frame source and maintains its dual-buffer pipeline latency behavior for the received stream, regardless of the frequency of the incoming stream, and it can support up to 120 Hz, according to the manual, while maintaining this dual-buffer latency in a deterministic way. Since in video mode, this projector-side buffering mechanism is determined by the input frame timing rather than by the spatial frequency content of the projected pattern, the projection latency is expected to remain deterministic for different spatial frequency settings as long as the same video timing configuration is preserved. Section 4.1 has already discussed the fundamentals of the dual-buffer latency mechanism.

For a standard 60 Hz video input, one frame occupies approximately 16.67 ms. In video mode, the DLP projector processes the incoming HDMI stream using a deterministic dual-buffer pipeline, in which one frame is loaded into the buffer while another frame is projected through the DMD micromirrors during the same frame period. This timing behavior is deterministic because the projector locks on the input frequency of the HDMI pattern-generator source. Appendix B.1 further explains this behavior for the standard 60 Hz operating condition. In particular, Table A1 shows how the 16.67 ms frame period (i.e., for 60 Hz) is deterministically divided into green, red, and blue bit-plane time slots. Furthermore, Appendix C.3.2 provides further experimental evidence of the same timing behavior for the current pattern-generator IP operating at approximately 58 Hz, where each frame takes 17.26 ms. Under this condition, the projector again maintains the same deterministic dual-buffer operation, where one frame remains in the buffer while another is projected through the DMD micromirrors, and the corresponding bit-plane timing slot distributions are listed in Table A4.

The pseudocode of the dataflow computation model that generates pixel streams in the PL fabric is provided in the Additional Materials, Algorithm A1, and is described in further detail in Appendix B.4.4.

#### 4.6.5. HW Design

The entire hardware design is configured with an 86 MHz clock, which allows it to operate within a single clock domain. Table 1 reports the utilization of post-implementation resources on the xc7z020 SoC. The proposed pattern-generator IP (i.e., *Pattern_kernel*) dominates the most of the utilization of HW resources; using 8634 LUT (16.23%), 7479 FF (7.03%), and 73 DSP48E1 blocks (33.18%). The video subsystem, composed of the *VTC*, *AXI4S-VOut*, and *RGB-to-DVI converter*, contributes only a marginal logic overhead. The remaining control and interconnect infrastructure is grouped into *Others*. In total, the complete design occupies 10,951 LUTs (20.58%), 10262 FFs (9.64%), 73 DSP48E1 blocks (33.18%) and 1 BRAM (0.71%).

The details of the HW design illustration and description can be found in Appendix B.4.5.

#### 4.6.6. Embedded SoC Application (SW)

The embedded SoC application functions as the slave controller, managing both the display and the phase transitions of the projected patterns. The ToF sensor collects measurement data and transmits the readings to the pattern generator running on the SoC. The SoC communicates with the SBC master controller via GPIO and implements a configurable, interrupt-based, frame-level delay mechanism. The details can be found in Appendix B.4.6.

### 4.7. SW System in the SBC Environment

**Figure A7:**
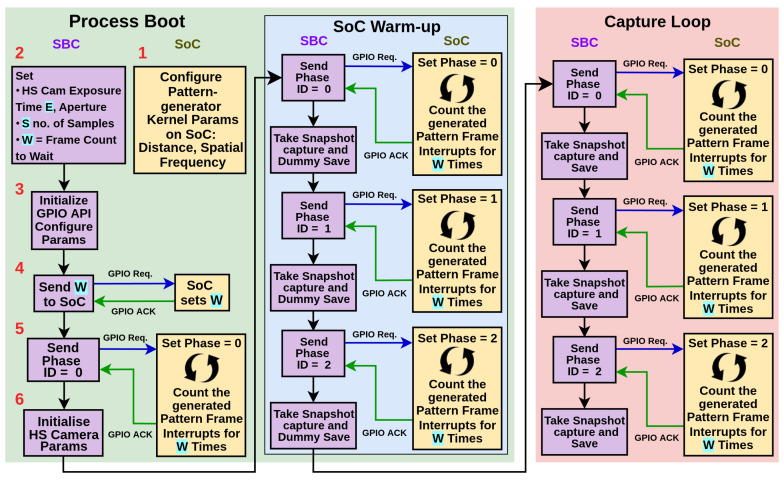
Schematic of the operational workflow.

The SW system in the SBC environment acts as the master controller by controlling the pattern projection on the SoC board through GPIO, taking HS image captures, and finally saving them to the disk. The complete process by which the SBC controls the SoC to achieve projection–capture synchronization is further described in Appendix B.3. Figure A7 in the same section illustrates the operational workflow of the SBC (i.e., the SBC column). This flow is further detailed as a pseudocode in Algorithm A2, along with two stages: boot (Appendix B.5.1) and capture loop (Appendix B.5.2).

### 4.8. Implement Projection–Capture Synchronization

Section 4.2 introduced the projection–capture synchronization concept; this section details its implementation on the SoC board.

Figure 4 shows an abstract diagram of how synchronization can be achieved. The SBC asks the SoC to generate and stream pattern frames for a particular phase ID through a GPIO request using two wires. The SoC board receives this ID through the PMOD port and instructs the Pattern_kernel IP to load the pattern for this particular phase ID. The IP then starts counting the interrupts emitted by the generated frames W times until the pattern frame is initialized and stabilized on the projection surface by the DLP projector. W is sent by the SBC master controller application (implemented through the sendWaitFrameCountToSoC(W) function, which is provided for reference in the (Appendix B.5, Algorithm A2). After that, the SoC board sends an ACK using the other two wires to the SBC. After receiving the ACK, SBC takes an HS image capture.

**Figure 7:**
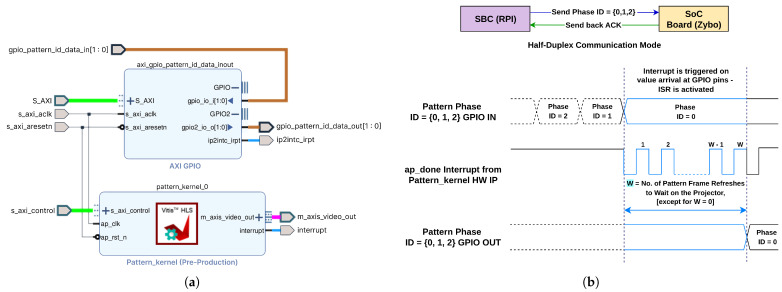
Implementation of the projection–capture synchronization: (**a**) Zoom-in view of the Pattern_Change_Handler group from the HW design. (**b**) Abstract timing diagram of the SoC’s inner interrupt-handling mechanism for the W parameter.

**Figure A8:**
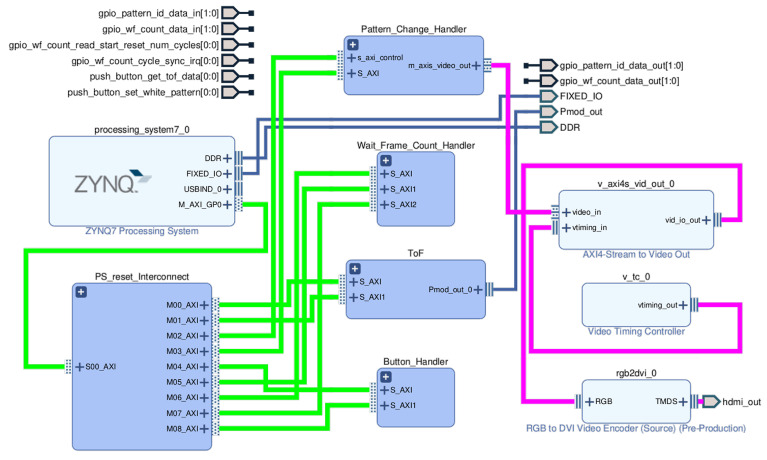
The interface view of the HW design.

To control and count the generation of individual pattern frames, the SW system running on the SoC PS must know when a pattern frame is generated by the Pattern_kernel IP. To achieve this, an interrupt must be sent from the Pattern_kernel IP to the PS IP after the completion of one-frame generation. Figure 7a zooms in on the Pattern_Change_Handler group of the HW design (provided as a reference in the Appendix B.4.5, Figure A8, for further explanation), where the interrupt port can be seen on the Pattern_kernel IP. The interrupt port is connected to the PS. Also, the AXI GPIO IP from this group is responsible for the GPIO connection with the SBC GPIO pins through the SoC board’s PMOD port. The interrupt mode is enabled on the GPIO IP as well.

On the SoC board, the receiving GPIO pair for the phase ID is grouped into Channel 1, and the ACK sending pair is Channel 2. A separate pair of GPIO pins is implemented so that receiving and sending do not conflict with each other, ensuring technical simplicity.

Figure 7b also shows that a simplified GPIO dataflow occurs between the SBC and the SoC, while interrupt-signal management takes place inside the SoC. It describes how different values of W (except W = 0) are managed with respect to the interrupt-handling mechanism. The SBC transmits an integer phase ID = {0,1,and2} as the binary bit combination of 00, 01 and 10; each bit is sent over a pair of wires. The SBC can send only one phase ID at an arbitrary moment, which means that a request can occur at any moment in time. As complete communication between the SBC and SoC takes place in half-duplex mode, after making this GPIO request, the SBC waiting period begins. When the AXI GPIO IP (Figure 7a) sees changes in the PMOD pins of Channel 1, it emits an internal interrupt. This interrupt causes the execution of an ISR as a callback, as given in the self-documented pseudocode in Algorithm 1.

The SBC writes to its own GPIO pins sequentially. The first write immediately triggers an interrupt on the SoC and invokes the ISR. A static delay of 8 ms has been added on line 3 (i.e., WAIT(8 milliseconds)) so that the SBC can complete the writes to both pins. This static delay was found through trial and error. Every time the Pattern_kernel IP completes its execution, it emits an ap_done interrupt. As the IP is always running, the interrupt signal will always be high, as can be seen in the timing diagram in Figure 7b. Line 11 of the ISR Algorithm 1 cleans that first. So in the timing diagram, it can be seen that it goes down at the beginning, as shown by the signal at the ap_done interrupt from the Pattern_kernel HW IP. Line 12 has the for loop that runs for W tripcounts.

The Pattern_kernel IP is controlled from the PS (i.e., from the ISR) through IP control registers. Different control registers are checked by the IP at the IP boot and runtime to perform different control tasks. At line 7, DISABLE_AUTO_RESTART(pattern_kernel) writes a Boolean false value to the Pattern_kernel IP’s auto-restart control register. Once the existing frame (i.e., the previous phase before changing to the new phase) finishes, the Pattern_kernel IP sees the false value and stops auto-restarting the IP. Next, at line 8, the IP control registers are updated with the new phase ID as the SBC requested through the GPIO. At line 9, auto-restart is enabled again. This causes the IP to begin generating frames for the new phase ID. All three writes to the correspondent registers complete in microseconds, and this delay is considered negligible in performance measurements.
**Algorithm 1** Handle_Phase_Id_Change_Request ISR  1: **function**
Handle_Phase_Id_Change_Request  2:     // 1. Wait to ensure both input pins have settled  3:     WAIT(8 milliseconds)  4:     // 2. Read the new pattern ID from GPIO channel 1  5:     receivedPhaseId ← READ_GPIO(channel = 1)  6:     // 3. Update the pattern generator IP  7:     DISABLE_AUTO_RESTART(pattern_kernel)  8:     SET_PHASE(pattern_kernel, phases[receivedPhaseId])  9:     ENABLE_AUTO_RESTART(pattern_kernel)
10:     // Clear any stale “done” flags and then wait for W frame count 11:     CLEAR_INTERRUPT(pattern_kernel, AP_DONE_INTERRUPT_MASK) 12:     **for** i←0
**to**
W−1 **do** 13:         **while** (GET_INTERRUPT_STATUS(pattern_kernel) & AP_DONE_INTERRUPT_MASK) ≠ AP_DONE_INTERRUPT_MASK **do** 14:            // wait until AP_DONE interrupt is emitted 15:         **end while** 16:         CLEAR_INTERRUPT(pattern_kernel, AP_DONE_INTERRUPT_MASK) 17:     **end for**
18:     // 5. Send the acknowledgment back on GPIO channel 2 19:     WRITE_GPIO(channel = 2, value = receivedPhaseId) 20:     // 6. Finally, clear the GPIO interrupt flags 21:     CLEAR_GPIO_INTERRUPT(mask) 22: **end function**

Since the SBC phase-change request can arrive through GPIO at any moment, even while the IP is still generating the frame of the previous phase, an interrupt (possibly residual) could be emitted when this last frame finishes. This approach guarantees that the pattern stream never halts, at the minor cost of measuring one interrupt from the old phase. This phenomenon was investigated and verified through an experiment, as described in Section 5.5.3. Subsequently, as presented in the same section, the first interrupt period measurement equations were derived and calculated using the runtime performance data across different W configurations.

At line 13, the while loop checks for the ap_done interrupt coming from the Pattern_kernel IP. Then the ap_done interrupt register is cleared again so that, in the next for loop iteration, it can be checked again. This is why in the timing diagram Figure 7b, a repetitious pattern of the square wave can be seen to occur W times as the for loop runs for W tripcounts. After finishing the for loop, the received phase ID is written as an ACK value to GPIO Channel 2 at line 19. Finally, the AXI GPIO interrupt is cleared at line 21 so that it can be ready to detect the interrupts when the next phase ID arrives. For the case of W = 0, the ISR skips the entire for loop and jumps directly to line 19 to send the ACK.

The GPIO API in the SBC can detect changes in GPIO ACK pins that are connected to Channel 2 of the SoC board by continuous polling. After receiving the ACK, the SBC takes and saves the HS image capture and prepares to send the next phase ID.

## 5. Results

This section presents the experimental methodology and results as a structured workflow based on the evaluation block of the development methodology (Figure 6). It begins with Section 5.1, which details the experimental setup, including room preparation, parameter tuning for pattern generation, camera configuration, and determination of exposure settings through white calibration for both polarizer and no-polarizer cases. Section 5.2 examines the quality of the generated patterns, as well as the runtime performance of the pattern-generation process. Section 5.3 describes the capture process, including acquisition of the white reference, preparation of the target sample, and capture of the reference and the final sample. Section 5.4 explains post-capture steps such as band selection, preprocessing, and phase-plot generation. Finally, Section 5.5 defines the demodulation evaluation criteria, compares the SOTA and proposed system outputs with reference data, experimentally validates frame-level synchronization, conceptualizes the frame-sequence timeline concept with respect to the camera exposure period to establish a phase-synchronization and projection analysis framework, and presents the performance comparison between the two systems.

### 5.1. Prepare Experimental Environment

Several steps must be completed before SI data acquisition: prepare the experimental room, set pattern-generation parameters, evaluate pattern-generation quality and performance, and finally determine the HS camera exposure periods considering two settings: polarizer and no polarizer.

#### 5.1.1. Set Pattern-Generator Parameters

For experimental integrity, both the proposed HyperSI system and the SOTA HSPy-SI system used the exact same pattern-generation parameters. The parameters included the distance between the projector and the projection surface, phase, spatial frequency, throw ratio, and brightness. Each of the SI systems employs a different ToF sensor model to measure the distance from the camera and projector to the projection surface. Hence, a static distance of 445 mm was used for all experiments since it fully covers the FOV of the target sample. The spatial frequency was set to $0.122\;{\mathrm{mm}}^{-1}$. This choice was motivated by the fact that a SOTA commercial biomedical SFDI system, Modulim Clarifi^®^ (Modulim, Costa Mesa, CA, USA), also uses a single spatial frequency of $0.12\;{\mathrm{mm}}^{-1}$ for tissue imaging [28]. Since both systems use the same DLP projector, the throw ratio was set to 1.2 for the used projector model [33]. Therefore, the complete set of pattern parameters is defined as follows: three phases (i.e., 0°, 120° and 240°), spatial frequency of $0.122\;{\mathrm{mm}}^{-1}$, distance of 445 mm, brightness of 255, and throw ratio of 1.2.

#### 5.1.2. Configuration of the HS Snapshot Camera

The aperture was set to ≈f1.65 (i.e., the maximum) to allow more light to reach the sensor, which helps reduce exposure time and, consequently, overall acquisition time. The gain was kept at the default value of 0.0. To determine the appropriate exposure time, the method recommended by the Ximea HS camera manufacturers was followed, which involves a white calibration process [40]. This process includes collecting exposure time through multiple trial captures of a white ceramic target until the measured pixel values are 80–90% of the maximum pixel value (i.e., 255 for 8-bit capture). However, since an LLG was used to transmit the maximum amount of light from the light source to the DLP projector, overexposure may result in pixel saturation (i.e., produce a burned-out appearance in some image segments), particularly in the no-polarizer configuration of the HS camera. Hence, 80% of the maximum value of the pixels was selected to avoid overexposure. To perform this calibration, a white ceramic target (SphereOptics GmbH, Herrsching am Ammersee, BY, Germany) was first placed on the projection bed. A white image (i.e., all pixel values set to 255, size 912×1140) was generated on-the-fly and used as a virtual filter, allowing the projector to transmit light through it to the target. Both systems support this dynamic filter creation, ensuring uniform illumination of the ceramic and maximum reflected light. Finally, following the aforementioned method, the white ceramic captures were repeated with and without polarizers (polarizers have been described in Section 3.1), and the optimal exposure times were determined to be 213 ms for the polarizer and 39 ms for the no-polarizer setting.

### 5.2. Evaluate Pattern-Generation Performance

**Figure 8:**
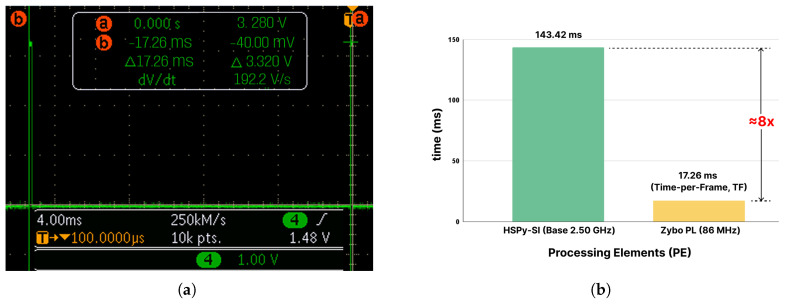
Pattern-generation performance: (**a**) HyperSI FRR performance measured with oscilloscope (Figure A8). (**b**) Comparison of time taken to generate each frame.

Assessing the quality of pattern generation is critical, as it directly influences the spatial frequency of the pattern and, consequently, the quality of demodulation in any SI system (Section 1). This section evaluates whether the HyperSI system meets the objective of generating high-quality 24-bit RGB modulated patterns with high performance (≈60 Hz). High quality refers to the production of pixel values that are identical to or negligibly deviate from those of the reference SOTA HSPy-SI system, while high performance means achieving a frame-generation rate close to 60 Hz. To assess these two aspects, Section 5.2.1 compares the generated patterns with those obtained with the SOTA system to verify pixel-level consistency. Section 5.2.2 then measures the FRR and compares it with that achieved by the SOTA implementation. Based on these evaluations, it was found that the pattern generator of the proposed system successfully meets both objectives (Figure 8).

#### 5.2.1. Evaluate the Quality of Generated Patterns

Quality was evaluated using the Root Mean Square Error (RMSE) of all pixel values for the three different phases from the HSPy-SI system versus those from the proposed system. An Intel processor, used in the SOTA HSPy-SI system, computed cosine values with 64-bit precision, producing reference pixel values for comparison. The results indicate that the pixel values of the HyperSI system exhibit 0% absolute variance across the 0–255 range compared to the SOTA system. Therefore, it can be concluded that the generated pixel values are identical and can be considered high-quality.

#### 5.2.2. Evaluate the Runtime Performance

Measuring the exact FRR of the SOTA system for pattern generation is challenging due to the abstraction layers of the OS and the complexity of the system. To address this, the Python (v3.8.10) implementation of Equation (5) was timed over ten executions, yielding an average frame period of 143.42 ms. To measure the FRR of the HyperSI system’s Pattern_kernel HLS IP, the VSync signal period was measured. This signal, extracted from the output of the AXI4S-VOut IP core, was routed through a PMOD connector and observed with an oscilloscope. A screenshot of the measurement is shown in Figure 8a. This demonstrates a consistent frame period TF of ≈17.26 ms, corresponding to an FRR of 57.94≈58 Hz, which is closely aligned with the theoretical value $FRR_{actual\_cvt}=59.78$ Hz (Section 4.6.2). And finally, the performance of both systems is plotted in Figure 8b in terms of time taken for each frame, showing more than ≈8× improvement.

### 5.3. Perform Experiments

The HS camera has been configured with two exposure settings: 213 ms with a polarizer and 39 ms without a polarizer (i.e., no-polarizer setting) (Section 5.1.2). Since the SOTA HSPy-SI system has been configured to operate with a polarizer (Section 3.3), all SOTA captures employ an exposure time of 213 ms. However, the proposed system was evaluated under both settings: 213 ms with a polarizer and 39 ms with no polarizer. Hence, both systems share two common parameters: E, the exposure time, and S, the number of modulated sample captures. For this study, S = 30 was chosen to approximate continuous real-time acquisition, simulating a typical hospital environment, where manual user intervention during image capture is generally impractical. However, the SOTA HSPy-SI system is limited to performing only three phase-modulated captures followed by demodulation at a time. Hence, the capture session for the SOTA system had to be repeated 10 times to match the total number of modulated captures S = 30 so that the performance of the runtime could be compared with that of the system proposed in Section 5.5.14. In addition, for three-phase demodulation, the S must satisfy

S≥3andS≡0(mod3).

The proposed system has a third parameter, W, which is tuned with four possible values 0, 1, 2, and 3 to experiment with different waiting periods. Acquisition sessions are labeled by their settings; e.g., the SOTA session for E = 213, S = 30 is denoted by E-213-S-30, while the proposed system session with E = 213, W = 2, S = 30 is denoted by E-213-W-2-S-30. Since demodulated images are the output of three modulated captures of consecutive phases, each group of three modulated captures can be denoted as a group, i.e., g-0, g-1, g-2, and so on. Before acquiring the final target samples, three acquisition steps must be performed. First, demodulated white reference images are obtained to support the preprocessing of the reference and final target sample images (this process is explained further in Appendix C.1.1). Second, the target sample to be captured is prepared (Section 5.3.1). Finally, both the reference and the final modulated target sample are acquired (Section 5.3.2), where the reference captures serve as the baseline for comparison and evaluation of the final data.

#### 5.3.1. Prepare a Target Sample for Capture

A 3D-printed four-chamber sample box containing salt, cocoa, coffee, and sugar was used as a consistent target to ensure uniform testing. To compare and evaluate modulated captures with reference captures, artifact-free demodulated images and accurate phase shifts are required. A white paper background with a gap allowed the extraction of a clean sine-wave cycle for analysis.

Subsequently, a phase plot (PP) was generated from the clean sine-wave cycle by mapping pixel intensities across approximately one sine-wave period. Applying the smoothing Equation (9) produced a smoothed phase plot (SPP), producing cleaner and higher-contrast signals for accurate phase-shift evaluation relative to the reference (Section 5.4.2).

The details can be found in Appendix C.1.2.

#### 5.3.2. Capture Reference and Final Modulated Target Sample

Reference modulated captures were obtained to establish a ground-truth reference. For each exposure time (i.e., polarizer setting as defined in Section 5.3), each of the three phase-shifted modulated patterns projected on the target sample box with white paper underneath was captured separately without any synchronization. Finally, all reference modulated captures were demodulated using Equation (6).

The HS snapshot camera used in this work captures a 5 × 5 spectral mosaic as a single 2D sensor image with a resolution of 2045 × 1085. Further details can be found in Appendix A.1. During HS cube formation, the 25 spectral bands are separated using the fixed mosaic mapping. Therefore, TPD is performed using spatially corresponding pixels across the phase captures, and no additional spatial information is lost during the demodulation step due to pixel-to-pixel mapping.

Acquisitions were conducted under two experimental settings: (i) with polarizers, using E-213-S-30 for the SOTA HSPy-SI system and E-213-W-0,1,2,3-S-30 for the HyperSI system, and (ii) without polarizers (i.e., no-polarizer setting for low-exposure condition), using E-39-W-0,1,2,3-S-30 for the HyperSI. Since the SOTA HSPy-SI system was designed for the polarizer setting (i.e., high exposure time), no captures were taken by the SOTA HSPy-SI for E-39 (Section 3.3). All runtime performance metrics, such as Actual Capture Time (ACT), Change Pattern Time (CPT), Saving Time (ST), and Total Time (TT), were automatically collected by both systems during system runtime. They will be described and analyzed in detail in Section 5.5.14 with respect to the real-time measures found after the capture runtime.

The details can be found in Appendix C.1.3.

### 5.4. Prepare Captured HS Image Dataset for Evaluation

Detailed analysis of all 24 spectral bands of the HS camera (Section 3.1) in this study would be overwhelming. Moreover, low-energy responses in certain bands can also introduce artifacts. Therefore, a subset of seven bands was first selected as the Spectral Bands of Interest (SBIs) through an experiment for a focused analysis (Section 5.4.1). The selected bands then underwent preprocessing, demosaicing, and phase-plot generation prior to final demodulation evaluation. From this subset, four bands with clean, high-contrast signals were chosen (Section 5.4.2) for a detailed evaluation analysis (Section 5.5).

#### 5.4.1. Experiment for the Initial Selection of the SBI

The projector used in this study supports fiber-coupled broadband illumination, allowing external broadband light to be delivered into the projection engine. This configuration enables visible to near-infrared (NIR) structured-illumination experiments within the supported spectral range of approximately 420 nm to 850 nm. In this projector version, the standard DMD is replaced with an IR-compatible DMD. The IR DMD includes a window coating that improves efficiency in the infrared range, providing approximately 11% additional efficiency over the 420 nm to 850 nm range. Therefore, the projector light engine is suitable for NIR broadband SI experiments.

Analysis and evaluation of all 24 spectral bands is impractical; therefore, a smaller subset of bands needed to be selected through preliminary experimentation. A miniature spectrometer (Ocean Insight, Orlando, FL, USA) was used to analyze the spectral response of the light source and the projector, both with and without a polarizer. The Ocean HDX-VIS-NIR spectrometer has an optical resolution of 0.70 nm FWHM (typical), a signal-to-noise ratio of 400:1, and a dynamic range of 12,000:1. The spectral range is obtained with 2067 measurements, averaged over the entire spectral range using a 10 μm slit. Moreover, the spectrometer measurement was conducted with a fiber optic positioned orthogonally to the polymer reference at 5 cm from the projector, following a similar methodology to that used in the literature [51].

**Figure 9:**
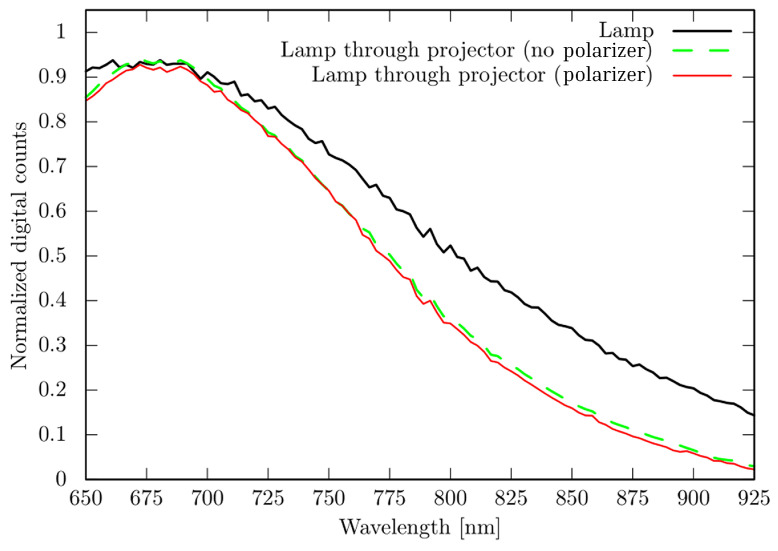
Spectral intensity responses of the lamp and DLP projector (with and without polarizer), measured from 650 to 925 nm using a miniature Ocean Insight VIS–NIR spectrometer (16-bit ADC).

The results plotted in Figure 9 show that the projector attenuates the energy coming from the lamp beyond 700 nm. As a result, the signal strength dropped noticeably throughout the subsequent contiguous spectral bands. Therefore, the initial seven bands within the range 650–775 nm were selected for further analysis, as they have a minimum energy response of 50% or higher. These selected bands will be further reduced to four bands in Section 5.4.2 for ease of evaluation.

The details of the experiment are explained further in Appendix C.2.1.

#### 5.4.2. Preprocess, Demosaic, and Generate Phase Plots of Demodulated HS Images

**Figure A9:**
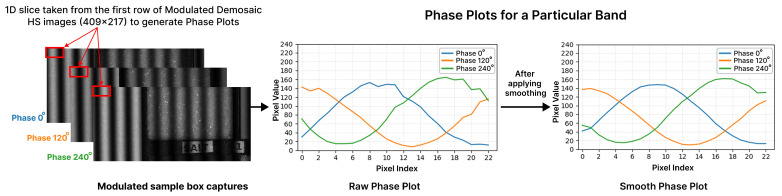
The method for preparing the sample box and collecting SPPs: placing white paper under the sample creates a left-edge FOV gap, allowing the extraction of a pixel row spanning one sine-wave cycle; its raw intensities are then smoothed using Equation (9) to generate an SPP.

All demodulated HS images were first preprocessed through calibration (Equation (7)) and spectral correction (Equation (8)) and then demosaiced to reconstruct spectral cubes of size 409×217×24 (665–960 nm). The SPP preparation method (further explained in Appendix C.2.2, Figure A9) was applied to preprocessed modulated captures for both reference and experimental configurations. From the initial seven bands in the 650–775 nm range, four bands (714 nm, 730 nm, 742 nm and 755 nm) were chosen for the final phase-shift evaluation due to their strong visual contrast. Beyond 775 nm, artifacts appeared as the signal dropped from about 50% to 5%, making higher-wavelength bands unreliable.

More details can be found in Appendix C.2.2.

### 5.5. Evaluation of the Demodulation

Section 5.5.1 first defines the criteria for evaluating the demodulated images produced by the SOTA HSPy-SI and HyperSI systems. Reference captures from Section 5.3.2 are used for evaluation. The captures performed with the SOTA HSPy-SI system are evaluated in Section 5.5.2 with respect to the criteria defined previously. Subsequently, a detailed experimental analysis is performed in Section 5.5.3 to validate frame-level synchronization in the proposed HyperSI system. Since phase alignment requires the interrupt period to match the frame period (i.e., I≈TF), the experiment measured the actual interrupt arrival periods and examined their behavior with respect to the W parameter. The results confirm that the interrupt period is consistently 17.26 ms (i.e., I≈TF≈17.26 ms). And the results also validate the design choice made in Section 4.8 for the first interrupt originating from the frame of the previous phase. Before proceeding to the evaluation, it is essential to establish a phase-synchronization and projection analysis framework, as this is a prerequisite to explain all key events across the SBC, SoC, DLP projector and camera together in a unified manner. To define this framework, a frame-sequence timeline concept is introduced in Section 5.5.4 using the case of W = 1 as an example. Section 5.5.5, Section 5.5.6, Section 5.5.7, Section 5.5.8, Section 5.5.9, Section 5.5.10, Section 5.5.11 and Section 5.5.12 demonstrate that the quality of the demodulation improves as the W value increments by one count, eventually reaching an acceptable optimal tuning point. Two experimental camera exposure configurations are evaluated: no polarizer (i.e., 39 ms exposure) and with a polarizer (i.e., 213 ms exposure). These sections employ the frame-sequence timeline concept (Section 5.5.4) to show what the camera records from the projection surface during its exposure window relative to the pattern frame generator, thus clarifying the sequence of events that lead to the resulting demodulation quality. Section 5.5.13 presents the derivation of equations for the minimum required W to ensure phase alignment and stable projection timing between the SoC and the DLP projector. Finally, Section 5.5.14 evaluates the runtime performance of both systems.

#### 5.5.1. Definition of the Demodulation Criteria

Demodulation is considered acceptable only if both of the following criteria are met:

1. A demodulation is accepted if at least two of the pre-selected bands exhibit phase shifts in the modulated images that closely align with the reference vertical guides of the reference SPP. It is rejected only if the phase shifts show a significant and uniform deviation from the reference across the evaluated bands.
2. The demodulated images should remain free of noise and artifacts.

**Figure A13:**
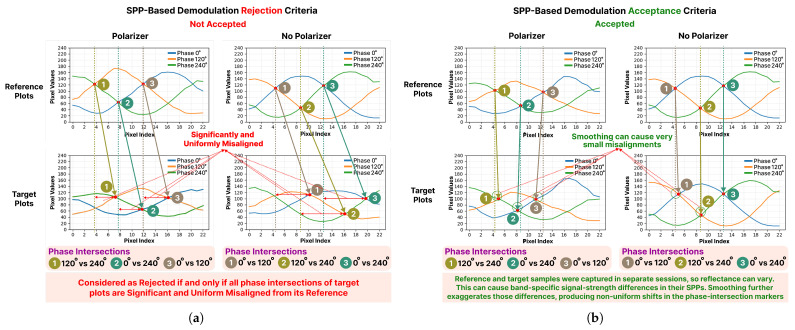
Demodulation acceptance criteria based on the SPP, with three vertical guides marking intersection points at 0° and 120°, 120° and 240°, and 0° and 240°. (**a**) Large misalignments (marked by red arrows) at all intersection points in the target plot relative to the reference are considered unacceptable. (**b**) Small deviations at intersection points in the target plot (marked by downward-pointing arrows and green circles) due to the smoothing approximation compared to the reference are considered acceptable.

**Figure A14:**
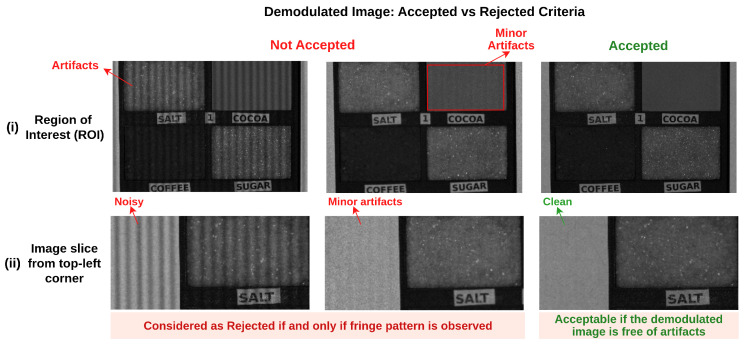
The demodulation acceptance Criterion 2 based on assessing image quality through visual inspection of the demodulated results. Row (**i**) highlights the region of interest (ROI) of the sample box, while row (**ii**) shows a slice from the top-left corner of the demodulated image, where white reference paper has been used to make minor artifacts clearly visible across the different substances (salt, cocoa, coffee, and sugar).

These conditions are further explained in Appendix C.3.1, Figure A13 and Figure A14.

#### 5.5.2. Demodulation Evaluation of SOTA HSPy-SI System

**Figure 10:**
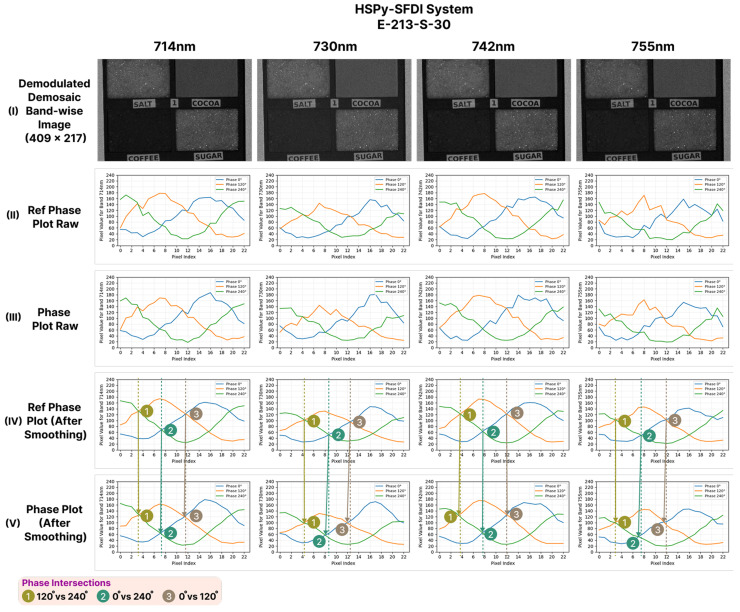
The figure shows the demodulated capture by the SOTA HSPy-SI system for the E-213-S-30 configuration, along with its phase-shift comparison against the reference. The four spectral bands (714 nm, 730 nm, 742 nm, and 755 nm) are arranged in columns.

Figure 10 illustrates the demodulated capture of the SOTA HSPy-SI system for the E-213-S-30 configuration, along with its phase-shift comparison against the reference. Row I presents the demodulated and demosaiced images (i.e., 409×217), which exhibit clean demodulation without any visible noise. Rows II and III display the phase profiles (PPs) for the reference and captured images, respectively. In rows IV and V, the phase shifts indicated by markers 1, 2, and 3 (Row V) align closely with the corresponding reference spatial phase profiles (SPPs) shown in Row IV.

**Figure 11:**
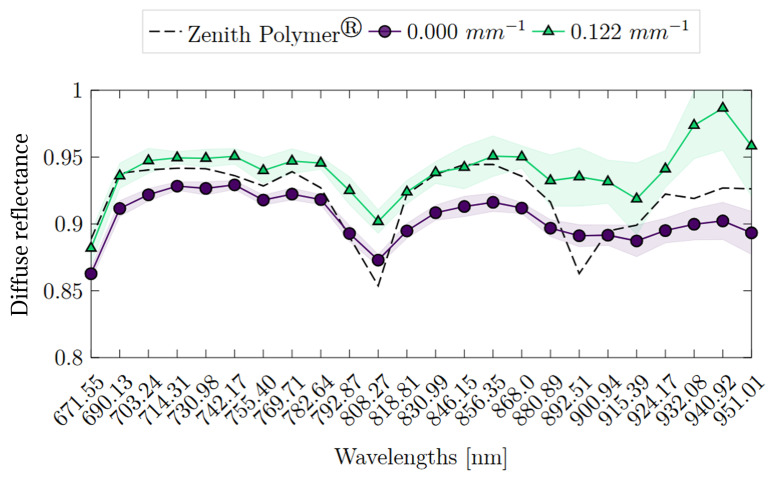
Reflectance spectral signatures when the Zenith Polymer Reflectance Standard was illuminated with different spatial frequencies. Mean spectral signatures were obtained using a 25 by 25 pixel region of interest centered in the polymer. The shaded regions represent the standard deviations of the measurements.

However, the 730 nm band exhibits reduced signal strength and slight marker shifts compared to the other three selected wavelengths (i.e., 714 nm, 742 nm, and 755 nm). This behavior was consistently observed across multiple trial captures using both the SOTA system and the proposed HyperSI synchronizer. After applying the smoothing approximation to generate the SPPs for the 730 nm band, the effect becomes more prominent, as smoothing tends to accentuate the relative shift of the markers compared to the reference SPPs. Hahn et al. reported a detailed characterization of a previous mosaic-based HS snapshot camera from the same manufacturer [52]. They concluded that the correction matrix provided by the manufacturer is insufficient to reconstruct the spectrum without introducing large measurement errors. Furthermore, the irregularities found in the sensor were present across the whole sensor and, hence, in the entire spectral range of the camera. Although Hahn et al. propose to create an individual matrix after characterizing the camera, a dedicated optical system is required. Although Hahn et al. characterized a previous version of the snapshot camera used for the proposed system (Ximea MQ022HG-IM-SM5X5-NIR, compared to MQ022HG-IM-SM5X5-NIR2), yet a dedicated optical system is needed, which we do not have access to (e.g., a tunable laser, high resolution monochromator, and a calibrated detector array). Consequently, a full re-characterization of the spectral response of the camera is beyond our current experimental capabilities. Nevertheless, the spectral response was validated by imaging a reference polymer with a known, smoothly varying reflectance spectrum. The polymer used is the Zenith Polymer^®^ Reflectance Standard, which exhibits nearly ideal Lambertian diffuse reflectance of 99% (SphereOptics GmbH, Herrsching am Ammersee, BY, Germany) and is represented with a dashed black line in Figure 11.

Figure 11 shows the reconstructed spectra when projecting with the spatial frequencies $f=0.000\;{\mathrm{mm}}^{-1}$ and $f=0.122\;{\mathrm{mm}}^{-1}$, purple and green in color, respectively. As expected, the highest wavelengths produce greater error, as expressed by the higher standard deviation obtained, which aligns with the measurements observed regarding the spectral analysis conducted with the spectrometer and the energy coming from the projection system.

**Figure 12:**
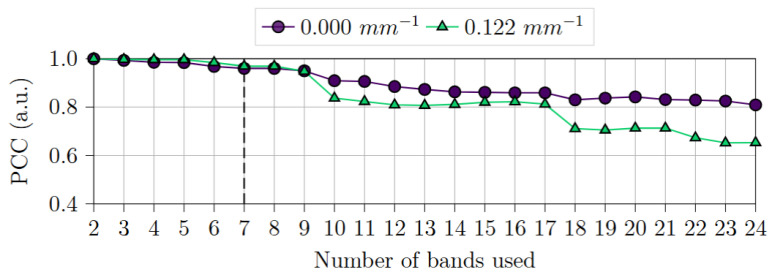
Reflectance spectral signatures when the Zenith Polymer Reflectance Standard was illuminated with different spatial frequencies. Mean spectral signatures were obtained using a 25 by 25 pixel region of interest centered in the polymer. The shaded regions represent the standard deviations of the measurements. The vertical dashed line indicates the wavelength (769.71 nm) cutoff selected for the experiments in this study.

Moreover, Figure 12 shows the Pearson Correlation Coefficient (PCC) computed for each spatial frequency used with the SOTA HSPy-SI system. The results indicate 80% and 63% agreement with the reference when 24 bands are used, including 730 nm, for the $f=0.000\;{\mathrm{mm}}^{-1}$ and $f=0.122\;{\mathrm{mm}}^{-1}$ spatial frequencies, respectively. However, reducing the number of bands produces higher PCC values, with values close to 97% for both spatial frequencies when using seven bands, confirming that the measurements taken at lower wavelengths provide reliable measurements after calibration. Nevertheless, these variations do not affect the quality of the demodulated images. Moreover, such small, random (i.e., non-uniform) marker shifts are considered acceptable under the demodulation evaluation criteria (explained further in the Appendix C.3.1, Figure A13b). Therefore, a detailed investigation of this behavior is beyond the scope of the present study.

In conclusion, the demodulated images remain free of artifacts across all four bands, and the SPP markers show strong alignment with their reference counterparts. According to the demodulation criteria defined in Section 5.5.1, the SOTA HSPy-SI system satisfies all the required conditions and is therefore accepted.

#### 5.5.3. Validation of Frame-Level Synchronization in HyperSI System

To ensure accurate phase alignment in the proposed HyperSI system, it is first necessary to validate frame-level synchronization. Phase alignment ensures that the pattern phases generated by the SoC and the phases displayed by the DLP projector are synchronized at the frame level. To confirm phase alignment, it is necessary to verify that the interrupt period matches the frame period of the projected frames (i.e., I≈TF). Furthermore, since the key configuration parameter of the proposed HyperSI system is the frame-based interrupt count W, which introduces a configurable delay to compensate for projector latency, the accuracy of W directly depends on the interrupt timing behavior. Therefore, it is essential to measure and analyze the actual interrupt arrival period, as well as to validate the design choice (Section 4.8) of the first interrupt originating from the frame of the previous phase.

The HW was designed with the specific objective of maintaining the equality between the interrupt period and the frame period. An experiment was conducted to study how the frame-based interrupt count W parameter is affected by the interrupt timing behavior. The interrupt period *I* must match the measured frame period TF≈17.26 ms (i.e., I≈TF≈17.26). An SW timer was added to the SoC application to measure the interrupt period during phase transitions. The system was initialized to the stream phase P0, and a modified SBC master controller application triggered five interrupts (W = 5) to switch from P0 to P1 in six random trials. These arbitrary trials were performed to observe real interrupt timing behavior and detect inconsistencies between interrupts. The measurements showed a consistent interrupt period of 17.255 ms (approximately 17.26 ms, averaged over 24 measurements). In addition, it was also observed that the first interrupt always originated from the frame of the previous phase, an outcome of the design choice implemented in the ISR Algorithm 1 (Section 4.8). The details of this experiment are provided in Appendix C.3.2, Table A3.

**Figure 13:**
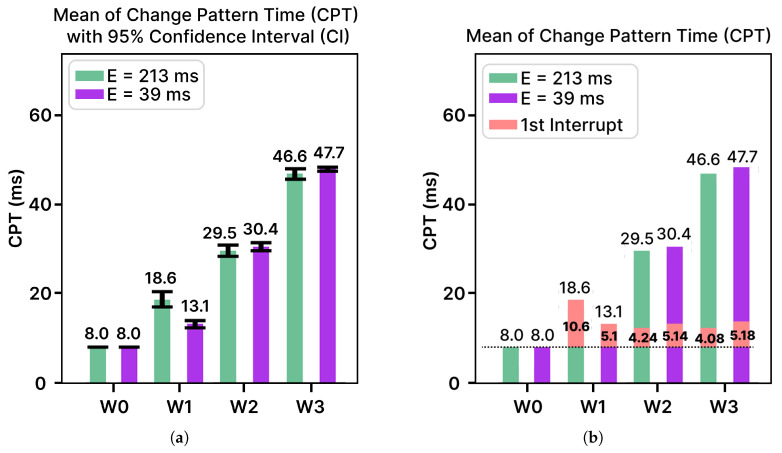
Measured mean CPT vs. W plots for all real-time HyperSI captures across all experimental configurations. (**a**) Mean CPTs with 95% CIs. (**b**) Stacked bar chart of mean CPTs, with the portion corresponding to the measurement of the first interrupt highlighted in red.

CPT is the delay (e.g., in milliseconds) before taking a capture. All CPT values for the different experimental configurations were collected during data capture (Figure 13a). In the aforementioned experiment, it was observed that the first interrupt $I_{1st}$ always originates from the frame of the previous phase. Hence, measuring $I_{1st}$ for the final captures is necessary for two reasons. First, it reconfirms the finding from the previous experiment that the first interrupt always originates from the frame of the previous phase. Second, it serves as a prerequisite for accurately characterizing the frame sequence generated by the SoC and managed by the DLP projector, along with their precise timing behavior (Section 5.5.5, Section 5.5.6, Section 5.5.7, Section 5.5.8, Section 5.5.9, Section 5.5.10, Section 5.5.11 and Section 5.5.12). Therefore, a generalized equation to define the CPT (Equation (A14)) is required so that the corresponding general equation to measure $I_{1st}$ can be derived thereafter.

CPT should be measured as the time taken to execute the ISR (Algorithm 1) on the SoC board; i.e., the W count is factored with the TF into the CPT, including an SD of 8 ms (Section 4.8). The SD is the period implemented to stabilize the GPIO communication between the SBC and the SoC. For W = 0, no interrupts are counted. However, for W≥1, the interrupt count begins. Each interrupt interval $I_{i}$ approximates the TF (i.e., TF=17.26 ms), and hence, $I_{1}\approx I_{2}\approx I_{3}\approx \ldots \approx TF\approx 17.26$ ms. Therefore, CPT can be expressed in closed form by Equation (10).

(10) $$CPT\approx SD+I_{1st}+I_{2nd}+\ldots +I_{Wth}\;\;\;\left[\mathrm{where};W\geq 1,I_{1}=I_{1st},I_{2}=I_{2nd}\ldots ,I_{W}=I_{Wth}\right]$$

Using Equation (10), $I_{1st}$ can be measured from the CPT using Equation (11).

(11) $$I_{1st}\approx CPT-SD-\left(TF\times \left(W-1\right)\right)\;\left[\begin{array}{c} \mathrm{Sin}\mathrm{ce}\;\mathrm{LHS}\;\mathrm{computes}\;I_{1st},\;\mathrm{RHS}\\ \mathrm{excludes}\;\mathrm{one}\;\mathrm{interrupt}\;\mathrm{count}\;\mathrm{thus},\;W-1 \end{array}\right]$$

Finally, the values computed for $I_{1st}$ in different experimental configurations in the final capture confirm the findings of the previous experiment. For all W = 1, 2, 3 configurations, $I_{1st}<TF$ was observed, demonstrating that the first interrupt consistently originates from the frame of the previous phase ID. The means of the measured CPT values and their corresponding $I_{1st}$ values (using the general Equation (11)) were computed. The results are expressed in milliseconds and plotted with 95% confidence intervals (CIs). These plots are presented in Figure 13a,b for all experimental capture configurations.

The larger $I_{1st}$ values (i.e., 10.6 ms for the 213 ms exposure vs. 5.1 ms for the 39 ms) for the case W = 1 in Figure 13b are due to the large difference between the two tested camera exposure times, i.e., 213 and 39 ms. In each acquisition loop, the system first changes the pattern and then captures it using the selected exposure time. Since the pattern generator continues streaming frames during the exposure, the loop reaches the next pattern-change event at a different moment in the ongoing frame cycle depending on the exposure duration. For this reason, the first detected interrupt can appear at a different time point within the previous-phase frame period, leading to the observed difference in $I_{1st}$. This effect is expected when exposure times differ significantly (i.e., 213 − 39 = 174 ms), and the proposed design still guarantees that $I_{1st}$ remains below the measured frame period’s TF.

The detailed derivation of the CPT and $I_{1st}$ equations, along with the computation of $I_{1st}$ for different experimental configurations, is further provided and explained in Appendix C.3.2.

#### 5.5.4. Framework for Phase Synchronization and Projection Analysis

The HyperSI system operates as a tuner-based, interrupt-driven synchronizer that implements configurable frame delays through the configurable W parameter. For an in-depth demodulation evaluation of the modulated captures corresponding to each W configuration, four key processes must be considered together:

- The behavior of the system when the phase-change request is sent from the SBC to the SoC while the SoC is generating frames;
- How the DLP projector receives and manages these frames within its internal dual buffer;
- The reorientation and stabilization behavior of the DMD micromirrors for each frame;
- How the camera perceives and records the projected frames.

To unify these processes under a single analytical perspective, a frame-sequence timeline is introduced to establish a phase-synchronization and projection analysis framework that ensures that the demodulation evaluation accounts for every key event occurring during the experimental capture. This timeline provides a unified representation of how all components interact across phases for any W configuration. This section discusses all these processes together using one representative configuration, W = 1. The same analytical framework is then applied to evaluate the demodulation for all other selected W configurations (Section 5.5.5, Section 5.5.6, Section 5.5.7, Section 5.5.8, Section 5.5.9, Section 5.5.10, Section 5.5.11 and Section 5.5.12). Similarly to the SOTA HSPy-SI demodulation evaluation, a detailed analysis will be presented for all four selected bands (i.e., 714 nm, 730 nm, 742 nm and 755 nm) if any particular configuration satisfies the evaluation criteria in at least one (i.e., 742 nm) of these bands. The E-39-W-0-S-30 configuration (i.e., no-polarizer setting) was chosen to assess demodulation as the initial evaluation case. The details can be found in Appendix C.3.3.

#### 5.5.5. Evaluation of E-39-W-0-S-30

**Figure 14:**
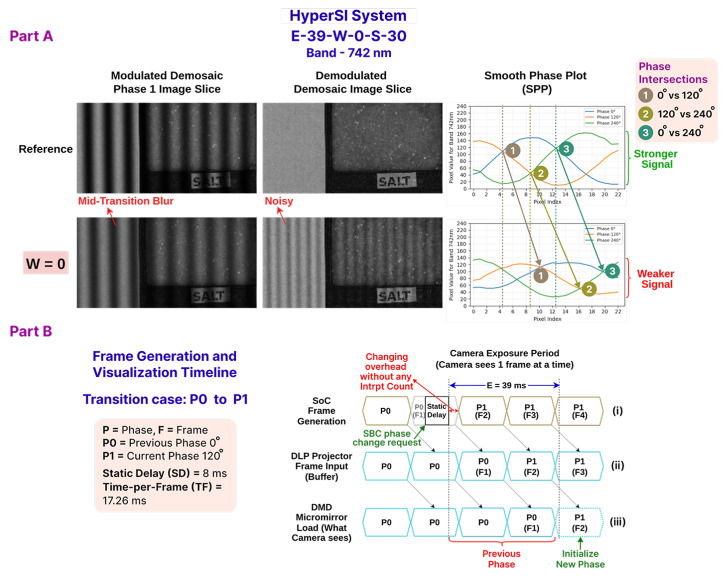
In Part (**A**), The first phase transition from phase P0 to P1 is illustrated through captured modulated images and their corresponding demodulated results compared to their references for the E-39-W-0-S-30 configuration. The SPP curves indicate that the signal is stronger or weaker. In Part (**B**), row (**i**) highlights the SoC frame-generation timeline. Row (**iii**) illustrates what the camera recorded: the tail end of P0, a complete P0, and P0(F1). Since nearly the entire camera exposure period was filled with incorrect phases, the modulated image in Part (**A**) exhibits mid-transition blur (marked in red) close to P0, while the SPP markers (1–3) remain misaligned relative to the reference. Consequently, the demodulated image appears noisy. This configuration fails to meet both acceptance criteria from Section 5.5.1 and is rejected.

Figure 14 presents the first phase transition (i.e., P0 to P1), and Part A illustrates the slices taken from the top-left corner of the demosaiced modulated and demodulated images of both the reference and final captures of the 742 nm band. Part B illustrates three frame-sequence timelines from generation to visualization for both the SoC and the DLP projector.

The SBC made a GPIO request to change the phase from P0 to P1, which resulted in the call to the ISR Algorithm 1. For W = 0, the timelines for SoC frame generation, projector frame input, and DMD projection are almost similar to the process of W = 1 (Section 5.5.4). In contrast to W = 1, the interrupt counts for applying a delay to the camera were not measured for W = 0. Consequently, the residual portion of P0(F1) (i.e., the changing overhead without an interrupt count) in row (i) shifts to the right relative to the start of the camera exposure window. As a result, a uniform rightward shift of the entire frame sequence was observed in all timeline rows (i), (ii), and (iii), corresponding to the residual portion of P0(F1). Since the interrupt count was not measured, the recorded CPT was only 8 ms for the static delay (i.e., to stabilize the GPIO communication), as depicted in Figure 13a. The time required to complete the residual portion of P0(F1) is equivalent to the conceptualized first-interrupt arrival time period. Section 5.5.3 reported measurements of the first-interrupt arrival times, and they are plotted in Figure 13b for W = 1, 2, and 3. Therefore, it can be assumed that the residual completion time of P0(F1) could range from 4.08 to 10.6 ms, since it was not measured. The remaining steps are the same as those for W = 1, already explained in Section 5.5.4, in terms of the timeline for frame generation, projector buffer management (i.e., projector frame input), and projection. Up to this point, the behavior described previously with respect to the SoC and DLP projector remains the same for W = 0, regardless of exposure time settings.

The camera sensor accumulated and recorded the incoming reflected light from the sample as information, which was later converted to a pixel value by the drivers. During most of the exposure window, the sensor recorded information from the incorrect phase, i.e., the tail end of P0, the complete P0, and P0(F1). For the two complete incorrect frames, ≈89% of the data (i.e., $(\frac{\left(17.26\times 2\right)}{39})\times 100$) already accumulated by the sensor were incorrect. Furthermore, since the residual of P0(F1) (i.e., in the SoC frame-generation timeline row (i)) was not measured and could range between 4.08 and 10.6 ms, it can be concluded with certain confidence that more than 89% of the phase data captured by the camera were incorrect. Hence, the correct phase P1(F2) was just initialized at the tail end of the exposure window, and the camera could barely record this frame.

However, the modulated image exhibits a minor yet perceptible change, remaining largely consistent with the previous phase P0 while appearing somewhat blurred. DLP renders each frame time-weighted bit-plane segments (i.e., MSB and LSB planes), where higher-weight segments (i.e., MSB) add to the brightness. For further explanation, see Appendix B.1: Table A4 for 58 Hz and Table A1 for the allocation of 8-bit bit-plane time at 60 Hz. During the correct phase P1(F2), the reorientation of the micromirrors had only just begun within the exposure period of the camera. As a result, only a small portion of the intended P1(F2) frame contributed to the captured light, while most of the light originated from the frames of the previous phase (i.e., partial P0, P0, and P0(F1)). As a result, this partial mixing caused a slight blur; however, the modulated image still mostly resembled the previous phase, P0. These phenomena can be further validated by the SPPs illustrated in Part A. The phase-intersection markers 1, 2, and 3 for the captured image exhibit a substantial uniform phase shift compared to the reference SPP, and the signal strength is also weaker. The modulated image closely resembles P0 rather than the correct P1 and appears blurry. Consequently, the demodulated image was full of artifacts. Since the findings for E-39-W-0-S-30 violated both acceptance criteria for demodulation, this configuration was rejected.

#### 5.5.6. Evaluation of E-39-W-1-S-30

**Figure 15:**
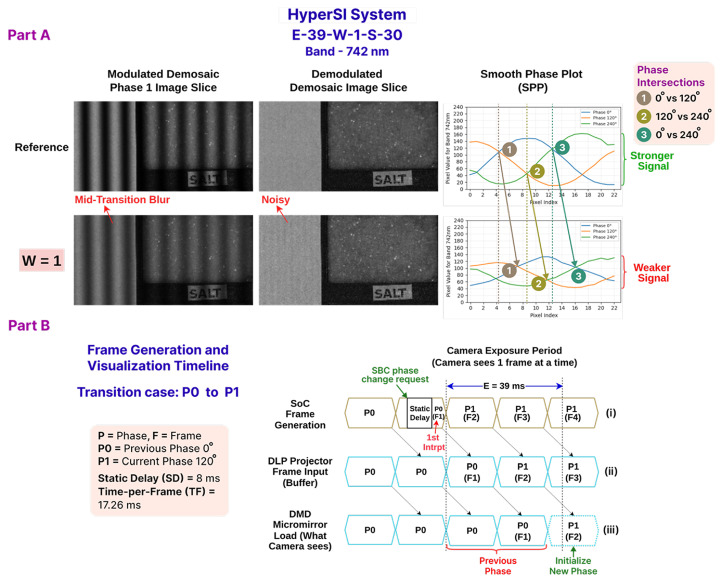
In Part (**A**), The transition from phase P0 to P1 is illustrated through captured modulated images and their corresponding demodulated results compared to their references for the E-39-W-1-S-30 configuration. The SPP curves indicate that the signal is stronger or weaker. In Part (**B**), row (**iii**) illustrates what the camera sequentially recorded: two frames from the previous phase (P0 and P0(F1)) followed by the partially correct-phase initialization of P1(F2) (marked as a dotted frame). Since more than half of the captured phase data comes from the previous phase, the modulated image in Part (**A**) shows mid-transition blur (marked in red). The SPP markers (1–3) also appear misaligned with respect to the reference. As a result, the demodulated image looks noisy. This configuration does not meet the acceptance criteria in Section 5.5.1 and is therefore rejected.

Figure 15 illustrates the captures taken with one frame interrupt for the projection–capture synchronization delay. In contrast to W = 0, when the SoC board received the GPIO request to change the phase to P1, the ISR Algorithm 1 started counting the ap_done interrupt from the pattern-generator IP to introduce the camera delay. An explanation of the process for W = 1 can be found in Section 5.5.4. Section 5.5.3 already established that this first interrupt always originates from the previous phase (i.e., from P0 for the transition from P0 to P1) and was measured as 5.1 ms (Figure 13b). Up to this stage, the behavior explained earlier with respect to the SoC and DLP projector remains the same for W = 1 regardless of exposure time settings.

The HS camera recorded sequential phases: incorrect P0 and P0(F1) and partially correct-phase initialization P1(F2). Similarly to the previous E-39-W-0-S-30 configuration, the camera still accumulated ≈89% of incorrect-phase information. However, in contrast to the previous configuration, since the configurable camera delay was implemented during the first-interrupt count period, the residuals of P0(F1) (i.e., with respect to the SoC frame-generation timeline) were absent within the exposure window. As a result, the entire frame sequence uniformly shifted to the left by the value of the first-interrupt arrival time period for P0(F1) in all given timelines. Thus, 39 − (2 × 17.26) = 4.48 ms time was left for P1(F2). During the final 4.48 ms, the controller began to reorient the micromirrors across the red, green, and blue channels by interleaving and intertwining the bit-plane segments (MSB and LSB) (Section 4.1). From the captured modulated image, it is apparent that a significant change occurred. This change is also visible in the SPP markers, which moved closer to the reference SPP compared to the previous capture experiment E-39-W-0-S-30. This indicates that the modulated image approached the correct phase P1 but still appeared blurred. Since the MSB segments contribute the highest intensity (i.e., brightness), they evidently account for the observed noticeable change.

In contrast to the previous configuration, it was evident that the acquired modulated image was gradually approaching the desired P1 phase. SPP markers 1, 2 and 3 were also found to move closer to the reference compared to the markers of the E-39-W-0-S-30 configuration. Despite the small improvement in the SPP markers and the modulated image, the phase transition remained visible in both the modulated image and the SPP compared to the reference. Consequently, a small number of artifacts were found in the demodulated image slice. Thus, the E-39-W-1-S-30 configuration fails to meet both acceptance criteria and must be rejected.

#### 5.5.7. Evaluation of E-39-W-2-S-30

**Figure 16:**
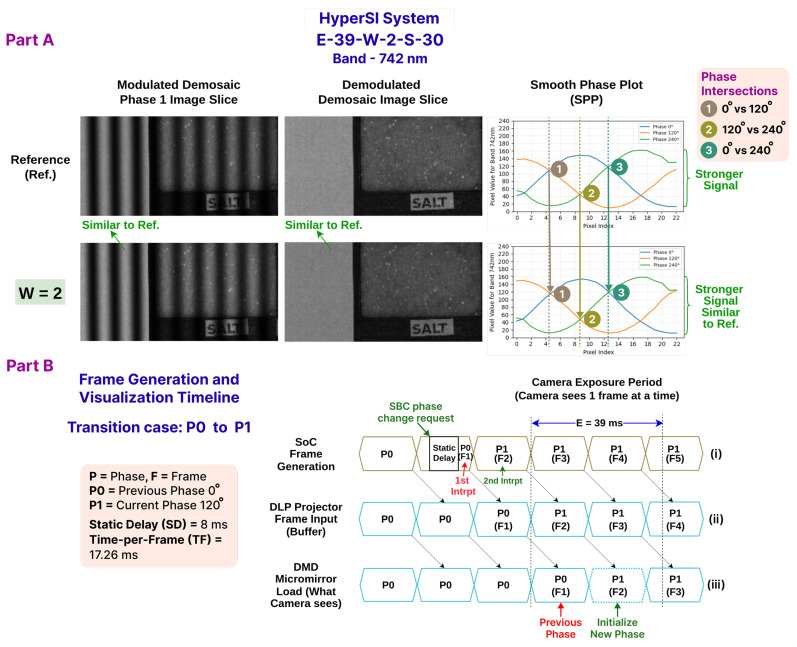
For the E-39-W-2-S-30 configuration, in Part (**A**), The transition from phase P0 to P1 is illustrated through captured modulated images and their corresponding demodulated results compared to their references. The SPP curves indicate that the signal is stronger or weaker. In Part (**B**) row (**iii**) shows what the camera sequentially recorded: the previous phase P0(F1), followed by the correct-phase initialization P1(F2) (dotted frame) and a partial P1(F3). Since most of the captured data belongs to the correct phase, the modulated image in Part (**A**) closely matches the reference (marked green). The SPP markers (1–3) also align well with the reference, with strong signal intensity. As a result, the demodulated image quality improves, and this configuration meets the acceptance criteria and is accepted.

Figure 16 illustrates the captures taken with two frame interrupts for the projection–capture synchronization delay. Compared to W = 1, the entire frame sequence was uniformly shifted to a position to the left of the exposure window. After receiving the GPIO phase-change request, the SoC counts the first interrupt of the previous phase, P0(F1), measured as 5.14 ms (Figure 13b), and then counts the second interrupt for the correct phase P1(F2). Thus, P1(F2) was initialized and stabilized in the DMD micromirror timeline row (iii) (i.e., frame visualization timeline). Up to this point, the previously described behavior of the SoC and the DLP projector remains the same for all W = 2, regardless of the exposure time settings.

The HS camera recorded three sequential image frames: the incorrect phase P0(F1), the correct phase P1(F2), and the final P1(F3) that was partially visible within the exposure window. Compared to the previous E-39-W-1-S-30 configuration’s 89% of incorrect-phase acquisition, the camera accumulated $(\frac{(17.26\times 1}{39})\times 100\approx 44\%$ of incorrect information: less than half the incorrect-phase data. In the remaining 56% (i.e., 39 − 17.26 = 21.74 ms) of the exposure window, the micromirrors stabilized for the first correct phase P1(F2) within the first 17.26 ms, and P1(F3) was visible in the remaining 21.74 − 17.26 = 4.48 ms.

However, this yielded a dramatic improvement: the captured modulated image closely matched the reference, and all SPP markers aligned with the reference guides, also showing strong signal intensity. This happened because the camera integrated over time, and the correct pattern occupied the majority (including the high-weight MSB plane portions for partially visible P1(F3)) of that time; the 56% “good” window was enough to dominate the average intensity, thus producing a clean modulated image. In addition, this observation establishes that the increment in W enhances demodulation quality. The findings for E-39-W-2-S-30 conform to both demodulation acceptance criteria; this configuration was accepted.

**Figure A17:**
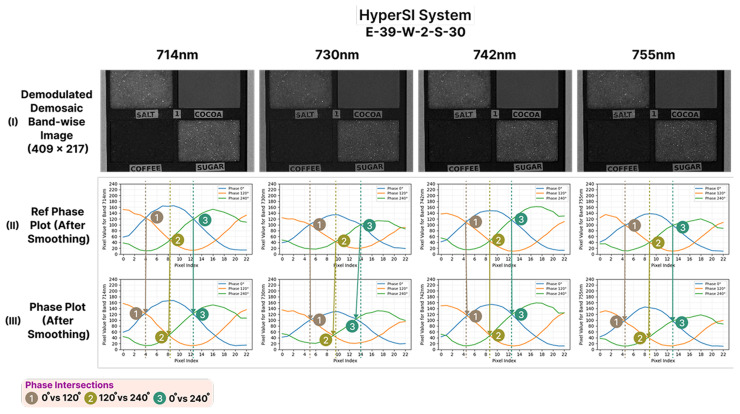
The figure illustrates the demodulated capture by the HyperSI system for the E-39-W-2-S-30 configuration, with its SPP phase-shift comparison against the reference. The four spectral bands (714 nm, 730 nm, 742 nm, and 755 nm) are arranged in columns.

Since this configuration met the acceptance criteria, a further analysis was performed on the remaining three chosen spectral bands, 714 nm, 730 nm and 755 nm, together with 742 nm. All bands satisfied the demodulation criteria (Section 5.5.1) and were considered acceptable. A detailed description of the analysis is further explained in Appendix C.3.4, Figure A17.

#### 5.5.8. Evaluation of E-39-W-3-S-30

The E-39-W-3-S-30 configuration meets both demodulation acceptance criteria and is accepted. The details of the analysis are further explained in Appendix C.3.5.

#### 5.5.9. Evaluation of E-213-W-0-S-30

**Figure 17:**
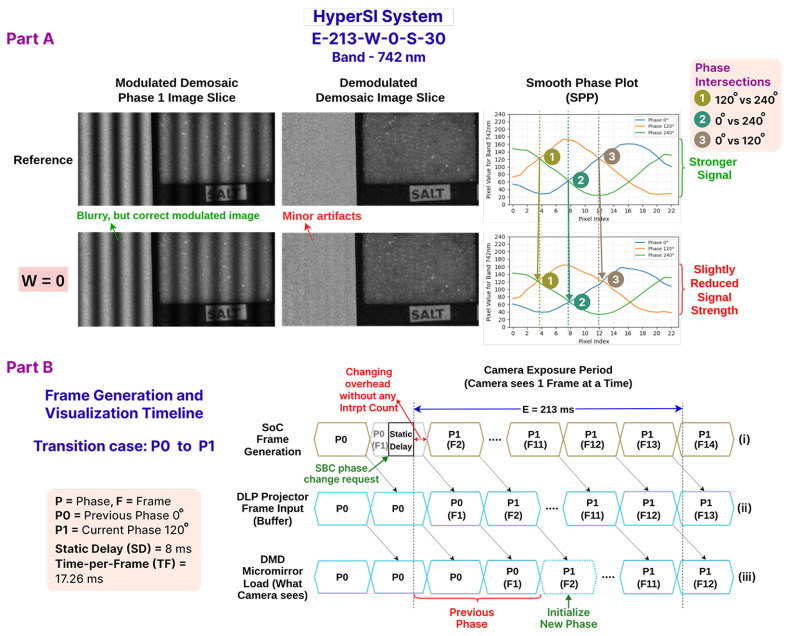
For the E-213-W-0-S-30 configuration, in Part (**A**), transition from phase P0 to P1 is illustrated through captured modulated images and their corresponding demodulated results compared to their references. The SPP curves indicate that the signal is stronger or weaker. Part (**B**) row (**iii**) shows that the camera sequentially recorded ≈13 frames: the tail end of P0; two full incorrect frames, P0 and P0(F1); the initialization of P1(F2) (marked as a dotted frame); ten correct stable frames, P1(F3) to P1(F11); and the start of P1(F12). Since the polarizer reduces the light reaching the camera sensor, the captures became energy-sensitive. The modulated image looks slightly blurred, the SPP is aligned with the reference but has a weaker signal strength, and the demodulated image contains noise (marked red). This configuration does not meet Criterion 2 and is therefore rejected.

Figure 17 shows the phase transition (i.e., P0 to P1) for an exposure of 213 ms with the polarizer setting. Since W = 0, no interrupts were measured; this configuration also mimicked the SoC frame generation and the DLP projector visualization sequence (i.e., DMD micromirror load timeline) described in Section 5.5.5, with the only difference being the exposure window period.

The HS camera accumulated the information of ≈13 sequential image frames of mixed phases (i.e., correct and incorrect), one by one: the tail end of the first incorrect phase, P0; two complete incorrect phases, P0 and P0(F1); the new correct-phase initialization, P1(F2); ten consecutive correct phases, P1(F3) to P1(F11); and finally the commencement of P1(F12). The last correct phase, P1(F12), was barely inside the exposure window. Although the SPP markers were aligned, a subtle blurriness was seen in the modulated image compared to the reference image. Consequently, a slight visible reduction in signal strength was found in the SPP compared to the reference image, and minor artifacts were also observed in the demodulated image.

During the 213 ms exposure, only about two and a half frames (out of ≈13 frames) contained incorrect-phase data. The no-polarizer configuration for the optimal W count (i.e., E-39-W-2-S-30) produced a correct modulated image and a clean demodulated output, even with only about 56% correct-phase data. In contrast, the polarizer configuration, despite having approximately 81% correct-phase data (i.e., $(\frac{\left(17.26\times 10\right)}{213})\times 100\approx 81\%$), yielded a modulated image very close to the correct phase, but with slight blurriness and minor artifacts in the demodulated result. The blur and resulting artifacts occurred because the polarizer blocked direct reflections and allowed only diffused light to pass, greatly reducing the energy reaching the camera sensor. This made the phase data captured by the camera sensor highly sensitive to variations in incoming light. As confirmed by the white calibration experiment (Section 5.1.2), the sensor required more than five times longer exposure (i.e., 213 ms vs. 39 ms) for the polarizer setting to achieve the same brightness level. In addition, Section 5.4.2 already explained that lower energy levels (i.e., ≤50%) received by the sensor can cause consistent noise, regardless of the synchronizer system or exposure time. This further supports the explanation for the blurriness of the modulated image and the resulting artifacts. Therefore, the information captured by the sensor during the exposure window became highly sensitive to the amount of incoming energy. In addition, Section 5.4.2 has already explained how less energy (i.e., ≤50%) received by the sensor can create consistent noise, regardless of the synchronizer system and exposure time. This further confirms the reason behind the blurriness of the modulated image and the resulting artifacts. Therefore, the information captured by the sensor throughout the exposure window became very sensitive to the amount of incoming energy. However, this configuration violated Criterion 2 and was therefore rejected.

#### 5.5.10. Evaluation of E-213-W-1-S-30

**Figure 18:**
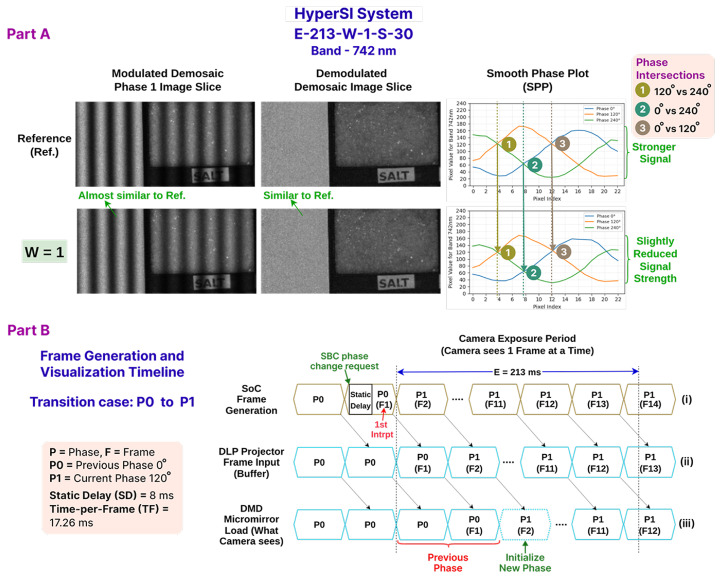
For the E-213-W-1-S-30 configuration, in Part (**A**), transition from phase P0 to P1 is demonstrated through captured modulated images and their corresponding demodulated results compared to their references. The SPP curves indicate that the signal is stronger or weaker. Part (**B**) row (**iii**) shows that the camera sees about 13 frames: incorrect phases P0 and P0(F1), the correct-phase initialization P1(F2), stable phases P1(F3)–P1(F11), and a partial P1(F12). The modulated image is nearly identical to the reference. The SPP markers are aligned with the reference but show slightly reduced signal strength. The demodulated image appears clean. This configuration meets the acceptance criteria; therefore, it is accepted.

Figure 18 illustrates the captures taken with one frame interrupt for the projection–capture synchronization delay. For W = 1, the first interrupt was recorded at 10.6 ms (Figure 13b). This configuration also reuses the SoC frame generation and DLP visualization timeline (i.e., DMD micromirror load timeline) sequence described in Section 5.5.6, with the difference being the camera exposure window.

In contrast to the previous configuration E-213-W-0-S-30, this one does not have a partial incorrect phase P0 visible at the beginning of the exposure window due to the interrupt count. Hence, the HS camera recorded ≈13 sequential image frames: incorrect phases P0 and P0(F1), correct-phase initialization and stabilization P1(F2), P1(F3) to P1(F11), and partially visible P1(F12) in the exposure window. The SPP markers are aligned with a mild reduction in signal strength. The modulated image was almost similar to the reference, with a clean demodulated image.

In the previous configuration, it was found that the accumulated light information captured by the camera sensor was sensitive to polarizers. Compared to the previous configuration, removal of only the tail end of P0 from the beginning of the exposure window was enough to cause this significant improvement for the E-213-W-1-S-30 configuration. These findings further confirmed that the polarizer made the captured image more sensitive to incoming light. SPP markers 1, 2 and 3 align with the reference guides, confirming that the phase timing itself is correct, as before. Compared to the reference, the modulated image exhibits a subtle, nearly imperceptible blurriness. Consequently, its SPP shows a slight reduction in signal strength. However, according to the demodulation evaluation criteria defined in Section 5.5.1, the findings for E-213-W-1-S-30 conform to both acceptance criteria, so this configuration was accepted. In addition, since this study proposes a tunable synchronizer with respect to W as the core contribution, users have the freedom to decide which tuning point they may or may not choose.

**Figure A20:**
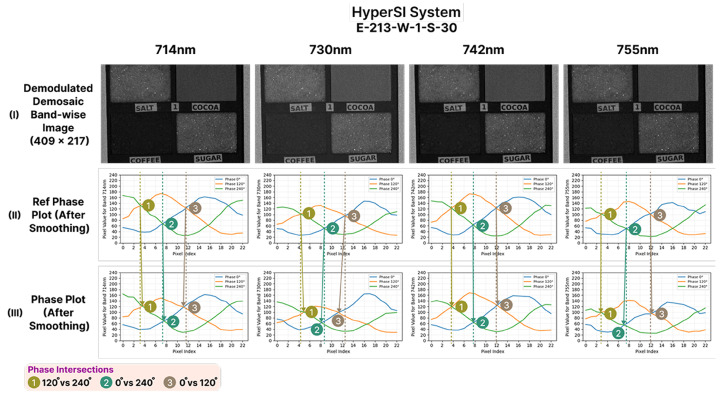
The figure presents the demodulated capture by the HyperSI system for the E-213-W-1-S-30 configuration, with its SPP phase-shift comparison against the reference. The four spectral bands (714 nm, 730 nm, 742 nm, and 755 nm) are arranged in columns.

As this configuration satisfied the acceptance criteria, additional analysis was performed for the remaining three chosen spectral bands at 714 nm, 730 nm, and 755 nm, along with 742 nm. All bands satisfied the demodulation criteria (Section 5.5.1) and were considered acceptable. A detailed description of the analysis is further provided in Appendix C.3.6, Figure A20.

#### 5.5.11. Evaluation of E-213-W-2-S-30

The E-213-W-2-S-30 configuration meets both demodulation acceptance criteria and is accepted. The details of the analysis are further explained in Appendix C.3.7.

#### 5.5.12. Evaluation of E-213-W-3-S-30

The E-213-W-3-S-30 configuration meets both demodulation acceptance criteria and is accepted. The details of the analysis are further explained in Appendix C.3.8.

#### 5.5.13. Determination of the Required Minimum W

The determination of the minimum required *W* is necessary to guarantee proper phase alignment between SoC-generated frames and the output of the DLP projector. It ensures that the projection begins with the correct-phase frame and maintains stable micromirror operation without temporal or phase misalignment. From the experimental observations in Section 5.5.5, Section 5.5.6, Section 5.5.7, Section 5.5.8, Section 5.5.9, Section 5.5.10, Section 5.5.11 and Section 5.5.12, the synchronization behavior with respect to different experimental values of *W* was explored to satisfy the demodulation criteria. In addition to this, a distinct behavioral pattern of the synchronizer was also observed in terms of the projected frame corresponding to the correct phase and the stabilization of the DLP micromirrors. It was found that for W=1, the first two projected frames (i.e., the DMD micromirror load) originated from the previous phase; for W=2, the first projected frame originated from the previous phase; and for W=3, all frames corresponded to the correct phase. This pattern indicates that both the SoC and the DLP projector introduce timing offsets that must be compensated for by selecting an appropriate *W*.

The DLP projector contributes to a one-frame latency (i.e., L=1) due to its dual-buffer architecture. Additionally, micromirrors require approximately one frame duration (i.e., s=1) to reorient and stabilize before displaying a fully correct-phase frame. Furthermore, it was consistently observed that the first interrupt $I_{1st}$ originates from the frame of the previous phase, introducing an additional one-frame offset that must be taken into account.

To represent these combined effects, the number of incorrect or unstable frames at the beginning of the projection can be expressed using Equation (12):

(12) $$E\left(W\right)=\max\left(0,\;(L+C_{phase}+\chi_{stab}\times s)-W\right)\;\;\;\left[\mathrm{where};C_{phase}=2\right]$$

where E(W) is the number of incorrect or unstable frames projected through DMD micromirrors. When E(W)=0, all projected frames are in the correct phase. The $C_{phase}$ is the phase compensation constant that compensates for two inherent frame-level delay counts: the first arises because the initial interrupt $I_{1st}$ originates from the frame of the previous phase, and the second corresponds to the additional frame required to reach the first correct-phase boundary. Hence, the constant value of $C_{phase}$ is defined as $C_{phase}=2$. $\chi_{stab}\in \left\{0,1\right\}$ is an indicator variable, where 0 denotes that stabilization is not required, and 1 denotes that stabilization is required.

From Equation (12), the minimum required *W* to achieve the correct phase and, when enabled, stabilized projection is presented in Equation (13).

(13) $$W_{\min}=L+C_{phase}+\chi_{stab}\times s$$

Therefore, for the present system, where L=1 and s=1, the above relation yields the results shown in Equations (14a) and (14b).

(14a) $$\begin{array}{cccc} W_{\min} & =3 & \; & [\;\chi_{stab}=0;\;\mathrm{phase-correct}\;\mathrm{projection}]\;\;\;\;\;\;\;\;\;\;\;\;\;\;\;\;\;\;\;\;\;\;\;\;\;\;\;\;\; \end{array}$$

(14b) $$\begin{array}{cccc} W_{\min} & =4 & \; & \left[\;\chi_{stab}=1;\;\mathrm{phase-correct}\;\mathrm{and}\;\mathrm{stabilized}\;\mathrm{projection}\right] \end{array}$$

Thus, W=3 ensures that all frames belong to the correct phase, while W=4 guarantees both phase correctness and micromirror stabilization. This unified expression captures the combined effects of DLP latency, micromirror stabilization, and interrupt–phase offset, providing a clear criterion for selecting an appropriate *W* to achieve accurate frame synchronization between the SoC and the DLP projector.

#### 5.5.14. Performance Evaluation of SOTA HSPy-SI vs. HyperSI

**Figure 19:**
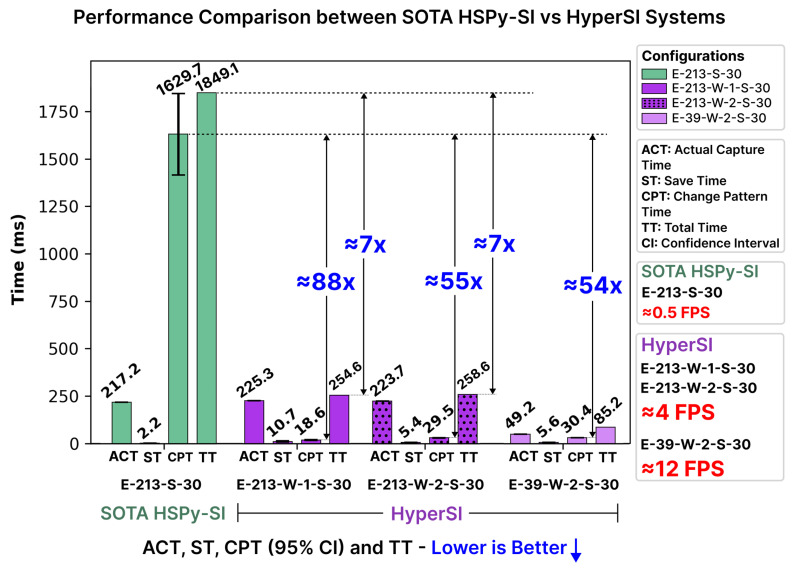
Runtime performance comparison between SOTA HSPy-SI and HyperSI systems.

Three HyperSI configurations with optimal W values were selected based on the evaluation of the quality of the demodulation. These configurations were then used to compare runtime performance with the SOTA HSPy-SI system. The configurations E-213-W-1-S-30 and E-213-W-2-S-30 satisfied the criteria under the polarizer setting. The configuration E-39-W-2-S-30 met the criteria under the no-polarizer setting. Figure 19 illustrates the performance comparison between the SOTA HSPy-SI and HyperSI systems for all selected configurations. These three configurations offered the best trade-off between synchronization accuracy and performance. The comparison was based on the mean values of three measured metrics for 95% CI: ACT, ST, and CPT. These metrics were selected because they serve as key performance indicators for the core capture loop to capture modulated HS images for the proposed system (for a further explanation, see Appendix B.5.2). Each of the metrics was averaged over 30 modulated samples. TT was calculated as the sum of the means of ACT, ST, and CPT.

**ACT:** ACT encompasses not only camera exposure but also sensor readout, USB3 transfer overhead, driver and CPU handling, and any on-board image preprocessing. After each capture, the image frame is split into smaller packets, errors are corrected (camera-model-dependent), and the packets are transferred over the USB bus to memory [53]. Consequently, this process adds a few milliseconds of delay. In the SOTA desktop system, USB3 runs over a dedicated multilane PCIe link with efficient DMA and low interrupt latency, minimizing transport delays; hence its ACT is 217.2 ms. In contrast, the proposed system uses an RPI 4 model (i.e., SBC), where USB3 shares a single PCIe lane with Ethernet and NVMe devices. This shared configuration introduces queueing and scheduling delays. It also adds more overhead from the driver and CPU. As a result, the ACT increased slightly. For the E-213-W-1-S-30 and E-213-W-2-S-30 configurations, the ACTs were 225.3 ms and 223.7 ms, respectively. Similarly, for the E-39-W-2-S-30 configuration, a higher ACT of 49.2 ms was observed for the proposed system.

**CPT:** A large improvement in CPT was observed for the proposed system versus the SOTA. CPT measures the delay required for the camera to capture stable frames of the correct phases. The proposed system achieves granular frame control by counting HW interrupts arriving after each frame generation, bypassing any OS-layer delays, whereas the SOTA HSPy-SI system incurs hidden processing overhead, including the static delay (i.e., (exposure_time + 1 s) + (exposure_time + 1 s) + 1.25 s) defined to achieve synchronization (further explained in Appendix A.3). Taking into account the polarizer setting (i.e., E-213), the CPT of the SOTA system was 1629.7 ms. In comparison, the proposed system achieved 18.6 ms with W = 1 and 29.5 ms with W = 2. These represent improvements of approximately 88× and 55×, respectively. The SOTA HSPy-SI system was designed to support only the polarizer setting (i.e., a longer exposure time). Even if it is assumed to work with a shorter exposure time (i.e., E-39), it will still rely on a fixed delay for synchronization. In comparison, the proposed system for such a case achieves approximately 54× faster performance for W = 2.

**ST:** ST is how much time is needed to save the image to storage. The SOTA system saves the captured images to a high-speed internal NVMe M.2 SSD with a write speed of approximately 3000 MB/s. As a result, it achieves a storage time (ST) of just 2.2 ms for the E-213-S-30 configuration. In contrast, the HyperSI system stores images on a microSD card, which has a higher write latency. This results in a longer storage time (ST): 10.7 ms for E-213-W-1-S-30, 5.4 ms for E-213-W-2-S-30, and 5.6 ms for E-39-W-2-S-30.

**TT:** Since TT = ACT + CPT + ST, the CPT measure had a significant influence on TT. For the polarizer setting (E-213-S-30), CPT was measured as 1849.1 for the SOTA. Meanwhile, the CPTs for the proposed system were only 254.6 ms (E-213-W-1-S-30) and 258.6 ms (E-213-W-2-S-30), both improved by ≈7×. Although the SOTA HSPy-SI system captured approximately one modulated frame every two seconds, the proposed system achieved a modulated capture rate of around 4 FPS. The most significant improvement in FPS was observed in the no-polarizer setting of the proposed system, where the modulated capture rate was nearly 12 FPS.

**Figure 20:**
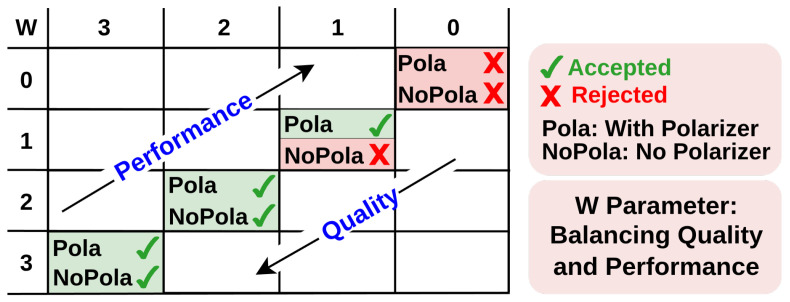
A decision matrix showing the trade-off between runtime performance and demodulation quality for both polarizer and no-polarizer settings, influenced by the W parameter. Smaller W values improve performance but may slightly reduce demodulation quality. In contrast, increasing W enhances quality at the cost of runtime performance. The variation across configurations is moderate but noticeable.

Figure 20 illustrates the decision matrix for selecting the optimal W values based on performance and quality. In particular, W = 1, 2, 3 was accepted for the system with a polarizer (i.e., 213 ms), and W = 2, 3 for the system without a polarizer (i.e., 39 ms). These selections were made according to the acceptance criteria for demodulation defined in Section 5.5.1. The choice of W depends on user priorities: selecting a lower W maximizes performance, while a higher W improves demodulation quality.

**Figure 21:**
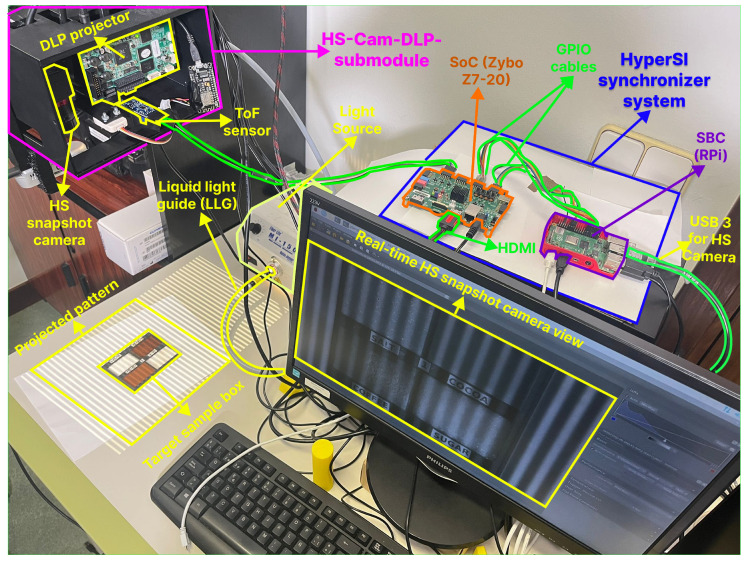
A photograph of the assembled HyperSI bench-top prototype used in this study. The system integrates an RPI 4 Model B as the master controller and a Zybo Z7-20 SoC board as the slave controller for deterministic pattern generation and projection–capture synchronization. The SoC is interfaced with the DLP projector through HDMI, while the SBC is connected to the HS snapshot camera through USB. GPIO/PMOD-based signaling is used between the SBC and SoC to coordinate pattern switching and image capture timing. The figure highlights the implemented hardware architecture and the compact embedded form factor of the proposed synchronizer prototype.

Figure 21 shows the assembled HyperSI bench-top prototype used in this study as a real-time projection–capture synchronizer proof-of-concept. The prototype integrates an RPI 4 Model B as the master controller (i.e., SBC) and the Zybo Z7-20 SoC board (i.e., SOC) as the slave controller for deterministic pattern generation and synchronization control. The SoC is interfaced with the DLP projector through HDMI for structured pattern projection, while the SBC is connected to the HS snapshot camera through USB3 for image acquisition. Communication between the SBC and SoC is established through GPIO/PMOD-based signaling to coordinate pattern switching and capture timing. This figure provides direct evidence of the implemented hardware architecture and highlights the compact embedded form factor of the proposed HyperSI synchronizer prototype.

## 6. Discussion

This research begins with studying the SOTA HSPy-SI projection–capture synchronizer system. It then proposes a successful design and development methodology for a high-performance, configurable, real-time SI synchronizer system, named the HyperSI system. Both systems follow the conventional TPD method to implement the SI principle and use the same UVC-compliant HS snapshot camera and DLP projector model that supports HDMI video mode. The chosen DLP projector introduces a one-frame projection (i.e., display) delay due to its dual-buffer mechanism. Hence, both systems must compensate for this delay to achieve accurate projection–capture synchronization. The SOTA system handles all core tasks, including frame generation, pattern phase transitions, HDMI streaming to the DLP projector, and camera capture. In contrast, the proposed HyperSI system distributes these tasks across two dedicated computing platforms: an SBC to control the change of pattern and capture image, and an SoC for high-performance pattern generation. Thus, the SBC acts as the master controller, managing the slave SoC-based system through GPIO communication. Both systems use a DMD-based DLP projector to project three-phase patterns, with HDMI input and video mode as required specifications.

Two camera exposure settings, one with a polarizer and one without (i.e., no-polarizer setting), are used to capture modulated images across three phases. The SOTA HSPy-SI system can capture modulated images only when using polarizers, which require a longer exposure time (i.e., 213 ms). This limitation arises because the system relies on a desktop computer for both frame generation and synchronization, lacking the low-level hardware access needed for accurate frame-level control. As a result, to address the one-frame projection latency caused by the DLP projector, the synchronizer delay was determined only for the polarizer configuration (i.e., the longer, 213 ms exposure) using a trial-and-error approach within a large search space. In contrast, the proposed HyperSI system offers greater flexibility in capture configurations by supporting both polarizer and no-polarizer settings (i.e., shorter 39 ms of exposure). This is because it uses a dedicated SoC-based frame generator with direct low-level HW access. This design allows for precise control over each 24-bit RGB pattern frame generation at approximately 58 Hz, about 8× faster than the SOTA HSPy-SI system. The pattern quality is validated by achieving zero RMSE compared to reference patterns generated using 64-bit floating-point precision on an Intel processor. To accurately address frame-level projection latency (i.e., one-frame display delay), the proposed system introduces a configurable W parameter implemented in the SoC as a projection–capture synchronization tuner, configurable from the SBC controller. The W parameter sets how long (i.e., in terms of the number of generated pattern frames) the SBC waits before triggering the camera capture. During this time, the SoC counts the corresponding number of frame-generation interrupts. Hence, it was necessary to verify that the interrupt period *I* matches the measured frame period TF (i.e., I≈TF). This was experimentally confirmed, where the measured interrupt period of 17.255 ms closely matched the oscilloscope measurement of 17.26 ms. A small deviation was observed for the first interrupt, which originated from a frame of the previous phase but remained within a predictable range of 4.08 to 5.18 ms (except for one outlier measured as 10.6 ms) during the capture experiment.

This study presents an end-to-end methodology in which the SoC-based system was developed through a structured, reproducible, step-by-step HW/SW co-design process. The method for determining the core HW clock (i.e., 86 MHz) and configuring the VTC parameters adheres to the VESA standard. This ensures adaptability at a resolution of 912×1140 and an FRR of approximately 58 Hz (targeting 60 Hz), providing compatibility with VESA-compliant display devices for HDMI I/O. In particular, the HDMI interface support makes this system projector-model-agnostic. A comprehensive methodology has been established to design the parameterized pattern-generator IP using a dataflow MoC implemented through HLS. This approach enables real-time, high-performance pattern streaming with 1 PPC. The HW design illustrates how key IP cores, including the pattern generator, VTC, and AXI GPIO modules, are integrated to support the functional requirements of the embedded SoC controller. Furthermore, it outlines the ISR algorithm, which is triggered by the SBC via GPIO, to change the pattern and implement a configurable frame-generation-based interrupt delay according to the specified W count. Meanwhile, a simple SBC algorithm was developed to serve as the master controller to manage the SoC system. This manages camera capture and controls pattern phase changes on the SoC via GPIO communication. Since the UVC-compliant HS snapshot camera is connected via a USB interface to the SBC, the system remains compatible with any snapshot camera that supports the UVC standard.

Evaluation is an integral part of the proposed methodology, for which specific criteria have been defined to evaluate the demodulated images produced by both the SOTA HSPy-SI and HyperSI systems. The modulated images captured by both systems, across all configurations, were compared with the reference captures. The evaluation was conducted on the basis of the quality of the resulting demodulated images and their corresponding phase plots. Modulated image captures were performed using a polarizer with the SOTA HSPy-SI synchronizer. Unlike the SOTA system, the captures performed by the proposed HyperSI system are directly influenced by the parameter W. Hence, multiple capture configurations of the proposed system were explored by varying the tunable parameter W in the range of 0 to 3. The phase transition from P0 to P1 was selected as the candidate modulated capture for the evaluation of both systems. Based on the evaluation criteria, three optimal configurations (i.e., with minimum acceptable W values) were identified for the proposed system: E-213-W-1-S-30 and E-213-W-2-S-30 for the polarizer setting, and E-39-W-2-S-30 for the no-polarizer setting. Under the polarizer setting, the SOTA system recorded a CPT of 1629.7 ms. In contrast, the proposed system achieved significantly faster performance, with CPTs of 18.6 ms for W = 1 and 29.5 ms for W = 2. These correspond to performance improvements of roughly 88× and 55×, respectively. Although the SOTA HSPy-SI system captured roughly one modulated frame every two seconds, the proposed system reached a capture rate of approximately 4 FPS. The greatest improvement in FPS was observed in the no-polarizer setting in the proposed system, where it was close to 12.

The demodulation analysis in this study was based on two explicitly defined acceptance criteria: first, that at least two of the pre-selected bands exhibit phase shifts closely aligned with the reference vertical guides of the reference SPP, and second, that the resulting demodulated images remain free of visible noise or artifacts. In parallel, a deterministic synchronization analysis of the proposed system indicated that the conservative safe operating range for the delay parameter W is approximately 3 to 4. These two analyses serve different purposes: the deterministic W range defines a safer synchronization margin from a system-timing perspective, whereas the demodulation criteria evaluate whether the captured data are practically sufficient to produce acceptable image quality. Therefore, the fact that some lower W values also produced acceptable demodulation does not imply that the criteria were weak; rather, it shows that the system can still tolerate certain non-ideal synchronization conditions while maintaining acceptable output quality. Hence, the lower acceptable values of W do not indicate that the evaluation criteria were too weak. Rather, they show that there is a difference between the minimum deterministic safe synchronization value and the minimum experimentally acceptable value for demodulation. The calculated W = 3 to 4 range represents a conservative synchronization setting derived from the projector–camera timing model so that the camera capture is more safely aligned with the intended projected phase. In contrast, the experimental results show that acceptable demodulation can still occur at lower W values when the captured signal contains a sufficient proportion of the correct-phase information and the demodulated image remains free of visible noise or artifacts. This is exactly what was observed in the reported results. For example, under the no-polarizer condition, E-39-W-2-S-30 produced acceptable demodulation even though W = 2 is below the conservative deterministic range, because the camera still captured about 56% correct-phase information, and the demodulated output satisfied the visual acceptance criteria. By contrast, under the polarizer condition, E-213-W-0-S-30 failed, even though about 81% correct-phase information was present, because the reduced light energy degraded the modulated image and therefore demodulated image quality. However, E-213-W-1-S-30 became acceptable (i.e., with an increase of just 4% correct-phase data) with approximately 85% correct-phase information. These results show that acceptable demodulation depends not only on phase correctness but also on signal energy behavior. Therefore, the purpose of the evaluation criteria was not only to redefine the deterministic synchronization bound but also to establish a practical benchmark for understanding how different synchronization conditions affect what the camera actually records and how that, in turn, affects demodulation quality. In this sense, the calculated W = 3 to 4 values provide safer and more deterministic operating points, whereas the lower experimentally acceptable W values reveal the tolerance of the system under less conservative conditions. This is a useful finding rather than a weakness of the criteria, because it shows that the proposed study not only identifies a deterministic synchronization setting but also characterizes the trade-off between acquisition speed and demodulation quality. This level of insight is important for users who may wish to choose between a more conservative synchronization margin and a faster operating point depending on the imaging scenario.

Since one of the central claims of this work is that the proposed HyperSI synchronizer is designed as a compact and portable embedded prototype, its physical form factor should be compared against the desktop-based synchronizer used in the SOTA HSPy-SI system. This comparison is important because the form factor directly affects system integration, portability, setup complexity, and suitability for space-constrained experimental environments. Using the approximate dimensions from Section 4.3, the proposed embedded synchronizer consists of an RPI 4 Model B with a volume of $90\times 53\times 22=104,940\;{\mathrm{mm}}^{3}$ and a Zybo Z7-20 SoC board with a volume of $122\times 88\times 15=161,040\;{\mathrm{mm}}^{3}$, giving a total bare-board volume of $265,980\;{\mathrm{mm}}^{3}$. If a packaging tolerance of 200% is included to account for enclosure space, wiring, connectors, and support integration overhead, the effective volume of the embedded synchronizer becomes $265,980\times 3=797,940\;{\mathrm{mm}}^{3}$, which is equivalent to approximately $7.98\times 10^{-4}\;m^{3}$. In contrast, the SOTA HSPy-SI synchronizer described in Section 3.1 uses a desktop with an approximate volume of $400\times 165\times 370=24,420,000\;{\mathrm{mm}}^{3}$, or $2.442\times 10^{-2}\;m^{3}$. Therefore, even after accounting for the 200% enclosure tolerance, the proposed embedded architecture reduces the synchronizer volume by approximately 96.7% relative to the desktop-based SOTA system, demonstrating a substantial improvement in compactness and portability.

The present prototype has been experimentally validated at a single spatial frequency of $0.122\;{\mathrm{mm}}^{-1}$, and the claims made in this manuscript are therefore restricted to single-frequency operation. However, the proposed HyperSI pattern-generator hardware IP was intentionally designed as a parameterized and deterministic real-time video-streaming block, in which runtime arguments such as spatial_frequency do not alter the synthesized throughput of the design. Since the hardware maintains 1 PPC operation at an 86 MHz clock for a fixed projector resolution, the frame-generation timing remains deterministic for the implemented architecture across the supported spatial frequency parameter range of $0.001\;\mathrm{to}\;0.999\;{\mathrm{mm}}^{-1}$. In addition, the DLP projector operates in video mode by locking to the incoming HDMI frame timing and maintaining its deterministic dual-buffer latency mechanism, which depends on frame timing rather than on the spatial content of the projected pattern. Therefore, although multi-frequency performance has not yet been experimentally demonstrated in this manuscript, the proposed prototype already possesses the architectural capability to support future experiments with multiple spatial frequencies without modification of the synchronization framework.

Table 2 includes different approaches using SI and multi-spectral or HS imaging acquisition systems to be compared in terms of their acquisition time. The table includes the citation of the study where the system is described, the year it was published, how many spatial frequencies were used, the number of wavelengths measured as well as the spectral range, how many phases or patterns were used for every wavelength, the total number of images projected and captured, the amount of time necessary to acquire all projected patterns, and the synchronizer system-build form factor. Note that the column “phases per wavelength” refers to how many patterns of SI were used per wavelength measured.

Table 2 shows that HyperSI occupies a distinct position among existing SFDI systems by combining the conventional TPD workflow, broad HS coverage, and an explicitly engineered frame-level synchronizer within a compact embedded architecture. Compared to earlier TPD-based systems, which typically use 2–11 spatial frequencies, 4–34 wavelengths, and acquisition times ranging from 3.6 s to 150 s per dataset [19,20,22,23,31], HyperSI retains the standard TPD model while reducing the modulated acquisition rate (i.e., in FPS) by achieving 12 modulated HS cubes per second over 24 bands from 660 to 950 nm for one experimental spatial frequency. This makes it, to the best of the authors’ knowledge and among the studies compared in Table 2, the fastest reported TPD-based HS SFDI synchronizer while still preserving conventional TPD. In contrast, the higher-speed systems in the literature achieve their rates mainly by departing from the TPD itself, for example, through SSOP, cSFDI, or temporally multiplexed single-pattern acquisition [16,30,54]. Although such approaches provide clear speed advantages, they trade away the conventional TPD framework and are therefore subject to the known limitations of single-shot methods, including lower spatial resolution, edge artifacts, motion sensitivity, and reduced reconstruction fidelity compared with phase-stepped SFDI (Section 1). HyperSI instead addresses speed without abandoning TPD while also providing a synchronizer form factor that is qualitatively different from those of prior desktop-, cart-, and SW-sequenced systems [19,20,22,23]. Furthermore, the pattern-generator IP in the SoC exposes the arguments tof_distance, phase, spatial_frequency, throw_ratio, and brightness as scalar control registers through an AXI4-Lite slave interface. As a result, these parameters are configurable at runtime from the embedded application in the PS, rather than being fixed constants in hardware. Although the present experiment was conducted using only one spatial frequency, the parameterized IP design allows the same hardware to be reconfigured for different spatial frequencies in future experiments, together with the other exposed parameters, without requiring hardware redesign. Its decoupled portable embedded synchronizer, compatibility with HDMI- and USB-based projection-imaging setups, deterministic frame-level synchronization, and tunable compensation for projector dual-buffer latency together make it more viable for TPD-based real-world applications, where compactness, timing determinism, interoperability, and real-time operation are all important.

To the best of the authors’ knowledge and based on the studies summarized in Table 2, HyperSI represents the fastest reported implementation of the TPD-based portable bench-top real-time SI prototype in terms of modulated capture rate while also providing explicit frame-level synchronization with the tunable delay control parameter W. Unlike previous TPD-based implementations that typically rely on fixed SW delays, the proposed system explicitly guarantees frame-level projection–capture synchronization, enabling deterministic and configurable timing control during acquisition.

## 7. Conclusions

A frame-accurate high-performance SI synchronizer has significant potential in applications where precise timing between pattern projection and image capture is critical. In biomedical and biophotonic imaging, this synchronization can support future quantitative imaging workflows that require stable phase capture and repeatable demodulation. Since the proposed system is compared with several SOTA SFDI methods reported by other authors (Table 2), it can be considered a promising experimental candidate for future biomedical SI studies. However, its use in clinical or tissue-optical-property applications will require further validation with appropriate phantoms, biological samples, and application-specific imaging protocols. With such validation, the synchronizer may support future studies related to non-invasive tissue assessment, perfusion and oxygenation monitoring, and intraoperative imaging, where stable and time-accurate acquisition is important. Beyond biomedical imaging, the proposed synchronizer is also relevant to profilometry and structured-light 3D measurement. In fringe projection profilometry, accurate synchronization ensures that each camera frame corresponds to the intended projected phase pattern. This is important for high-speed surface reconstruction, dynamic object measurement, vibration analysis, deformation monitoring, and industrial 3D inspection. The same timing accuracy can benefit robotic vision, automated quality control, material inspection, manufacturing calibration, and optical metrology systems, where repeatable, high-speed, and artifact-reduced measurements are required. Thus, the proposed synchronizer is not limited to one SI application field but can serve as a general synchronization platform for TPD/TPS-based SI systems.

The proposed architecture adopts a heterogeneous control model in which the SBC acts as the master controller responsible for camera triggering and data storage, while the SoC operates as a slave responsible for deterministic pattern generation. Although this design provides modularity and simplifies system integration, it introduces an inherent timing limitation in the trigger path. Specifically, the capture trigger propagates from the SoC frame synchronization event through GPIO signaling to the SBC and is subsequently handled by SW running on a non-real-time operating system before issuing a USB trigger to the camera. Such an SW-mediated control path can introduce non-deterministic latency and jitter, which are well-known challenges in embedded systems relying on general-purpose operating systems. This behavior explains the observation that the first interrupt may originate from a previous phase and necessitates the use of a larger W parameter to provide a safety margin that ensures stable phase capture. However, increasing W directly increases the capture delay, thereby limiting the achievable frame rate of the overall system. Future work will focus on reducing this timing uncertainty by introducing a more deterministic hardware-based triggering mechanism. One potential improvement is to implement a direct hardware trigger path from the SoC to the camera, allowing the capture signal to be generated synchronously with the pattern frame events detected in the programmable logic. Alternatively, the system architecture could be restructured so that the SoC or FPGA acts as the master controller, coordinating both projection and camera triggering with hardware-level synchronization. Such approaches would eliminate the non-deterministic SW layer in the trigger path, reduce latency and jitter, and enable higher-frame-rate operation for real-time SI imaging applications. Furthermore, the pattern-generation frequency can be experimented with beyond the current 60 Hz, as the DLP projector supports up to 120 Hz. Another direction involves integrating real-time processing capabilities on the SBC or SoC, such as performing demodulation followed immediately by machine learning-based classification. This would enable intelligent on-device decision-making for SI imaging applications.

## Figures and Tables

**Table 1:** Post-implementation resource utilization breakdown for xc7z020clg400-1.

HW IP Blocks	LUTs	FF	DSP48E1	BRAM	LUTRAM	IO	BUFG	PLL
*Pattern_kernel*	8634	7479	73	0	143	0	0	0
*VTC*	77	118	0	0	0	0	0	0
*AXI4S-VOut*	127	173	0	1	0	0	0	0
*RGB-to-DVI converter*	158	142	0	0	0	0	2	1
*PS*	0	0	0	0	0	0	1	0
*Others*	1955	2350	0	0	12	0	0	0
**Total**	**10,951** **(20.58%)**	**10,262** **(9.64%)**	**73** **(33.18%)**	**1** **(0.71%)**	**155** **(0.89%)**	**28** **(22.40%)**	**3** **(9.38%)**	**1** **(25.00%)**

**Table 2:** Comparison of representative SFDI studies in terms of spatial frequencies, wavelength sampling, phase measurements, total number of images, acquisition time, and synchronizer form factor.

Study	Year	Spatial Freq.	Wavelengths Measured (Spectral Range)	Phases Per Wavelength	Total Images	Acquisition Time	Synchronizer System Form Factor
Wavelength optimization for rapid chromophore mapping using spatial frequency domain imaging [19]	2010	2	34 (650–980 nm)	3	204	50 s	Desktop SW-sequenced fixed-delay acquisition
First-in-human pilot study of a spatial frequency domain oxygenation imaging system [23]	2011	2	6 (670–980 nm)	3	369	3.6 s	A mobile cart/computing system containing the computer and control electronics with SW-timed synchronization acquisition
Preoperative mapping of nonmelanoma skin cancer using spatial frequency domain and ultrasound imaging [20]	2014	11	4 (590–740 nm)	3	132	13 s	Desktop LabVIEW SW-controlled sequential acquisition
Real-time absorption reduced surface fluorescence imaging [32]	2014	1 real-time (0.5 ${\mathrm{mm}}^{-1}$; 4 evaluated)	1 excitation band (490 nm; >510 nm emission)	3	3	≈19 FPS	TPD; desktop DLP cyclic 3-phase
Real-time simultaneous single snapshot of optical properties and blood flow using coherent spatial frequency domain imaging (cSFDI) [16]	2016	1	1 (660 nm)	1 (using SSOP)	1	50 FPS	Uses SSOP; hence single-shot static-pattern illumination, no TPD
qF-SSOP: real-time optical property corrected fluorescence imaging [54]	2017	1	2 reflectance (760 and 808 nm); 1 fluorescent (>815 nm)	1	3	10 ms demodulation speed for the 2 reflectance wavelengths	Desktop single-shot pattern acquisition
Preclinical evaluation of spatial frequency domain-enabled wide-field quantitative imaging for enhanced glioma resection [22]	2017	8	6 (390–632 nm)	3	144	12 s	Desktop SW-sequenced DMD capture
High-speed spatial frequency domain imaging with temporally modulated light [30]	2017	1	3 (519–740 nm)	1 spatial pattern/1 phase-equivalent image per wavelength; recovered after temporal demultiplexing	128	128 frames at 55.6 frames/s ≈ 2.3 s	Static transparent film for single-phase projection and wavelength recovered on desktop
Hyperspectral imaging in the spatial frequency domain with a supercontinuum source [31]	2019	1 spatial frequency (0.1 ${\mathrm{mm}}^{-1}$)	580–950 nm	3	≈3000 frames (i.e., 1000 spectral steps × 3 phases)	150 s per HS cube; less than 1 s per modulated capture; hence ≈3 s for 3 phases	MCU-triggered scan synchronization
Accurate burn wound categorization using an optimized spatial frequency domain imaging device and machine learning (Commercial product: Clarifi) [28]	2024	1 (0.12 ${\mathrm{mm}}^{-1}$)	470–850 nm; 5 planar wavelengths at 470, 525, 620, 730, and 850 nm; only one 850 nm wavelength as modulated capture with only 1 spatial frequency 0.12 ${\mathrm{mm}}^{-1}$)	1 for every wavelength, and additionally 3 only for 850 nm wavelength	8	10 s per full scan	Not reported in details since it is a proprietary product
HyperSI	2026	1 (programmable & parameterizable)	24 (660–950 nm)	3 (programmable & parameterizable)	30 modulated HS cubes (as much as user wants depending of disk-space size)	12 modulated HS cubes per s	Decoupled portable embedded TPD synchronizer from Projector-Imaging (PIG) system (no desktop or bulky computing system management); compatible with HDMI- and USB-based PIG setups; deterministic frame-level sync; tunable delay to compensate for projector dual-buffer latency

**Figure A1:**
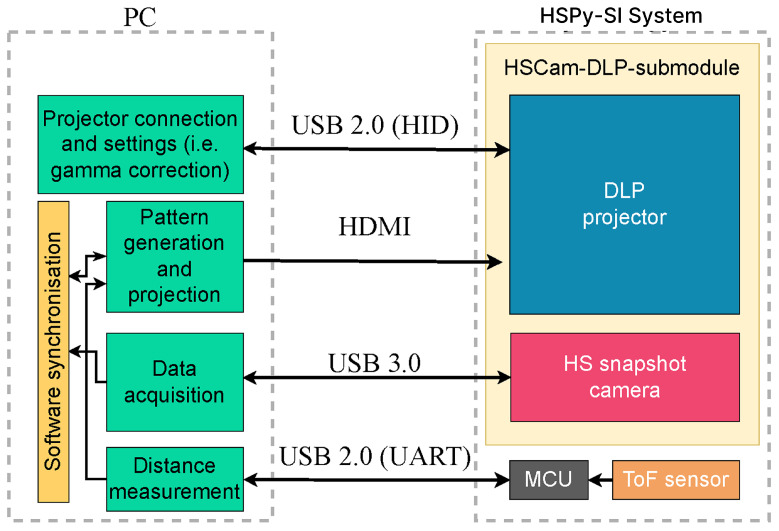
A schematic of the HSPy-SI system showing the connections between all the components with their functionalities.

**Figure A2:**
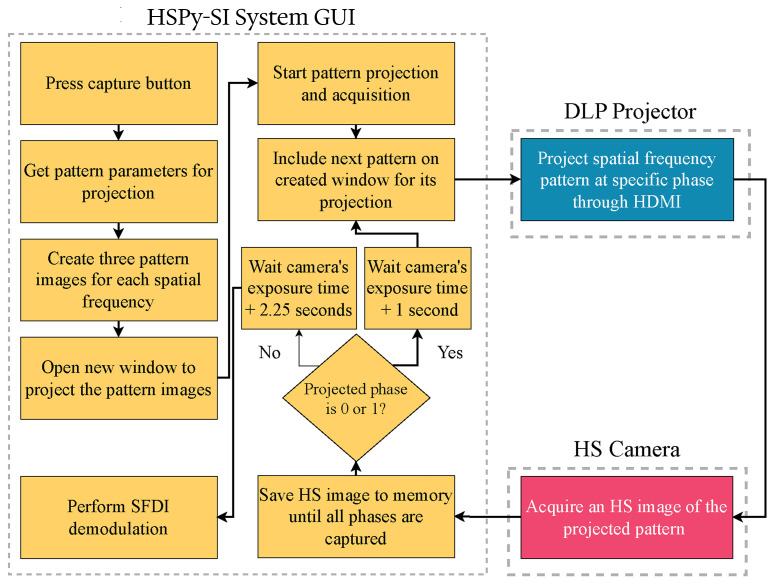
An operational workflow diagram of the HSPy-SI system. The diagram illustrates the sequence of operations involved, including pattern generation, projection, image acquisition, and demodulation. The process is managed through a GUI that coordinates the projection of phase-shifted patterns via a DLP projector and the corresponding image acquisition by the HS camera.

**Figure A3:**
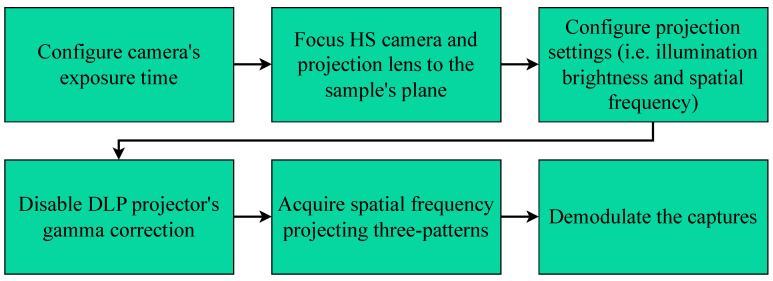
A workflow diagram for configuring and acquiring spatial frequency projection measurements with the HSPy-SI system for the conducted experiments.

**Figure A5:**
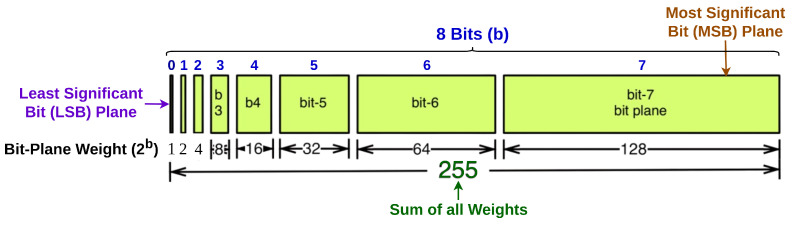
Bit division within a frame for an 8-bit monochrome image, showing the LSB plane at b=0 (marked in purple) and the MSB plane at b=7 (marked in brown), along with the corresponding binary weights and their total sum of 255 (marked in green) [45].

**Figure A6:**
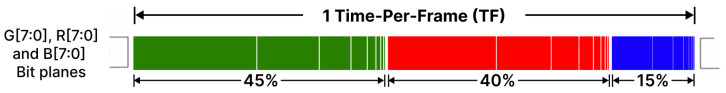
The arrangement of GRB bit-planes within a TF. The green, red, and blue channels occupy 45%, 40%, and 15% of the 1 TF duration, respectively. Each color channel consists of eight bit-planes arranged in order of decreasing weight (i.e., b7 to b0).

**Figure A10:**
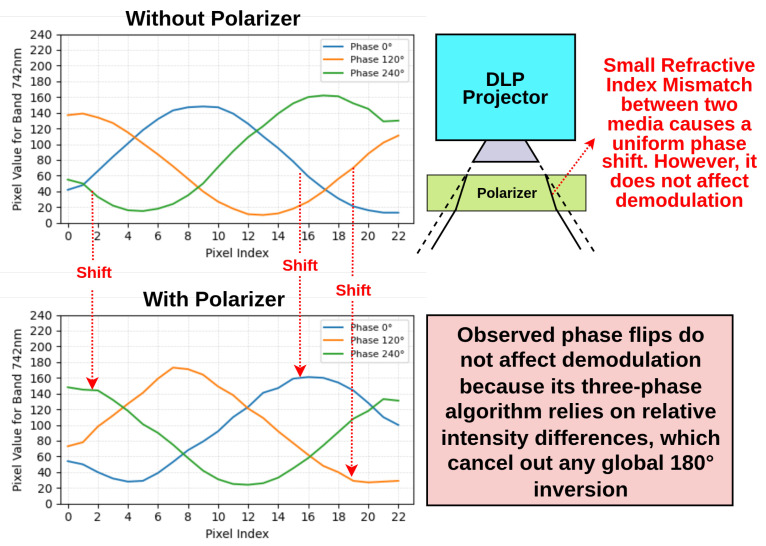
A small refractive-index mismatch after applying the polarizer. When light passes through the polarizer, its path slightly shifts (red mark), but this small refraction does not affect the demodulation Equation (6).

**Figure A11:**
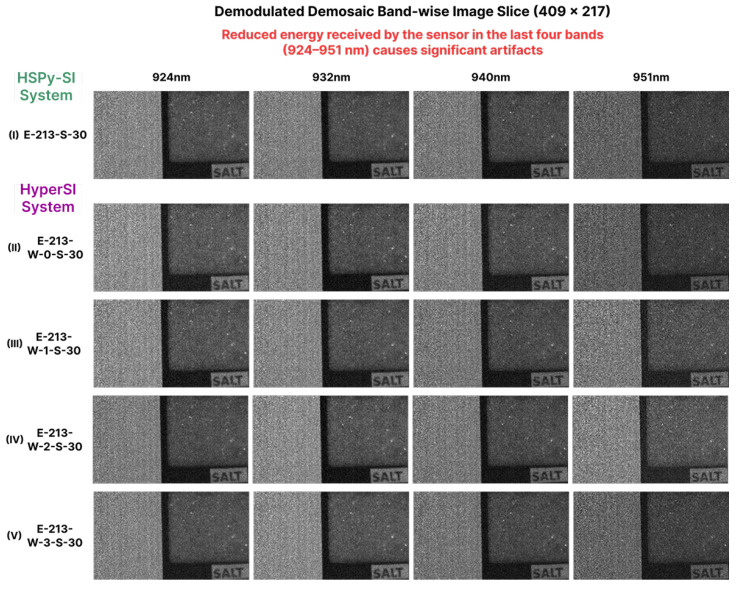
Strong artifacts are visible in the last bands, 924 nm, 932 nm, 940 nm, and 951 nm, for the demosaiced demodulated captures (i.e., 409×217) using a polarizer (i.e., 213 ms exposure period).

**Figure A12:**
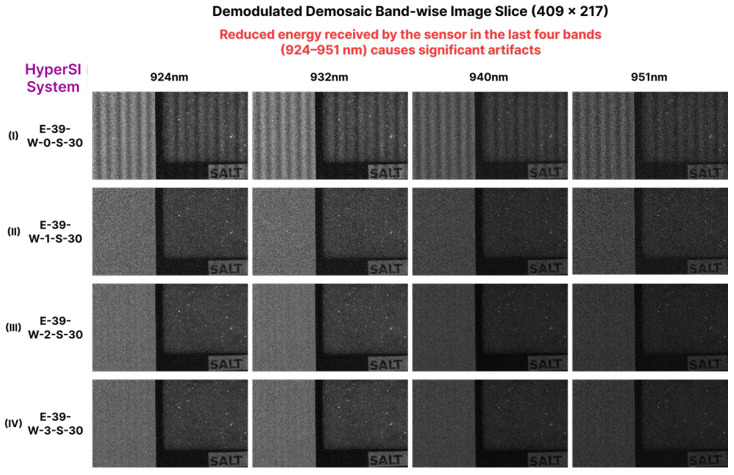
Strong artifacts are visible in the last bands across different W = 0, 1, 2, 3 configurations, 924 nm, 932 nm, 940 nm, and 951 nm, for the demosaiced demodulated captures (i.e., 409×217) using the no-polarizer setting (i.e., 39 ms exposure period).

**Figure A15:**
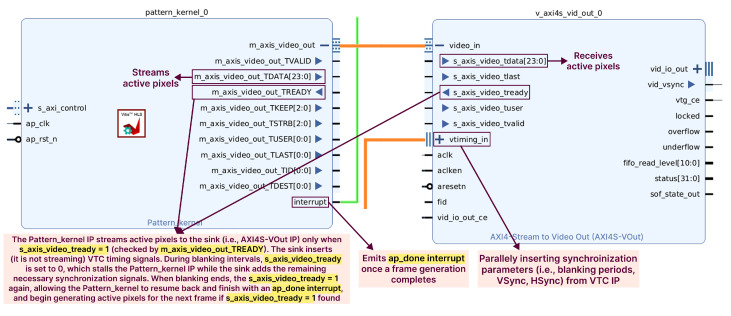
The illustrated HW design is a zoomed-in view of the AXI4-Stream interface between the Pattern_kernel (i.e., the pattern-generator IP) and the AXI4S-VOut IP, extracted from the complete HW system design for the SoC. The downstream AXI4S-VOut IP core induces backpressure on the Pattern_kernel IP so that the generator stalls until the AXI4S-VOut IP finishes adding a blanking period to the active pixel stream. Therefore, the measured period for the interrupt coming from the Pattern_kernel IP is always equal to the TF (i.e., 17.26 ms).

**Figure A16:**
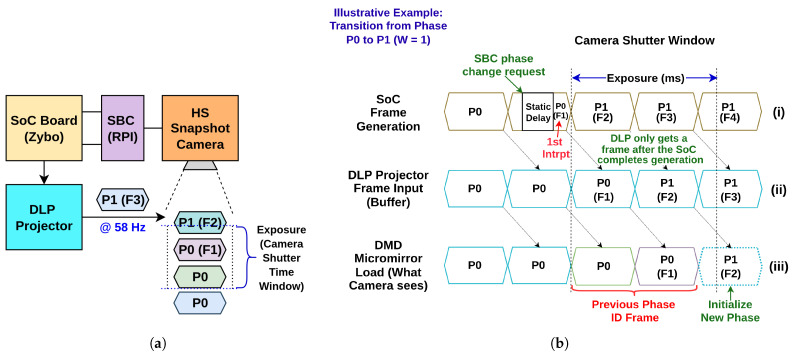
The camera’s perspective with respect to SoC frame generation and DLP projection for the phase transition case P0 to P1. (**a**) Explains the real scenario: The HS camera sees one projected frame at a time. However, frame initialization and projection occurring immediately after receiving frames generated by the SoC cannot be explained from this perspective, especially when the 48-bit display frame buffer of the DLPC350 controller introduces a frame lag. (**b**) For a better illustration, this shows how the camera actually sees the frames for each particular phase ID, which cannot be explained by (**a**). Row (**i**) shows the SoC frame-generation timeline. In row (**ii**), frames are received by the display buffer of the projector after generation is done by the SoC. Row (**iii**) illustrates the micromirror initialization of the frame (marked as a dotted frame), lagging one frame behind the SoC (Section 4.1). During the exposure period, the camera records whatever the DLP is projecting, one frame at a time.

**Figure A18:**
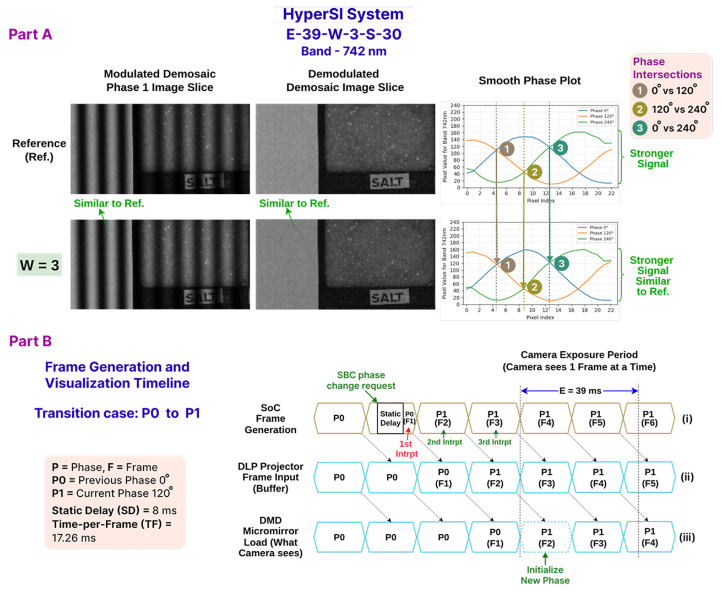
For the E-39-W-3-S-30 configuration, in Part (**A**), transition from phase P0 to P1 is demonstrated through captured modulated images and their corresponding demodulated results compared to their references. The SPP curves indicate that the signal is stronger or weaker. In Part (**B**), row (**iii**) demonstrates what the camera sequentially recorded: the correct-phase initialization P1(F2) (dotted frame), P1(F3) and a partial P1(F4). Since all captured frames belong to the correct phase, the modulated image in Part (**A**) is close to the reference (marked in green). Also, SPP markers (1–3) align with the reference, with strong signal intensity. Consequently, the demodulated image appears clean. This configuration meets the acceptance criteria; it is accepted.

**Figure A19:**
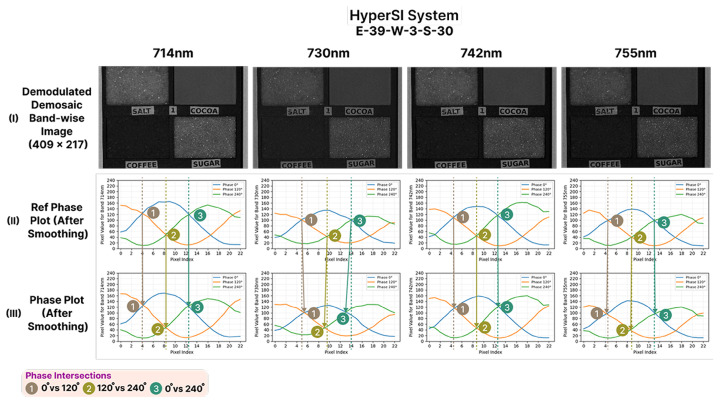
The demodulated capture by the HyperSI system for the E-39-W-3-S-30 configuration, with its SPP phase-shift comparison against the reference. The four spectral bands (714 nm, 730 nm, 742 nm, and 755 nm) are arranged in columns.

**Figure A21:**
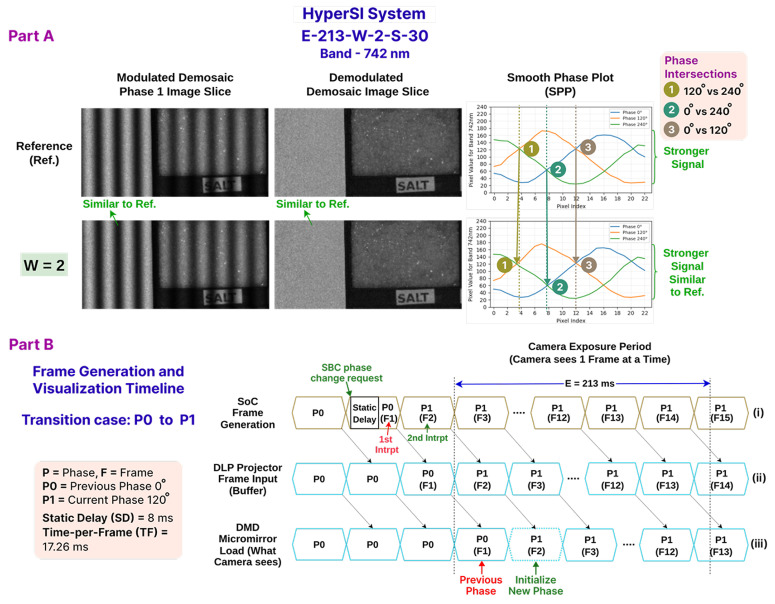
For the E-213-W-2-S-30 configuration, in Part (**A**), transition from phase P0 to P1 is illustrated through captured modulated images and their corresponding demodulated results compared to their references. The SPP curves indicate that the signal is stronger or weaker. In Part (**B**), row (**iii**) illustrates that the camera recorded ≈13 frames: incorrect phase P0(F1), the correct-phase initialization P1(F2), stable phases P1(F3) to P1(F12), and a partial P1(F13). The modulated image and the SPP markers are close to the reference. The demodulated image appears clean. This configuration meets the acceptance criteria; it is accepted.

**Figure A22:**
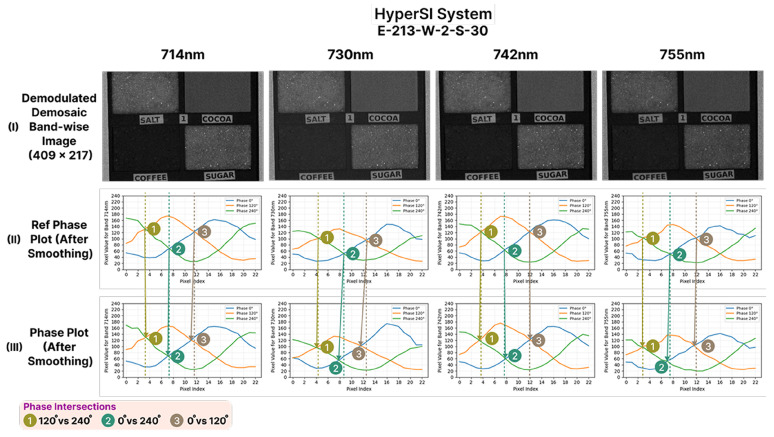
The figure shows the demodulated capture by the HyperSI system for the E-213-W-2-S-30 configuration, with its SPP phase-shift comparison against the reference. The four spectral bands (714 nm, 730 nm, 742 nm, and 755 nm) are arranged in columns.

**Figure A23:**
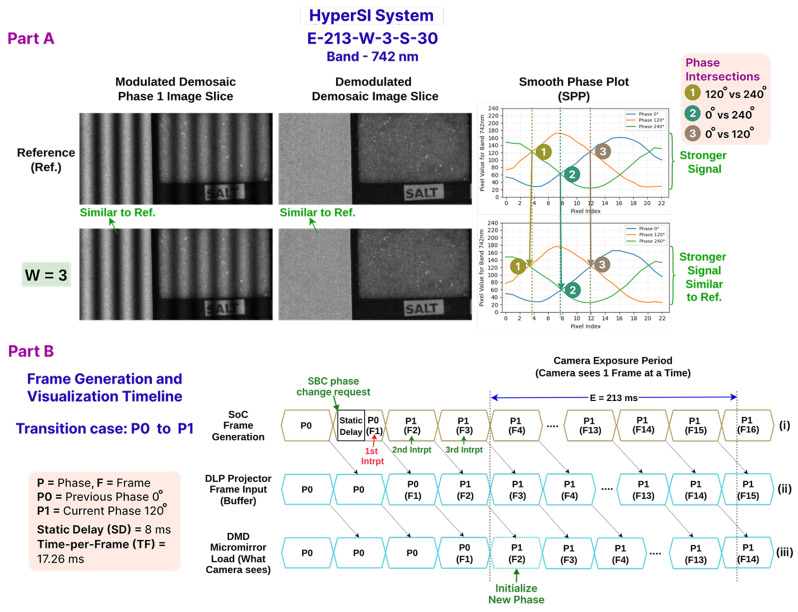
For the E-213-W-3-S-30 configuration, in Part (**A**), transition from phase P0 to P1 is demonstrated through captured modulated images and their corresponding demodulated results compared to their references. The SPP curves indicate that the signal is stronger or weaker. In Part (**B**), row (**iii**) shows that the camera recorded ≈13 frames: the correct-phase initialization P1(F2), stable phases P1(F3) to P1(F13), and a partial P1(F14). The modulated image and the SPP markers are close to the reference. The demodulated image appears clean. This configuration meets the acceptance criteria; therefore, it is accepted.

**Figure A24:**
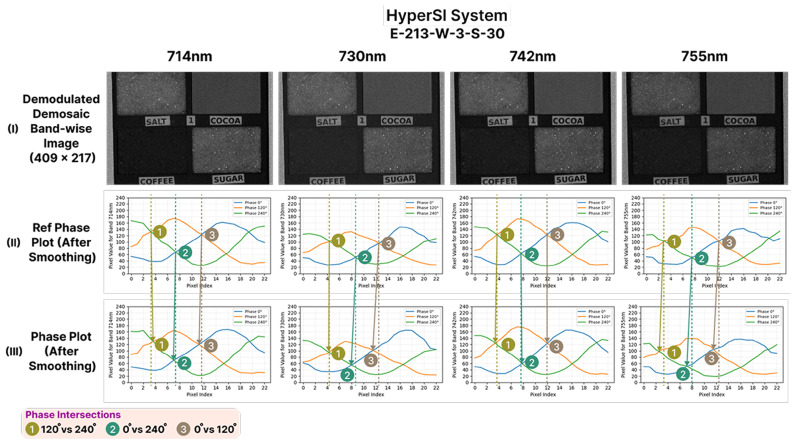
The demodulated capture by the HyperSI system for the E-213-W-3-S-30 configuration, with its SPP phase-shift comparison against the reference. The four spectral bands (714 nm, 730 nm, 742 nm, and 755 nm) are arranged in columns.

## Data Availability

The original contributions presented in this study are included in the article. Further inquiries can be directed to the corresponding authors.

## Abbreviations

The following abbreviations are used in this manuscript:

ACK	Acknowledgment
ACT	Actual Capture Time
ARSFi	Absorption-Reduced Surface Fluorescence Imaging
AXI4S-VOut	AXI4-Stream to Video Out
CC	Color Channel
CI	Confidence Interval
CPT	Change Pattern Time
cSFDI	Coherent Spatial Frequency Domain Imaging
DLP	Digital Light Processing
DMD	Digital Micromirror Device
FOV	Field of View
FPD	Flat Panel Display
FPS	Frames per Second
FRR	Frame Refresh Rate
GPIO	General-Purpose Input/Output
GRB	Green Red Blue
HLS	High-Level Synthesized
HS	Hyperspectral
HSPy-SI	HS Python-based SI
HW	Hardware
ISR	Interrupt Service Routine
LCTF	Liquid Crystal Tunable Filter
LLG	Liquid Light Guide
LSB	Least Significant Bit
MCU	Microcontroller Unit
MoC	Model of Computation
MSB	Most Significant Bit
NIR	Near-Infrared
OS	Operating System
PCC	Pearson Correlation Coefficient
PIG	Projector-Imaging
PL	Programmable Logic
PP	Phase Plot
PPC	Pixels per Clock
PS	Processing System
PWM	Pulse-Width Modulation
RMSE	Root Mean Square Error
ROI	Region of Interest
RPI	Raspberry Pi
SBC	Single-Board Computer
SBI	Spectral Band of Interest
SD	Static Delay
SFDI	Spatial Frequency Domain Imaging
SI	Structured Illumination
SoC	System-on-Chip
SOTA	State-of-the-Art
SPP	Smoothed Phase Plot
SSOP	Single Snapshot imaging of Optical Properties
ST	Saving Time
SW	Software
TF	Time per Frame
TR	Throw Ratio
TMDS	Transition-Minimized Differential Signaling
ToF	Time of Flight
TPD	Three-Phase Demodulation
TPS	Three-Step Phase Shifting
TT	Total Time
TX	Transmitter
VESA	Video Electronics Standards Association
VTC	Video Timing Controller
W	Frame Count to Wait
WXGA	Wide Extended Graphics Array

## Appendix A. SOTA HSPy-SI System

### Appendix A.1. Supplementary Details on HSPy-SI Components

**DLP Projector:** The DLP^®^ LightCrafter^™^ E4500 MKII^™^ Fiber Couple (EKB Technologies Ltd., Bat Yam, Israel) has been chosen as the candidate projector model; it supports video mode with a 24-bit RGB HDMI pattern input stream. This model includes the DLPC350 digital controller (Texas Instruments Inc., Dallas, TX, USA) to facilitate the control of the integrated near-infrared (NIR) DMD [33]. The projector features a video display with up to a wide extended graphics array (WXGA) resolution (1280×800 pixels), a throw ratio of 1.2, an on-axis projection, and a pattern rate of up to 120 Hz for 8-bit grayscale patterns. In addition, video can be sent through an HDMI cable, which changes the dimensions of the DMD to 912×1140 pixels.

**HS Camera:** The projector used was suitable to acquire data in the NIR range using the second-generation MQ022HG-IM-SM5X5-NIR2 HS snapshot camera (Ximea GmbH, Münster, NRW, Germany), which captures 24 spectral bands from 665 to 960 nm in a single exposure. The raw 2D image captured with the camera has dimensions of 2048×1088 pixels, whose active area reduces the dimensions to 2045×1085 pixels. Within that area, all spatial and spectral information is stored in 5×5 pixel mosaics for 25 wavelengths. This 2D image can be transformed into an HS cube with dimensions 409×217×24 by applying a spectral correction matrix specified by the manufacturer. The camera model features a USB 3.0 interface to ensure a high-speed data bus with the host computer. Moreover, the camera has a fixed-focal-length lens VIS-NIR C 35 mm (Edmund Optics, Barrington, NJ, USA).

**Light Source, Bulb, and Liquid Light Guide (LLG):** The Fiber-Lite Mi-150 light housing (Dolan-Jenner, Boxborough, MA, USA) LLG, with 150 watts of power, has been selected as the light source. And an L1090 halogen lamp (International Light Technologies Inc., Peabody, MA, USA) has been used as the light bulb to illuminate the projection through the projector. This specific bulb was selected because it provides more energy within the NIR spectral capture by the camera compared to the default lamp included in the light housing. The transmission of photons from the light housing to the DMD was facilitated by using an LLG5-4Z liquid light guide (Thorlabs Inc., Newton, NJ, USA) featuring a 5 mm diameter core, which exhibited optimal compatibility with the projector through the fiber coupler.

**Polarizers:** The HSPy-SI system uses two polarizing filters: one in front of the projector lens and the other attached to the camera lens. This configuration is essential to mitigate reflections in captured images. The polarizer, when positioned in front of the projector, directs the emitted light to polarize in a particular direction. When this polarized light illuminates the surface, reflected light of the same polarization contributes to the reflection. A second polarizer, positioned in front of the camera lens, is at a perpendicular, or crossed, angle relative to the first. This ensures that only light that has undergone a significant polarization change, typically from tissue, passes through the polarizer of the camera. Light with the original polarization of the projector’s emission is effectively blocked by the cross-polarized camera filter. A $2^{\prime \prime }$ diameter model (LPNIRE200-B, Thorlabs Inc., Newton, NJ, USA) was used for the projector, which covers 600–1000 nm, and a $1^{\prime \prime }$ model (LPNIRE100-B, Thorlabs Inc., Newton, NJ, USA) was used for the camera lens.

**Time-of-Flight (ToF) Sensor and MCU:** To measure the working distance, a VL53L0X ToF laser-ranging sensor (STMicroelectronics, Geneva, Switzerland) has been selected. A NodeMCU ESP8266 (Espressif Systems, Shanghai, China) MCU is used to manage and control the ToF sensor.

**The PC:** A desktop computer synchronizes the projection–capture and controls all the components of this SFDI system. It has a 12th Gen Intel^®^ Core^™^ i5-12400 × 12 processor with a 2.50 GHz base clock and 32 GB of RAM. The computer runs on an Ubuntu 20.04LTS OS.

### Appendix A.2. Supplementary Details on HSPy-SI System Design

Figure A1 shows the connections of different core components used for the HSPy-SI system. This system integrates a DLP projector with DMD, an HS snapshot camera, a ToF sensor (controlled by an MCU to measure working distance), a desktop computer (i.e., PC), and a light source with a lamp for broadband illumination. The HS camera and DLP projector form the reusable HSCam-DLP-submodule, ensuring the modularity of implementation across different SFDI systems. The DLP projector receives the pattern from the PC as a video stream through HDMI with a resolution of 912×1140. Furthermore, the DLP projector is connected to the PC through a USB 2.0 (HID) peripheral connected to pre-load different configurations (such as disabled gamma correction). A USB 3.0 cable is used to connect the HS camera to the PC during pattern projection for data acquisition. The aperture of the camera was configured to ≈f1.65 for this study to allow more light to pass through the sensor, thus allowing the camera to reduce the exposure time and therefore the acquisition time.

The DLP projector receives the pattern from the PC as a video stream through HDMI with a resolution of 912×1140. The changing patterns for three different phases are controlled by the PC (i.e., the desktop computer). Note that a further description of how the projector is synchronized with the HS camera is included in Section 3.3. Specifically, the DLPC350 employs a dual-buffer memory configuration, with each buffer accommodating 24-bit planes of image data. Simultaneously, one buffer is displayed on the DMD, while the other one is populated with new frame data from the HDMI interface. The double-buffering mechanism introduces a minimum delay of one frame between input and projection in video mode, which is the standard DLP configuration for the HSPy-SI system; therefore, the displayed frame always corresponds to the frame received previously [33]. Furthermore, a USB 2.0 peripheral is used to connect the system to the PC to configure the DLP and eliminate nonlinearity of the system, thus ensuring the right projection of sinusoidal patterns.

The USB 3.0 cable is used to connect the HS camera to the PC during pattern projection for data acquisition. The aperture of the camera has been configured to ≈f1.65 for this study to allow more light to pass through the sensor, thus allowing the camera to reduce the exposure time and therefore the acquisition time.

To measure the working distance, the PC controls the ToF sensor through the MCU. The light source feeds the light through the LLG to the DLP projector to illuminate the projection surface.

### Appendix A.3. Operational Workflow

The operational workflow of the SOTA HSPy-SI system considers the incorporation of polarizers on both the DLP projector and the HS snapshot camera. These polarizers eliminate specular reflections, ensuring that only the diffuse reflectance of the scene is captured. Figure A2 outlines the operational workflow of the system, which comprises three subsystems: the GUI for user control, the DLP projector for pattern projection, and the HS camera for image acquisition.

Upon pressing the capture button, the system retrieves projection parameters and, for each spatial frequency, generates and displays three phase-shifted patterns on the projection surface. For phases 0 and 1, it waits for a duration equal to the camera exposure time plus one second; for the final phase, it waits for a duration equal to the exposure time plus 2.25 s to accommodate an unknown amount of latency caused by the DLP projector and OS overhead. Static delays of 1 and 2.25 s were determined by trial and error to achieve projection–capture synchronization in the polarizer setup. This three-phase loop ensures that all patterns are captured without artifacts, with each HS image stored in memory until the set is complete. Once all phase images are acquired, SFDI demodulation is performed using Equation (6).

### Appendix A.4. Capture Methodology

The SOTA HSPy-SFDI system acquires three phase-shifted modulated images in sequence and then demodulates them to produce a single demodulated image. Continuous real-time acquisition is not supported; instead, each button press initiates a cycle of three modulated captures followed by a single demodulation. The workflow presented in Figure A3 illustrates the methodology followed to acquire the captures for the experiments carried out in this study.

It includes the configuration of the system, specifically involving an HS camera and a DLP projector. The process commences with the adjustment of the camera’s exposure time, followed by the focusing of both the HS camera and the projection lens onto the sample plane. Subsequently, the projection parameters are configured, including the illumination brightness and spatial frequency. In addition, the DLP projector’s gamma correction is disabled to ensure accurate light modulation of the sine-wave patterns. The subsequent workflow involves the acquisition of spatial frequency data by displaying three-phase patterns on any given sample. Ultimately, the captured images are demodulated, and the reflectance of the sample is calibrated using the white reference data.

## Appendix B. Supplementary Details on Materials and Methods

### Appendix B.1. Supplementary Details on Latency Caused by the DLP Projector to Refresh a Pattern Frame

The DLPC350 Programmer’s Guide [45] and related datasheets have been thoroughly examined to explore the micromirror reorientation and stabilization process. These references provide a theoretical foundation for understanding how bit-plane time-slot allocation and micromirror actuation contribute to the overall frame stabilization time period. It can be explained for an example case at a standard 60 Hz Frame Refresh Rate (FRR) for a 24-bit incoming image frame. Since frame stabilization is performed by the DMD using the entire frame period, it is essential to know the frame-generation frequency (i.e., 60 Hz in this case). The DLPC350 controller automatically measures the frame period (i.e., TF) by locking the VSync pulse [33]. Hence, $TF_{60\mathrm{Hz}}$ can be computed using Equation (A1), as shown in Equation (A2).

(A1) $$TF_{FRR}=\frac{1}{FRR}$$

(A2) $$TF_{60\mathrm{Hz}}=\frac{1}{60}=0.01667\;s=16.67\;\mathrm{ms}$$

The 24-bit RGB image frame contains three color channels (i.e., red, green, and blue). Each color channel has an 8-bit depth, i.e., 8 bits of intensity information, illustrated in Figure A4. The video output modes operate on a per-frame basis. To span the three channels throughout the frame period, the DLPC350 controller assigns specific percentages to each of those channels; i.e., time slots are allocated for one image pixel as 40% for red, 45% for green, and 15% for blue [45]. The time slots allocated for each of the channels are then distributed among eight bit-planes [33,45]. A bit-plane is a two-dimensional array containing one specific bit (i.e., from bit 0 up to bit 7) extracted from every pixel in the full-color image. The DMD receives each of these bit-planes from the buffer and sends them to the micromirrors one at a time throughout the entire frame stabilization period. In both parallel RGB (i.e., HDMI in this study) and FPD-Link input modes, the DLPC350 assigns the color channels in the default order of green, red, and then blue (i.e., GRB) [33]. The color channel (CC) time slot $T_{CC}$ can be represented using Equation (A3);

(A3) $$T_{CC}=TF\left(\mathrm{ms}\right)\times \left(\frac{X}{100}\right)\;\left[\begin{array}{c} X\;=\;\mathrm{color}\;\mathrm{allocation}\;\\ \mathrm{percentage}\;(e.g.,\;45\;\mathrm{for}\;\mathrm{green}) \end{array}\right]$$

By substituting the individual color channel percentage values into Equation (A3), the time durations for each channel are obtained, as shown in Equations (A4a)–(A4c). However, only the green channel will be further analyzed, because the same logic applies to red and blue.

(A4a) $$\begin{array}{cc} T_{Green} & =16.67\times \left(\frac{45}{100}\right)=7.50\;\mathrm{ms} \end{array}$$

(A4b) $$\begin{array}{cc} T_{Red} & =16.67\times \left(\frac{40}{100}\right)=6.67\;\mathrm{ms} \end{array}$$

(A4c) $$\begin{array}{cc} T_{Blue} & =16.67\times \left(\frac{15}{100}\right)=2.50\;\mathrm{ms} \end{array}$$

To distribute the allocated time of the green channel (i.e., 7.50 ms) through its bit-planes, the DLPC350 uses a technique called Pulse-Width Modulation (PWM). PWM controls the average power of a signal by varying the width of its pulses at a fixed frequency. It effectively simulates analog output using a digital signal. The DLPC350 controller implements the PWM technique to achieve a similar objective. PWM regulates the display duration of each bit-plane according to its binary weight. Thus, depending on the bit weight of each bit-plane, the DLPC350 controls the timing for each bit-plane to be exposed to light. Hence, the illumination duration is directly proportional to the binary weight of the bit-plane [45]. Therefore, the time duration/slots allocated for each color channel are divided among its corresponding bit-planes in proportion to their binary weights. The time slot of the green channel $T_{Green}$ = 7.50 ms is distributed over its 8 bit-planes accordingly. And the period of time allocated per bit-plane $T_{b}$ is proportional to the weight unit and is calculated as $2^{b}$ of its corresponding bit position *b*, as expressed by Equation (A5). Here, binary weight refers to the relative importance of each bit in an 8-bit binary number. The bits are indexed from b=0 (i.e., LSB, minimum intensity contribution) to b=7 (i.e., MSB, maximum contribution). Consequently, higher-order bit-planes are displayed for longer durations to render brighter pixel intensities [45]. In summary, higher bit-planes (closer to MSB) are allocated longer display durations to represent brighter intensities.

(A5) $$T_{b}\propto 2^{b}\;[\mathrm{where}:\;b\in \left[0,7\right]]$$

Since each bit-plane contributes a binary weight of $2^{b}$, the sum of weights across all 8 bit-planes equals 255, which is expressed using Equation (A6). Figure A5 also visually illustrates the binary weight distribution between bit-planes, highlighting the positions of the MSB and LSB, their weights $2^{b}$, and the total sum of 255.

(A6) $$\sum_{b=0}^{7}2^{b}=2^{0}+2^{1}+2^{2}+2^{3}+2^{4}+2^{5}+2^{6}+2^{7}=255$$

The total weight of 255 provides a reference to proportionally allocate time durations for each bit-plane, ensuring that more significant bits are displayed longer than less significant ones. In this process, the total time slot assigned to a color channel (e.g., 7.50 ms for green) is divided among its 8 bit-planes in proportion to their binary weights. Each bit-plane is allocated a fraction of the total time of the channel based on its binary weight $2^{b}$ relative to the total sum of the weights of all bit-planes (i.e., 255) so that the higher-weighted planes occupy a larger share of the allotted channel time. Therefore, the individual bit-plane duration $T_{b}$ could be calculated using the generalized Equation (A7a). Below, the specific computations for the green, red, and blue channels are given by Equations (A7b)–(A7d) and are then listed in Table A1.

(A7a) $$\begin{array}{cc} T_{b} & =\left(\frac{2^{b}}{255}\right)\times T_{CC} \end{array}$$

(A7b) $$\begin{array}{cc} T_{bg} & =\left(\frac{2^{b}}{255}\right)\times T_{Green} \end{array}$$

(A7c) $$\begin{array}{cc} T_{br} & =\left(\frac{2^{b}}{255}\right)\times T_{Red} \end{array}$$

(A7d) $$\begin{array}{cc} T_{bb} & =\left(\frac{2^{b}}{255}\right)\times T_{Blue} \end{array}$$

**Table A1:** This table presents the 8 bit-plane indices from 0 to 7 (first column), the corresponding binary weights (second column), and the approximate time durations for each bit-plane of the green, red, and blue channels (third, fourth, and fifth columns). The sixth column (GRB Total) shows the row-wise sum across the three color channel durations for each bit-plane. The last row shows the total binary weight of 255, the total time for each channel (green ≈ 7.50, red ≈ 6.67 and blue ≈ 2.50 ms), and the GRB total time of 16.67 ms, obtained by summing the eight bit-plane durations from each column.

8-Bit-Plane Information		Bit-Plane Time			
Bit Index (*b*)	Weight ($2^{b}$)	Green ($T_{\mathrm{bg}}$)≈	Red ($T_{\mathrm{br}}$)≈	Blue ($T_{\mathrm{bb}}$)≈	GRB Total≈
0	1	0.0294	0.0262	0.0098	0.0654
1	2	0.0588	0.0523	0.0196	0.1307
2	4	0.1176	0.1046	0.0392	0.2614
3	8	0.2353	0.2092	0.0784	0.5229
4	16	0.4706	0.4185	0.1569	1.046
5	32	0.9412	0.8370	0.3137	2.0919
6	64	1.8824	1.6740	0.6275	4.1839
7	128	3.7647	3.3480	1.2549	8.3676
**Total Sum**	**=255**	**7.50** ms	**6.67** ms	**2.50** ms	**16.67** ms

By adding up the durations of all 24 bit-planes across the three channels, the total time for the DMD micromirrors to reorient and stabilize for one frame is 1 TF, as shown in Equation (A8b).

(A8a) $$\begin{array}{cc} \mathrm{Total}\;\mathrm{Frame}\;\mathrm{Time} & =T_{Green}+T_{Red}+T_{Blue}\;\;\;\;\;\;\;\;\;\;\;\;\;\;\;\;\;\;\;\;\;\;\;\;\;\; \end{array}$$

(A8b) $$\begin{array}{cc} \mathrm{Total}\;\mathrm{Frame}\;\mathrm{Time} & =7.50+6.67+2.50=16.67\;\mathrm{ms}=TF \end{array}$$

Equation (A8b) also validates that, during the first frame F1 of the micromirror timeline in Figure 7b, the DMD micromirrors underwent reorientation and initialization within the TF duration, and the image fully stabilized in the subsequent frame.

Figure A6 also illustrates how the bit-planes of each color channel (GRB, according to the DLPC350 default color channel order) are streamed to the DMD. For each color segment, only one bit-plane is streamed at a time, proceeding from the MSB to the LSB. The MSB planes are exposed for longer durations as a result of their higher binary weights, whereas the LSB planes are displayed for shorter periods, contributing proportionally less to the final image intensity. However, the DLPC350 intertwines and interleaves bit-planes, per-color time slots, and color frames to improve image quality. In video mode, this behavior is automatic and not user-configurable [45]; i.e., since the DLPC350 controller is proprietary technology, the exact color and bit-plane interleaving sequence are not provided by the datasheet.

### Appendix B.2. Supplymentary Details on HyperSI Components

**Raspberry Pi as the SBC:** A Raspberry Pi (RPI) 4 Model B is used as the SBC-based system. It carries a 64-bit quad-core ARM Cortex-A72 (ARM v8) processor that operates at about 1.8 GHz clock frequency; the board provides two USB 3.0 ports, which makes it a suitable candidate for interfacing and controlling an HS camera. A standard 40-pin GPIO header allows GPIO communication with other systems, such as an SoC-based platform. The board also includes two micro-HDMI ports and a microSD card slot for the operating system and data storage. The used RPI 4 Model B has an approximate form factor (i.e., dimensions) of 90 mm × 53 mm × 22 mm (length × width × height).

**Digilent Zybo Z7-20 Development Board as the SoC Board:** The Digilent Zybo Z7-20: Zynq-7000 ARM/FPGA SoC development board model (Austin, TX, USA) uses the AMD Xilinx Zynq-7020 SoC (XC7Z020-1CLG400C) from AMD Xilinx (Santa Clara, CA, USA). The SoC combines a dual-core ARM Cortex-A9 Processing System (PS) that can run at a clock frequency of up to 667 MHz and a PL unit. The PL offers 53,200 lookup tables, 106,400 flip-flops, and 630 KB of block RAM, along with other HW resources. These resources are sufficient for the complete HW design, including the High-Level Synthesized (HLS) pattern-generator IP core. The board carries an HDMI TX port that outputs 24-bit RGB video from the SoC. This output can stream real-time pattern frames to an external display, for example, a DLP projector, or to a monitor. The board has 6 PMOD ports that can be used for the GPIO interface with external systems (e.g., with an SBC). In addition, the board includes 1 GB DDR3L with a 32-bit bus @ 533 MHz (1066 MT/s), which is essential for running an HLS accelerator platform to evaluate the pixel values generated by the HLS pattern-generator IP core.

**Digilent PMOD ToF Sensor:** The Digilent PMOD ToF module is used for low-power optical distance measurement. Its ISL29501 chip contains a digital signal processor that records the round-trip time of light that is sent towards a target and reflected back, converting that time into distance. This model uses an 860 nm near-IR wavelength of light for distance measurement. The module can detect objects from 300 mm up to 5000 mm away.

**HS Snapshot Camera and DLP Submodule:** The HS-Cam-DLP submodule shown in Figure A1 mounts the HS snapshot camera and the DLP projector. It was used in the SOTA HSPy-SI system and reused in the proposed SFDI system. The submodule includes an HS snapshot camera, a DLP projector, polarizers, a light source, and an LLG.

**Miscellaneous:** Different types of cables are used for respective specific interfaces. Jumper wires connect the Raspberry Pi GPIO pin header to the Zybo board’s PMOD ports. An HDMI 1.4 specification cable connects the HDMI TX port of the Zybo board to the DLP projector.

### Appendix B.3. Operational Workflow

This section provides a detailed overview of the system’s operational workflow. Whereas the SOTA HSPy-SI workflow uses polarizers, the proposed system can perform captures with and without them. Polarizers remove specular reflections and also attenuate target light, resulting in longer camera exposures at the cost of real-time performance. Without polarizers, the camera can capture comparable energy using minimal exposure time at the cost of specular reflections. The determination of the exposure time, both with and without polarizers, is described in Section 5.1.2. Then, both polarizer configurations were tested and evaluated using the proposed system. Figure A7 explains the operational workflow of the proposed system in two stages: the process boot stage and the capture loop stage.

In step 1 of the boot stage, the SoC embedded application configures all pattern-generation parameters, such as ToF distance measurement, spatial frequency, throw ratio, and brightness. The press of a push button on the SoC board then triggers ToF distance acquisition and sends the measurement directly to the pattern-generator IP. Alternatively, a static value can be entered in the pattern-controller software of the SoC. The ToF sensor has been positioned outside the camera’s FOV. To preserve capture integrity, it should be turned off once the distance measurement is passed to the pattern generator, preventing unintended near-infrared interference. Once configured, the SoC board initiates the pattern-generation process and streams the pattern frames to HDMI for phase ID 0 by default. Since the SBC acts as the master controller, in step 2, all experimental configuration parameters should be set there, such as camera exposure time, number of modulated sample captures, and W = Frame Count to Wait. W is the waiting period in terms of an integer pattern frame interrupt count. In step 3, the SBC initializes all the GPIO configurations needed to communicate with the SoC. All GPIO communications between the SBC and the SoC follow a half-duplex communication mode. In step 4, the SBC uses GPIO to set W to the SoC. Next, in step 5, the SBC instructs the SoC to load pattern phase ID 0 to reset any residual phase values from previous tests (e.g., typically phase ID = 2). After that, in step 6, the HS camera is initialized with all the experimental parameters.

After finishing the initial boot configuration, the system starts warming up the SoC application to have a stable interrupt period for the first phase change (i.e., P0 to P1) in the capture loop stage. The system cycles through each phase ID {0,1,and2} using the phase-change technique described in Section 4.2. After each phase change, an HS image is captured but is not saved before entering the main capture loop. A fixed delay replaces the actual HS image save to emulate its time-consumption behavior. The delay (i.e., “dummy” save) ensures that the phase-change timing path in the warm-up cycle aligns the phase-change timing path in the real capture loop stage.

Finally, the system enters the capture loop stage. To change the phase of the pattern in the SoC, the system repeats the same phase-change technique as in step 5 and the DLP warm-up loop. Next, the SBC takes one modulated HS image capture. This loop is performed for S number of modulated captures and saves them to the disk. After performing all S modulated captures, the SBC scans the disk and performs demodulation using Equation (6).

### Appendix B.4. Supplementary Details on Develop the HW/SW System in the SoC Environment

The HW/SW system for the SoC board has been developed using three state-of-the-art AMD Xilinx tools from the 2022.2 release [55]. Vitis HLS software, v2022.2 synthesizes the C/C++ High-Level HLS pattern-generator kernel (i.e., Pattern_kernel) code (Algorithm A1) into an HW IP. The IP integrator of the Vivado Design Suite facilitated the assembly and interconnection of all HW IPs into the complete HW design. Then the Vitis IDE was used to generate the platform from the complete HW design and also to write C drivers and methods to initiate the pattern projection application in the PL from the PS.

#### Appendix B.4.1. Supplementary Details on Select the Pattern Projection Resolution and FRR

In video mode, the DLP projector supports resolutions up to 1280×800 pixels at a refresh rate of 120 Hz FRR via the 30-bit RGB or FPD-Link interfaces. However, the SOTA HSPy-SI system has two resolution choices, 1280×800 and 912×1140, due to the limitation on the recognition of resolution by the Linux OS. The SOTA system’s manufacturer has chosen the latter, i.e., 912×1140 @ 60 Hz FRR, because it allowed the system to detect the output of the DLP projector as a second monitor.

Therefore, the 912×1140 pixel pattern resolution was also chosen for the SoC application to keep both systems alike for fair-comparison purposes. And, 60 Hz FRR was selected to ensure that the HW design can be tested on the widest range of retail monitors currently available.

#### Appendix B.4.2. Supplementary Details on Determine HW Operating Clock Frequency and VTC Parameters

The primary function of the HW design is to drive the pattern-generator HLS IP (Section 4.6.4). Since the HW was designed from scratch to generate pattern frames with the maximum FRR performance possible, the first task was to find the HW operating clock frequency for the complete HW design. To achieve this, the initial step was to calculate the pixel clock frequency and the VTC parameters. Next, the necessary PPC requirement was retrieved from the Digilent HDMI IP. After that, the population of the VTC parameters was done. And finally, combining these results, the HW core clock was determined.

**Determine Initial Pixel Clock Frequency:** The pixel clock frequency $Pixel_{clock}$ (i.e., pixels/s) dictates how fast the pattern-generator IP must emit pixels, including active and blanking intervals. The blanking period refers to the portion of each video line (horizontal blanking) or each frame (vertical blanking) during which no active pixel data are transmitted. Calculating the operating clock frequency of the HW for the final design begins by determining $Pixel_{clock}$. In addition, a fairly accurate pixel clock is essential to ensure reliable video output and HW timing closure. The VESA [56] defines open timing standards that specify exactly how to calculate pixel clocks, total pixels, and blanking intervals for arbitrary resolutions and FRRs, ensuring that custom video modes generated via compliant tools are recognized and displayed correctly by compliant display devices (e.g., monitors, projectors). A VESA-compliant cvt command from Linux Ubuntu 24.04LTS [57] was used to calculate the pixel clock frequency $Pixel_{clock\_cvt}=86$ MHz and the actual $FRR_{actual\_cvt}=59.78$ Hz by specifying a resolution of 912×1140 and the target FRR, $FRR_{target}=60$ Hz, as command arguments. In particular, this command prefers 50.0, 60.0, 75.0 or 85.0 Hz as the $FRR_{target}$ options. The cvt command gives a “Modeline” for a given resolution and $FRR_{target}$. The Modeline is universal VESA-compliant HW timing data. From the generated Modeline, the total horizontal pixels and the total vertical pixels, including their blanking period, can be found as, respectively, $H_{total}=1216$ and $V_{total}=1183$. The $Pixel_{clock\_cvt}$ and $FRR_{actual\_cvt}$ can be verified using Equations (A10) and (A11) by calculating $Pixel_{clock}$ and the estimated actual frequency $FRR_{est\_actual}=59.78$. In addition, the used cvt command uses the pixel clock formula of an older version than the current version (1.2). Hence, it rounds the ideal 86.312 MHz clock to the nearest 0.25 MHz (i.e., 86.00 MHz), which can be seen in Equation (A10). Therefore, $Pixel_{clock\_cvt}=86$ MHz will be used in the next steps.

(A9) $$pixels_{per\_frame}=H_{total}\times V_{total}=1216\times 1183=1,438,528$$

(A10) $$Pixel_{clock}=pixels_{per\_frame}\times FRR_{target}=86,311,680\;\mathrm{Hz}=round\left(86.312,0.25\right)\approx 86.00\;\mathrm{MHz}$$

(A11) $$FRR_{est\_actual}=\frac{Pixel_{clock}}{pixels_{per\_frame}}=59.78\;\mathrm{Hz}$$

**Determine the PPC Requirement from the HDMI IP:** The PPC count defines how many pixel samples the HDMI block accepts at each input clock tick. Changing PPC alters only the internal data path width and the input clock frequency, not the external Transition-Minimized Differential Signaling (TMDS) bitrate. So, the input clock frequency must be equal to the pixel rate. Thus, the PPC count requirement of the HDMI IP plays an important role in the HW clock design decision, as well as the pattern-generator IP PPC configuration. The Digilent RGB-to-DVI HDMI IP core encodes 24-bit RGB video data, along with the pixel clock and synchronization signals (i.e., VTC parameters), and drives the raw TMDS clock and data channels as defined by the DVI 1.0 source-device specification. By default, the Digilent HDMI IP (i.e., RGB-to-DVI Video Encoder IP) has been configured for 1 PPC. That is, 1 PPC means that one pixel sample will be transferred at each input clock tick. Therefore, given the initially calculated pixel clock of 86 MHz, the HDMI IP simply uses the same 86 MHz as its operating clock.

**Determine the VTC Parameters:** The Modeline generated by cvt depends not only on the input resolution but also on $FRR_{target}$. Because the CVT’s blanking scheme (front-porch, sync, back-porch) is partly tied to the $FRR_{target}$, the horizontal and vertical parameters of the VTC are changed to maintain stable porches at a higher horizontal frequency $H_{frequency}$ (i.e., line rates; how many video lines are drawn per second; each pattern frame contains $H_{frequency}$/$V_{total}$ lines). Therefore, Equation (A12) explains the HSync: 70.72 kHz value generated from the cvt command, i.e., when an HSync pulse is being issued.

(A12) $$H_{frequency}=\frac{Pixel_{clock}}{H_{total}}=\frac{86\;\mathrm{MHz}}{1216}\approx 70.72\;\mathrm{Hz}$$

And finally, since the PPC required by the HDMI IP is 1, the population of VTC parameters is straightforward. Table A2 reorganizes and populates all the VTC parameter values found from the cvt-command-generated Modeline.

**Table A2:** VTC IP configuration values for 912 × 1140 pixels @ 60 Hz refresh rate.

Parameter	Horizontal Settings	Frame/Field 0 Vertical Settings	Frame/Field	Horizontal Fine Adjustment
Active Size	912	1140	Vblank Start	1140
Frame Size	1216	1183	Vblank End	1183
Sync Start	968	1143	VSync Start	1143
Sync End	1064	1153	VSync End	1153

**Determine HW Operating Clock Frequency:** The PPC = 1 requirement also simplifies the $HW_{clock}$ calculation. The $Pixel_{clock\_cvt}$ found by the Linux cvt can be directly used as the HW clock $HW_{clock},$ shown by Equation (A13)

(A13) $$HW_{clock}=Pixel_{clock\_cvt}=86\;\mathrm{MHz}$$

This clock will be set on the Zynq 7000 PS IP and the pattern-generator IP. Thus, the entire HW design runs on a single clock domain set by the 86 MHz clock frequency.

#### Appendix B.4.3. Supplementary Details on Configure the VTC IP

Before the HW design, configuration of the VTC IP is an important step since it generates the precise synchronization signals (e.g., HSync, VSync, blanking intervals) and clock domains needed to drive video signals at the correct resolutions and refresh rates required by an output display device.

The VTC LogiCORE IP from AMD Xilinx functions as a configurable video timing controller, enabling the generation of synchronization signals for a broad spectrum of video formats, standard and custom types. In this prototype, the VTC LogiCORE IP has been specifically employed to support a non-standard video format for the custom 912×1140 pixel resolution required by the used DLP projector. This IP provides two modes, detection and generation, to handle the acquisition and creation of the timing signals needed for video display. In this design, the generation mode has been used to produce the timing signals required to drive the display with custom resolution through HSync and VSync pulses, based on the configured timing parameters. The VTC parameters defined in Table A2 have been used to configure the VTC IP.

#### Appendix B.4.4. Supplementary Details on Design and Develop Pattern-Generator HLS IP

Since the HDMI IP used requires a 1 PPC rate for the pixel feed, the pattern-generator HLS IP (i.e., Pattern_kernel()) has been structured to allow dataflow modeling through function, loop, and stream pipelining techniques to achieve 1 PPC performance. This IP generates a pattern with a resolution of 912×1140, where the width and height are, respectively, 912 and 1140, as required by the DLP projector. Since the entire HW design has to operate at an 86 MHz clock frequency, the top-level function Pattern_kernel() (i.e., IP kernel) is synthesized as a real-time video-streaming HW IP with an 86 MHz clock. The pseudocode to generate pixel streams within the PL fabric is given in the pseudocode Algorithm A1.

**Algorithm A1** Pattern_kernel HLS IP
1: **function** Compute… (out_stream_obj, in_stream_obj, {scalar_arg1, scalar_arg2, …}) 2: **for** i←0 **to** height − 1 **do** 3: #pragma HLS PIPELINE 4: **for** j←0 **to** width − 1 **do** 5: // Compute... 6: **end for** 7: **end for** 8: **end function** 9: **procedure** Pattern_kernel( m_axis_video_out, tof_distance, phase, spatial_frequency, throw_ratio, brightness ) 10: // HLS interfaces 11: #pragma HLS INTERFACE S_AXILITE PORT = {tof_distance, phase, spatial_frequency, throw_ratio, brightness, return} 12: #pragma HLS INTERFACE AXIS REGISTER BOTH PORT = m_axis_video_out 13: #pragma HLS STREAM VARIABLE = {S1, S2, S3, S4, S5, S6, S7} 14: #pragma HLS DATAFLOW 15: // intermediate stream objects S1...S7 16: ComputeProjectorWidth(S1, throw_ratio, tof_distance) 17: ComputeProjectorWidthWithPI(S2, S1, spatial_frequency) 18: ComputeWaveIntermediateInputValue(S3, S2) 19: ComputeWaveInput(S4, S3, phase) 20: ComputeCosineWaveValue(S5, S4) 21: ComputePixelValue(S6, S5, brightness) 22: ComputeGrayToRGB(S7, S6) 23: xf::cv::XFMat2AXIvideo(S7, m_axis_video_out) 24: **end procedure**

The pseudocode decomposes all computations in Equation (5) into seven subfunctions, each prefixed with the name COMPUTE.... This structure enables fine-grained control over the latency and Initiation Interval (II), supports task-level pipelining and concurrency, improves hardware resource sharing and binding, decouples control and data dependencies, and simplifies verification and reuse. The processed pixels are streamed from one subfunction to the next in each iteration of the induction variable j, which can be found at line 4 of the pseudocode Algorithm A1.

Line 11 declares the AXI4-Lite slave port for each scalar argument tof_distance, phase, spatial_frequency, throw_ratio, brightness and the implicit return of the Pattern_kernel IP. As a result, those arguments become accessible as memory-mapped registers from the PS (i.e., CPU or host), thus enabling this IP to be a completely controllable parameterized IP from the embedded application residing in the PS.

Line 12 declares m_axis_video_out as an AXI-Stream master interface. The REGISTER BOTH directive option insert registers are on both the input and output sides of the stream channel to meet the timing and FIFO backpressure requirements.

All loops involved in computing the pixel values across the tasks achieved an II of 1, thus ensuring a real-time dataflow model of computation (MoC). To reach II = 1 while keeping the generated cosine values as close as possible to their ideal approximations, every stream object and variable used in arithmetic operations is declared with the Vitis HLS library fixed-point data types, namely, ap_fixed<Total_Bits, Integer_Bits> and ap_uint<Integer_Bits>, which use fixed-point arithmetic. The value 28 is used as the scaling factor to map real numbers to scaled integers, enabling maximum fractional precision in fixed-point format.

A custom wrapper StreamContainer() class tuned for HLS has been written to create stream objects by wrapping these fixed-point datatypes, inspired by the Xilinx Vision Library-provided xf::Mat() class. It is a templated HLS-friendly data container that holds pixel data in on-chip/block RAM, supports streaming interfaces, and carries its own metadata, such as height, width, and PPC. It implements a 1D array in HLS-friendly BRAM, with a single data[] buffer. So, the HLS tool can help allocate exactly the right depth of FIFOs or partition BRAMs, resulting in a smaller, more efficient hardware implementation and better timing than hand-rolled arrays. The default value 2 has been used as the FIFO depth, and 1 is used for PPC. The pragma directive at line 13 declares all the stream_objects (i.e., S1$\ldots $S7) as STREAM VARIABLE directive option, wrapped by the custom StreamContainer().

The preparation of the input for the mathematical cos() function is scaled up between subfunctions from line 16 to line 19. The ComputeCosineWaveValue() function computes the input and generates the cosine value with the AMD Xilinx-provided hls::hotbm:cosf() function.

The ComputePixelValue() function writes the resulting per-pixel intensity values in the stream S6. The ComputeGrayToRGB() function consumes the gray 8-bit pixels of the single channel of S6 and expands or maps them to the 24-bit three-channel RGB format, as required by the Digilent HDMI IP, pushing full-color pixels into the stream S7 for downstream stages.

xf::cv::XFMat2AXIvideo() is an AMD Xilinx-provided function that converts S7 to an AXI4-Stream video format. It generates the required handshake signals (TVALID, TREADY) along with the payload and sideband signals (TDATA, TUSER, TLAST) and drives them on the m_axis_video_out interface toward the video sink. With respect to the HW design (Figure A8), the video sink is the AXI4S-VOut IP, which consumes the AXI4-Stream of pixels produced by the pattern-generator IP. This interface will be further analyzed and explained in Section 5.5.3 to understand the behavior of the interrupt timing.

Finally, this IP generates each cosine pattern at 1 PPC speed.

#### Appendix B.4.5. HW Design

To simplify the illustration of the HW design, IPs are grouped into Vivado hierarchies based on their functional responsibilities within the proposed system. Figure A8 illustrates the simplified interface view of the complete HW design from the Vivado IP integrator. The design operates at an 86 MHz clock frequency, as already determined in Section 4.6.2. This clock rate provides sufficient bandwidth for pixel data transfer and maintains precise synchronization across all video components, including the HDMI output. For simplicity, most IPs used in the design are wrapped in different groups according to their responsibility in the application.

The Xilinx Zynq-7000 PS processing_system7_0 HW IP serves as the main controller of the total design and the source of the 86 MHz clock. The PS provides the main system clock FCLK_CLK0 and distributes a global reset signal FCLK_RESET0_N to all modules in the PL, ensuring synchronous operation. It communicates with other IP cores via the M_AXI_GP0 interface, utilizing an AXI SmartConnect interconnect IP (wrapped in the PS_reset_interconnect group) to support multiple AXI master and slave ports.

The video stream, generated by the IP Pattern_kernel (wrapped inside the group Pattern_Change_Handler), is processed by an AXI4S-VOut IP v_axi4s_vid_out_0, as indicated by the pink connections. This IP also adds the necessary synchronization signals for the image display. The VTC IP (i.e., v_tc_0), configured for the target resolution and refresh rate, provides these synchronization signals. The video data stream is then passed to rgb2dvi_0, a Digilent RGB-to-DVI converter IP (i.e., the HDMI IP), which formats the data into TMDS for HDMI transmission.

The Wait_Frame_Count_Handler group handles the GPIO communication with the SBC that is responsible for setting up W = Frame Count to Wait in the embedded SoC application.

The Pattern_Change_Handler group manages the GPIO communication SBC through the AXI GPIO IP, as well as the pattern-generator Pattern_kernel HLS IP. This group is responsible for solving the projection–capture synchronization, which will be discussed in detail in Section 4.8.

The ToF group deals with IIC communication with the ToF sensor that is connected through the PMOD ports. The Button_Handler group defines the control of a push button that is responsible for the interactive control of the ToF sensor.

#### Appendix B.4.6. Supplementary Details on Embedded SoC Application (SW)

After synthesizing and generating the HW bitstream, an HW platform needs to be built. Using the HW platform, the embedded SW was compiled. The application runs on the first core of the ARM processor. After turning on the Zybo board, this application boots with the default values for the arguments tof_distance, phase, spatial_frequency, throw_ratio, brightness of the Pattern_kernel IP. By default, the application executes the pattern-generation kernel for phase ID 0, along with other arguments given above, and streams the pattern to the output display device (a monitor for testing or the DLP projector). An interactive push button is also programmed in the application to obtain the distance measurement from the ToF sensor and load it into the Pattern_kernel in real time. It also initiates and registers all the Interrupt Service Routines (ISRs) written for the ToF sensor module and GPIO communication with the SBC-based system. The ISR (Algorithm 1) that is directly responsible for projection–capture synchronization has been discussed in detail in Section 4.8.

### Appendix B.5. The SW System in the SBC Environment

The SW system in the SBC environment acts as the master controller by controlling the pattern projection on the SoC board through GPIO, taking HS image captures, and finally saving them to the disk. The SBC column segment of the operational workflow shown in Figure A7 is presented as a pseudocode in Algorithm A2.

**Algorithm A2** Main Function
1: **function** Main 2: // System boot and initial setup 3: // 1. Initialize SBC GPIO API 4: InitSBCGPIO() 5: // 2. Wait so the SoC can finish booting and send its initial pattern 6: WAIT(x milliseconds) 7: // 3. Configure the W frame count on the SoC 8: sendWaitFrameCountToSoC(W) 9: // 4. Reset the pattern phase on the SoC to zero 10: sendAndRcvPhaseIdToSoC(0) 11: // 5. Initialize the HS camera config parameters 12: InitHSCamConfig(EXPOSURE_TIME, GAIN) 13: // 6. Enter the SoC warm-up stage to stabilize the interrupt period for the final capture loop. 14: **for** i←0 **to** 2 **do** 15: // 6.1 Send the phase ID (cyclically 0, 1, 2) to the SoC 16: sendAndRcvPhaseIdToSoC((i mod 3)) 17: // 6.2 HS image capture via software trigger 18: captureHSImage(hs_image, SOFTWARE_TRIGGER) 19: // 6.3 “Dummy” Save 20: sleep(STATIC_DELAY) 21: **end for** 22: // 7. Enter the capture loop for S number of samples 23: **for** i←0 **to** S−1 **do** 24: sendAndRcvPhaseIdToSoC((i mod 3)) 25: captureHSImage(hs_image, SOFTWARE_TRIGGER) 26: // Save the captured image to disk 27: save(hs_image) 28: **end for** 29: **end function**

#### Appendix B.5.1. Boot Stage

First, it configures the GPIO API parameters for the initial setup. Then it waits (line 6) for a few milliseconds to allow the SoC board to complete the initial boot. At line 8, sendWaitFrameCountToSoC(W) sends the Wait Frame Count W to the SoC board through GPIO and waits until it receives the ACK. This tells the SoC to set W in the global environment. Then the sendAndRcvPhaseIdToSoC(0) function call tells the SoC to send the pattern to the DLP projector for phase ID 0 as the initial configuration for the pattern projection. After that, InitHSCamConfig(EXPOSURE_TIME, GAIN) initializes the Ximea camera API with important experimental parameters required, such as EXPOSURE_TIME and GAIN for the HS camera. To address the inconsistent first interrupt period observed during the initial phase change in the second iteration (i.e., P0 to P1) of the capture loop, a warm-up stage was introduced. This inconsistency arises because the very first transition from phase P0 to P1 occurs at an arbitrary moment, while a frame from the previous phase is still being processed. As a result, the first interrupt period becomes highly volatile, ranging from approximately 1.74 ms to 12.37 ms across all trials, as observed in the experiment detailed in Section 5.5.3 and reported in Table A3. By executing a dedicated warm-up loop prior to the actual capture loop, the timing of the first phase transition is aligned with a more predictable window. In actual modulated capture experiments, this resulted in the first interrupt period typically falling within a narrower range of 4.06 to 5.16 ms, with only one isolated occurrence at 10.6 ms (Figure 13b). Thus, the SoC warm-up loop effectively stabilizes the first iteration of the capture loop stage, reducing interrupt timing variability and improving overall synchronization reliability. At line 14, the SoC warm-up stage begins. The for loop runs for three sample captures. Before taking a capture, the sendAndRcvPhaseIdToSoC((i mod 3)) function call at line 16 instructs the SoC through GPIO to change the pattern cyclically for the respective phase IDs (i.e., 0, 1, and 2). It waits until it receives an ACK from the SoC. captureHSImage() at line 18 uses a software trigger to execute a camera capture offered by the Ximea API and store the image in the hs_image object. This feature allows the capture call to be tied directly to the capture loop, ensuring that image acquisition only happens when the algorithm is ready. In other words, this function explicitly tells the camera when to grab and deliver the image. This ensures that acquisition occurs only when downstream processing can handle it immediately, eliminating the need for extra buffering or handshaking. A dummy delay is inserted at line 20 via sleep(STATIC_DELAY) to mimic the timing overhead of the real save(hs_image) call in the capture loop, ensuring that the loop timing behavior produced by the g++ compiled code remains consistent between the warm-up and the capture loops.

#### Appendix B.5.2. Capture Loop Stage

The capture loop, starting at line 23, repeats the same phase-cycling sequence used in the DLP warm-up but saves captured modulated images to the SD card. It runs for S − 1 iterations to acquire S modulated samples, and each captured HS image is saved by the hs_image function call on line 27.

## Appendix C. Results

### Appendix C.1. Perform Experiments

#### Appendix C.1.1. Capture White Reference

As explained in Section 2.3, white reference demodulated HS images were first acquired to preprocess the target sample demodulated HS data. The same white ceramic target used to determine the exposure time in Section 5.1.2 served as a reference. The sample was placed on the projection bed, and the modulated captures were individually recorded in both systems under each experimental configuration using the polarizer setting (213 ms exposure) and no-polarizer setting (39 ms exposure), without applying projection–capture synchronization. Synchronization was omitted for the white reference to ensure that it remained free of possible noise or artifacts introduced by the experimental synchronization process. Then these white reference modulated captures were demodulated using Equation (6).

#### Appendix C.1.2. Supplementary Details on Prepare a Target Sample for Capture

A 3D-printed box with four chambers was specifically designed as the target sample box. Each chamber held four substances: salt, cocoa, coffee, and sugar. The sample box served as the consistent test target in all acquisition configurations to ensure experimental uniformity.

The final modulated captures of the target sample must be compared with a set of reference modulated captures. Two key aspects were considered for the comparison: artifact-free demodulated images and the expected phase shift. However, when the sample box filled the entire FOV of the camera, it was difficult to extract clean modulated spectra for further phase-shift evaluation. Hence, white paper was placed under the sample, leaving a gap at the left edge of the frame. This gap ensured the extraction of a clean sine-wave cycle from the top-left corner of the demosaiced HS image.

Subsequently, a phase plot (PP) was generated from the clean sine-wave cycle by mapping pixel intensities across approximately one sine-wave period. Applying the smoothing Equation (9) produced a smoothed phase plot (SPP), producing cleaner and higher-contrast signals for accurate phase-shift evaluation relative to the reference (Section 5.4.2).

#### Appendix C.1.3. Supplementary Details on Capture Reference and Final Modulated Target Sample

The prepared target sample box (described in Section 5.3.1) was used for both the reference and final modulated captures to ensure consistency. This enabled a direct comparison between the modulation observed in the reference and that of the target sample.

Reference modulated captures were obtained to establish a ground-truth reference. For each exposure time (i.e., polarizer setting, as defined in Section 5.3), each of the three phase-shifted modulated patterns projected on the target sample box with white paper underneath was captured separately without any synchronization. Capturing each phase individually ensured that these images represented ideal references. These reference phase shifts formed the ground truth for subsequent comparisons: both the HSPy-SI and HyperSI systems were evaluated by measuring how closely their synchronized captures matched these benchmarks. Although both systems support this individual capture feature, the HyperSI system was used to perform this task. Finally, all reference modulated captures were demodulated using Equation (6).

Then, the final modulated images of the sample box were acquired for each experimental configuration on both systems. Data acquisition was performed under two settings: with polarizers, using the E-213-S-30 configuration for the SOTA HSPy-SI system and E-213-W-0,1,2,3-S-30 for the HyperSI system, and with no polarizers (i.e., low exposure), using E-39-W-0,1,2,3-S-30 for the HyperSI system. Since the SOTA HSPy-SI system was designed for the polarizer setting (i.e., high exposure time), no captures were taken by the SOTA for E-39 (Section 3.3). In addition, the SOTA HSPy-SI system does not support contiguous real-time-modulated captures (as described in Section 3.4), in contrast to the proposed system. Hence, all ten groups were captured one by one for the SOTA. All runtime performance metrics, such as Actual Capture Time (ACT), Change Pattern Time (CPT), Saving Time (ST), and Total Time (TT), were automatically collected by both systems during the system runtime. They will be described and analyzed in detail in Section 5.5.14 with respect to the real-time measures found after the capture runtime. Finally, all sample modulated captures were demodulated with Equation (6).

### Appendix C.2. Supplementary Details on Prepare Captured HS Image Dataset for Evaluation

#### Appendix C.2.1. Supplementary Details on Experiment for the Initial Selection of the SBI

A miniature spectrometer (Ocean Insight, Orlando, FL, USA) capable of measuring within the VIS and NIR spectra (350–925 nm) and featuring a 16-bit analog-to-digital resolution was used to analyze the spectral responses of the energy coming from the lamp and the projector (using and not using a polarizer). The Ocean View software, v2.0.20 from Ocean Insight was used for the measurements. In Figure 9, the black curves represent the energy emitted by the lamp used as the light source, whereas the red and dashed green curves represent the same light passing through the LLG to the projector, respectively, using and not using a polarizer.

The plot shows that the projector attenuated the energy provided by the lamp from approximately 700 nm onward. When the energy received by the HS camera dropped to approximately 50% or less, artifacts began to appear in the demodulated images at 775 nm and continued up to the final band at 925 nm (corresponding to 951 nm for the HS camera). Illustrating the 16 noisy bands within this range is impractical; therefore, only the last four bands, 924 nm, 932 nm, 940 nm, and 951 nm, are presented to highlight the demodulation artifacts, which were observed regardless of the polarizer configuration or the system used. Figure A11 and Figure A12 illustrate these artifacts for both systems. Therefore, the initial seven bands in the range of 650–775 nm were selected because these bands exhibited better energy responses (i.e., close to or above ≈50%).

#### Appendix C.2.2. Supplementary Details on Preprocess, Demosaic, and Generate Phase Plots of Demodulated HS Images

All demodulated images were preprocessed by calibration (Equation (7)) and spectral correction (Equation (8)), described in Section 2.3, as required by the camera manufacturer. As discussed in Section 3.1, the active resolution of 2045×1085 of the 2D HS image comes with all the spectral bands arranged as a 5×5 mosaic. Hence, the pixel spectra of the original data are fragmented in multiple spatial locations. Demosaicing reconstructs accurate spectral signatures for each pixel location. After applying preprocessing, the 2D HS image was transformed into a cube of size 409×217×24, where 409×217 are the spatial pixels and 24 is the number of spectral bands (665–960 nm). Each band-by-band demosaiced image at a resolution of 409×217 was used for further analysis.

Figure A9 shows how the sample box was prepared for capture and how PPs were generated from both reference and experimental captures for a given spectral band. This method was used to generate SPPs from preprocessed modulated captures for all types of experimental configurations, including both reference and experimental sets. First, a full sine-wave cycle was visually identified as a slice in the modulated image. The first row of pixels spanning the sine-wave cycle slice was then extracted. The PPs were generated from the intensity of the pixels in that slice. To generate an SPP, Equation (9) was applied to the raw pixel values of the slice. The SPP provided clean and high-contrast phase signals that facilitated near-accurate phase-shift analysis when compared to the reference SPP.

However, Figure A10 shows a small, uniform phase shift across the three phases (i.e., 0°, 120°, and 240°) in the SPP generated from captures with a polarizer, relative to the counterpart no-polarizer setting. Consequently, the SPP obtained with the polarizer appears to be “flipped”. This variation occurs because of the small refractive-index mismatch between the two media, that is, the lens of the projector and the polarizer (i.e., the phase shift becomes prominent due to the small gap between the projector and the polarizer). Since the TPD Equation (6) relies only on relative intensity differences, it effectively cancels out this uniform global phase shift. Therefore, this effect is negligible.

After analyzing the SPPs of seven bands in the 650–775 nm range (as selected in Section 5.4.1), four bands, 714 nm, 730 nm, 742 nm and 755 nm, were selected for the final phase-shift evaluation. These wavelengths provide better visual contrast than the others. However, artifacts began to appear in the demodulated images from 775 nm onward and persisted until the final band at 925 nm (corresponding to 951 nm for the HS camera), as the energy received by the camera progressively decreased from about 50% to nearly 5% (Section 5.4.1, Figure 9). Illustrating the 16 noisy bands within this range is impractical; therefore, only the last four bands, 924 nm, 932 nm, 940 nm, and 951 nm, are presented to highlight the demodulation artifacts, which were observed regardless of the polarizer configuration or the SFDI system used. Figure A11 and Figure A12 illustrate these artifacts for both systems.

### Appendix C.3. Supplementary Details on Evaluation of the Demodulation

#### Appendix C.3.1. Supplementary Details on Definition of the Demodulation Criteria

The demodulation grouping scheme from modulated captured images has already been defined and described in Section 5.3. It would be overwhelming to analyze all demodulated images (i.e., S = 30 for each of the experimental configurations). Hence, only the fifth group g-4 is used for the evaluation, as it best represents the steady-state behavior of the system after initial transients have settled during real-time capture. Demodulation is considered acceptable only if both of the following criteria are met:

1. A demodulation is accepted if at least two of the pre-selected bands exhibit phase shifts in the modulated images that closely align with the reference vertical guides of the reference SPP. It is rejected only if the phase shifts show a significant and uniform deviation from the reference across the evaluated bands.
2. The demodulated images should remain free of noise or artifacts.

As the HS snapshot camera captures all 24 spectral bands simultaneously using a shared optical path and sensor, the acquisition conditions are identical in all bands. Therefore, as defined in Criterion 1, if at least two bands demonstrate correct alignment of the SPP marker with the reference, it is reasonable to assume that the remaining bands are also correctly demodulated, given the inherent consistency of the snapshot capture process. Even when Criterion 1 is satisfied, artifacts may still appear. Therefore, both conditions must be met for acceptance. However, defining quantitative thresholds for these phase-shift KPIs is beyond the scope of this study and is left for future work. Figure A13 shows an example SPP comparison for a band, indicating which demodulations are accepted or rejected based on Criterion 1.

For comparison of phase shifts, the SPPs already generated in Section 5.4.2 were used. The phase-intersection points were numerically marked with 1, 2, and 3. Phase flips were observed in Section 5.4.2 (illustrated in Figure A10) under polarizer and no-polarizer captures, causing the phase-intersection positions to differ. With polarizers, markers 1, 2, and 3 mark the phase-intersection points 120° vs. 240°, 0° vs. 240°, and 0° vs. 120°, respectively. Without polarizers, they mark 0° vs. 120°, 120° vs. 240°, and 0° vs. 240°. Three vertical guides have been drawn from markers 1, 2, and 3, and the downward arrows show the measured shifts relative to the reference. If a significant and uniform phase-shift deviation is observed in all the phase-intersection point markers, as shown in Figure A13a, compared to the reference SPP, it indicates that the synchronizer is not working properly, and therefore, the camera recorded the incorrect phase. Since the proposed system used the synchronization tuner W, the SPP explains the behavior of the system with respect to the capture performed. However, small deviations can often occur randomly at the intersection points (i.e., not uniform in all markers) of the phases of the target plots compared to the reference. Two such cases are shown in Figure A13b. Since reference and target captures were acquired in separate sessions, the reflectance of the sample can change over time. As a result, some spectral bands exhibit differences in signal strength between the reference and target SPPs. The smoothing approximation further accentuates these discrepancies, producing non-uniform shifts at the phase-intersection markers. If the demodulation shows no artifacts for such cases, then it can be considered acceptable (i.e., these minor discrepancies are considered negligible).

Figure A14 presents a demosaiced demodulation example used to evaluate the criteria in Criterion 2. In row (i), the region of interest (ROI) of the sample box is highlighted; row (ii) shows a slice from the top-left corner of the demodulated image, where the white reference paper makes even subtle minor artifacts clearly visible across the varied substances (e.g., salt, cocoa, coffee, sugar).

#### Appendix C.3.2. Validation of Frame-Level Synchronization in the HyperSI System

To ensure accurate phase alignment in the proposed HyperSI system, it is first necessary to validate frame-level synchronization. Phase alignment ensures that the pattern phases generated by the SoC and the phases displayed by the DLP projector are synchronized at the frame level. To confirm phase alignment, it is necessary to verify that the interrupt period matches the frame period of the projected frames (i.e., I≈TF). Furthermore, since the key configuration parameter of the proposed HyperSI system is the frame-based interrupt count W for introducing a configurable delay to compensate for projector latency, the accuracy of W directly depends on the interrupt timing behavior. Therefore, it is essential to measure and analyze the actual interrupt arrival period, as well as to validate the design choice (Section 4.8) of the first interrupt being generated from the frame of the previous phase.

The HW was designed with the specific objective of maintaining the equality between the interrupt period and the frame period. After each frame is generated by the SoC, which includes both the active pixels and the blanking periods (i.e., Vblank, Hblank), an interrupt must be triggered from an IP core, which should give an interrupt period equal to the measured frame period (i.e., I≈TF≈17.26 ms). To satisfy this condition, three potential IP cores during HW design were considered for interrupt generation: the pattern-generator IP (i.e., Pattern_kernel), the AXI4S-VOut IP, and the Digilent HDMI IP (i.e., RGB-to-DVI Video Encoder IP). The AXI4S-VOut IP, a general-purpose IP from the Xilinx IP repository, lacks an interrupt output port and is therefore unsuitable for this purpose. Similarly, the Digilent HDMI IP does not provide an interrupt port for complete frame-generation detection; however, even if it did, using it would constrain the design to the Zybo Evaluation board, limiting HW portability. In addition, both the AXI4S-VOut and HDMI IP cores provide a dedicated VSync port that can be engineered as a frame detector. However, converting the VSync signal into an interrupt would add complexity to the HW design. This narrowed the interrupt extraction choice to only one IP, i.e., the Pattern_kernel IP. Therefore, the Pattern_kernel IP was chosen as the interrupt source for this design, as it emits one interrupt upon completion of each frame generation. These interrupts are counted by W to determine the frame-level delay required for synchronization.

Nevertheless, selecting the Pattern_kernel IP as the interrupt source introduces an additional timing-specific consideration within the HW design. Since the Pattern_kernel IP computes only active pixels (i.e., $Pixels_{active}=912\times 1140=1,039,680$), the theoretical interrupt arrival period is supposed to be $I_{theory}=\frac{Pixels_{active}}{PPC\times clk}=\frac{1,039,680}{1\times 86\;\mathrm{MHz}}=12.09$ ms. However, this theoretical estimate assumes that the pattern-generator IP operates without any backpressure from downstream IP cores (i.e., AXI4S-VOut IP, HDMI IP), which is not the case in the actual system. Figure A15 provides a detailed view of the interface between the pattern-generator IP and the AXI4S-VOut IP. The Pattern_kernel IP (i.e., the pattern-generator IP) was designed to comply with the AXI4-Stream protocol and therefore interface with the sink (i.e., AXI4S-VOut IP), along with all sideband handshake signals: TVALID, TREADY, TLAST, and TUSER. This interfacing induces backpressure from the sink (i.e., downstream IP cores started with AXI4S-VOut IP). Through these signals, the AXI4S-VOut IP can slow down or stall the Pattern_kernel when it cannot accept more data. This effect is known as backpressure. As a result, Pattern_kernel does not run freely on its own clock, but rather the pace is controlled by the sink. In practice, this means that the generator produces pixels only when the sink is ready, in particular, when the TREADY signal is asserted.

Hence, the Pattern_kernel IP streams active pixels to the sink (i.e., the AXI4S-VOut IP) only when the s_axis_video_tready signal from the AXI4S-VOut IP is high. Once the blanking periods defined by the VTC IP are parallelly inserted through the vtiming_in port of the AXI4S-VOut IP, s_axis_video_tready becomes low, which is asserted by m_axis_video_out_TREADY of the Pattern_kernel IP. This causes the Pattern_kernel IP to pause/stall until the AXI4S-VOut IP finishes processing the active pixel stream of the current frame, i.e., the insertion of synchronization parameters. Although active pixel generation itself ends (i.e., theoretically ≈12.06 ms), the Pattern_kernel IP remains stalled for the current frame until the VTC finishes adding synchronization signals. Once all blanking intervals are added to the stream of pixels of the current frame, the AXI4S-VOut IP sets the s_axis_video_tready signal high again. Then, the Pattern_kernel IP asserts s_axis_video_tready = 1 through its m_axis_video_out_TREADY port and and resumes back again to end the process of the current frame by emitting one ap_done interrupt through its interrupt port. Thus, the interrupt period *I* corresponds to the full frame period (i.e., TF≈17.26 ms) measured by the oscilloscope from the AXI4S-VOut IP (Section 5.2.2), which includes both active pixel generation and VTC synchronization (blanking) periods added by the AXI4S-VOut IP. Since there are no remaining blanking intervals left to add to the current frame, the AXI4S-VOut IP keeps s_axis_video_tready = 1 so that the Pattern_kernel IP can compute and stream the next active pixels for the next frame. Thus, this cycle continues. Therefore, the actual arrival time of an interrupt I≈TF≈17.26 ms.

To confirm that the HW design performs as intended, a small experiment was conducted to measure the interrupt period and verify that I≈TF≈17.26 ms. The experiment utilized a simplified instrumentation setup consisting of the SBC master controller and the embedded SoC application. For the SoC application, lines 13 to 15 of Algorithm 1 were wrapped inside a software timer to measure the interrupt period. Once booted, the SoC application, by default, begins sending the P0 phase to the HDMI stream. However, a simplified master controller application (pseudocode Algorithm A3 was used to trigger five interrupts (i.e., W = 5, defined on line 3) for the phase transition from P0 to P1 (line 5), carried out in six trials at arbitrary moments. The reason behind performing these trials at arbitrary moments was to capture the actual behavior of the interrupt period and to identify any potential discrepancies in the measured period throughout the interrupt sequences (i.e., 1st to 5th interrupt). All measurements of the interrupt period were taken for the execution of sendAndRcvPhaseIdToSoC(1) on line 5. In particular, here, no warm-up loop was used, as in the original master controller Algorithm A2.

The measurements of the interrupt period in milliseconds obtained from this experiment are presented in Table A3. The column Interrupt Counter indicates the sequence of interrupts emitted by the Pattern_kernel IP. Across experimental trials 1 to 6, the intervals from the 2nd to the 5th interrupt were consistently measured as approximately 17.255 ms, on average (i.e., ≈17.26 ms out of 24 measures) during all phase transitions from P0 to P1. This supports and validates the assumption that I≈TF≈17.26 ms.

**Algorithm A3** SBC Main Function
1: **function** Main 2: // Configure the W = 5 frame count on the SoC 3: sendWaitFrameCountToSoC(5) 4: // By default, the SoC was running P0 phase. This statement tells the SoC to change the phase from P0 to P1 5: sendAndRcvPhaseIdToSoC(1) 6: // Tell the SoC to load back P0 for the next experiment trial 7: sendAndRcvPhaseIdToSoC(0) 8: **end function**

**Table A3:** Interrupt period measurements over six trials in ms for P0-to-P1 phase change.

Interrupt Counter	Trial 1	Trial 2	Trial 3	Trial 4	Trial 5	Trial 6
**1st**	12.366	2.329	11.153	1.741	5.982	10.607
2nd	17.246	17.254	17.255	17.256	17.255	17.256
3rd	17.255	17.256	17.256	17.256	17.256	17.256
4th	17.256	17.256	17.255	17.256	17.256	17.256
5th	17.256	17.256	17.256	17.256	17.256	17.256

However, due to the design choice implemented in the ISR Algorithm 1 (Section 4.8), the first interrupt period $I_{1st}$ deviated from the expected interval (i.e., 17.26 ms). The measurements of $I_{1st}$ were consequently inconsistent, i.e., 12.366 ms, 2.329 ms, 11.153 ms, 1.741 ms, 5.982 ms and 10.607 ms. This design choice was intentionally made to prevent the interruption of the pattern frame stream during phase transitions, as stopping the stream would violate the real-time pattern streaming requirement. Therefore, the non-deterministic timing behavior of the first interrupt (i.e., $I_{1st}$ coming from the frame of the previous phase) during system startup was anticipated.

As discussed in Section 4.8, the SBC can make a GPIO request at any point in time to change the phase ID (i.e., change from P0 to P1) through the ISR Algorithm 1 call, even while the pattern-generator IP is still generating the frame of the previous phase, P0. The statements defined in the ISR (Algorithm 1), from lines 7 to 9, only updated the control register of the Pattern_kernel IP. As a result, these changes took effect only after the HW completed its current task. The call to DISABLE_AUTO_RESTART(pattern_kernel) on line 7 simply disables the auto-restart register. However, at the time this statement was executed, the pattern frame generation of the previous phase was still running. The same applies to the execution of SET_PHASE(pattern_kernel, phases[receivedPhaseId]) to change the phase and ENABLE_AUTO_RESTART(pattern_kernel) to enable the auto-restart control register. After the execution of lines 7 to 9, a while loop enclosed inside a for loop, starting at line 13, was entered to count the interrupts. In the first iteration (i.e., the first interrupt), it detected the remaining duration of the frame generation of the previous phase, which was still in progress during the execution of lines 7 to 9. Since the SBC trial runs to change the phase were conducted at arbitrary moments, and the first interrupt was triggered by the unfinished frame generation of the last active phase, the measurements of $I_{1st}$ were inconsistent. The maximum interrupt period for this design is 17.26 ms. Since an interrupt is simply a voltage pulse, it does not inherently indicate which phase or frame it originated from. Therefore, if the first measured interrupt $I_{1st}$ is found to be less than the frame period (i.e., $I_{1st}<TF$), it can be assumed that $I_{1st}$ originated from the frame of the previous phase. This design choice was deliberately made to ensure that the pattern stream never stops, at the cost of measuring an interrupt from the previous phase.

Similarly, for all experimental capture configurations (i.e., E-39-W-0-S-30 to E-39-W-3-S-30 and E-213-W-0-S-30 to E-213-W-3-S-30), the first measured interrupt, $I_{1st}$, is assumed to come from the frame of the previous phase (i.e., $I_{1st}<TF$ is found for all configurations), then finally it can be proved that the first interrupt originates from the previous phase ID, indicating that the previous frame generation remained active during the static delay (SD). To avoid inconsistency in the first interrupt period, as observed in Table A3, an SoC warm-up stage has been added just before the capture loop in the SBC master controller Algorithm A2. Moreover, this finding will validate the ISR design in Algorithm 1 with respect to the final modulated captures.

For modulated capture experiments, the SBC Algorithm A2 was used. Change Pattern Time (CPT) is the delay (e.g., in milliseconds) before taking a capture. All CPT values for the different experimental configurations were collected during data capture (Figure 13a). From the aforementioned experiment, it was observed that the first interrupt $I_{1st}$ always originates from the frame of the previous phase. Hence, measuring $I_{1st}$ for the final captures is necessary for two reasons. First, it reconfirms the finding from the previous experiment that the first interrupt always originates from the frame of the previous phase. Second, it serves as a prerequisite for accurately characterizing the frame sequence generated by the SoC and managed by the DLP projector, along with their precise timing behavior (Section 5.5.5, Section 5.5.6, Section 5.5.7, Section 5.5.8, Section 5.5.9, Section 5.5.10, Section 5.5.11 and Section 5.5.12). Therefore, a generalized equation to define the CPT (Equation (A14)) is required so that the corresponding general equation to measure $I_{1st}$ can be derived thereafter.

CPT should be measured as the time taken to execute the ISR (Algorithm 1) on the SoC board; i.e., the W count is factored with TF into the CPT, including the SD of 8 ms (Section 4.8). The SD is the period implemented to stabilize the GPIO communication between the SBC and the SoC. Therefore, CPT can be expressed in closed form by Equation (A14).

(A14) $$CPT\approx SD+\left(TF\times W\right)\;\;\;\left[\mathrm{where};W\in Z_{\geq 0}\right]$$

For W = 0, no interrupts are counted. However, for W≥1, the interrupt count begins. Since W counts frame-generation interrupts for a given new phase ID, and each interrupt interval $I_{i}$ approximates the TF (i.e., TF=17.26 ms), $I_{1}\approx I_{2}\approx I_{3}\approx \ldots \approx TF\approx 17.26$ ms. Consequently, TF×W can be written in the form of Equation (A15) as the series sum of all *W* interrupts.

(A15) $$TF\times W\approx \sum_{i=1}^{W}I_{i}\;\;\;\left[\mathrm{where};W\geq 1\right]$$

Therefore, by substituting Equation (A15) into Equation (A14), CPT can be expressed as Equation (A16a). CPT can also be written as the elapsed time of all $I_{Wth}$ interrupts, including SD, in expanded form in Equations (A16b) and (A16c).

(A16a) $$\begin{array}{cc} CPT & \approx SD+\sum_{i=1}^{W}I_{i}\;\;\;\;\;\;\;\;\;\;\;\;\;\;\;\;\;\;\;\;\;\;\;\;\;\;\;\;\;\;\;\;\;\;\;\;\;\;\;\;\;\;\;\;\;\;\;\;\;\;\;\;\;\;\;\;\;\;\;\;\;\;\;\;\;\;\;\;\;\;\;\;\;\;\;\;\;\;\;\;\;\;\;\;\;\;\;\;\;\;\;\;\;\;\;\;\;\;\;\;\;\;\; \end{array}$$

(A16b) $$\begin{array}{cc} CPT & \approx SD+I_{1}+I_{2}+\ldots +I_{W}\;\;\;\;\;\;\;\;\;\;\;\;\;\;\;\;\;\;\;\;\;\;\;\;\;\;\;\;\;\;\;\;\;\;\;\;\;\;\;\;\;\;\;\;\;\;\;\;\;\;\;\;\;\;\;\;\;\;\;\;\;\;\;\;\;\;\;\;\;\;\;\;\;\;\;\;\;\;\;\;\;\; \end{array}$$

(A16c) $$\begin{array}{cc} CPT & \approx SD+I_{1st}+I_{2nd}+\ldots +I_{Wth}\;\;\;\left[\mathrm{where};I_{1}=I_{1st},I_{2}=I_{2nd}\ldots ,I_{W}=I_{Wth}\right] \end{array}$$

Using Equation (A16c), $I_{1st}$ can be measured from the CPT using Equation (A17b).

(A17a) $$\begin{array}{cc} I_{1st} & \approx CPT-SD-(I_{2nd}+I_{3rd}\ldots +I_{Wth})\;\;\;\;\;\;\;\;\;\;\;\;\;\;\;\;\;\;\;\;\;\;\;\;\;\;\;\;\;\;\;\;\;\;\;\;\;\;\;\;\;\;\;\;\;\;\;\;\;\;\;\;\;\;\;\;\;\;\;\;\;\;\;\;\;\;\;\;\;\;\;\;\;\;\;\;\;\;\;\; \end{array}$$

(A17b) $$\begin{array}{cc} I_{1st} & \approx CPT-SD-\left(TF\times \left(W-1\right)\right)\;\left[\begin{array}{c} \mathrm{Sin}\mathrm{ce}\;\mathrm{LHS}\;\mathrm{computes}\;I_{1st},\;\mathrm{RHS}\\ \mathrm{excludes}\;\mathrm{one}\;\mathrm{interrupt}\;\mathrm{count}\;\mathrm{thus},\;W-1 \end{array}\right] \end{array}$$

Two different settings, with a polarizer “Pola” (i.e., E-213) and without a polarizer “NoPola” (i.e., E-39), have been defined; so CPT can be denoted as $CPT_{Pola}$ and $CPT_{NoPola}$. In addition, the measured ap_done interrupt events (in ms) can be defined as $I_{Wth}^{Pola}$ and $I_{Wth}^{NoPola}$. For example, for the “NoPola” setting, the first interrupt is $I_{1st}^{NoPola}$, the second $I_{2nd}^{NoPola}$, the third $I_{3rd}^{NoPola}$, and so on.

Since, for W = 1, the count of interrupts begins after an SD of 8 ms for each frame, this configuration serves as a suitable starting point to measure the first interrupt (i.e., $I_{1st}^{Pola}$ and $I_{1st}^{NoPola}$) under both polarizer and no-polarizer settings. Thus, Equations (A16c) and (A17b) can be written for W = 1 as Equations (A18a) and (A18b) for $CPT_{Pola}$ and (A19a) and (A19b) for $CPT_{NoPola}$, respectively. Both values $I_{1st}^{Pola}$ and $I_{1st}^{NoPola}$, measured respectively as 10.6 and 5.1 ms by Equations (A18c) and (A19c), are less than TF = 17.26 ms. Therefore, these first interrupts come from the frame of the previous phase ID.

(A18a) $$\begin{array}{cc} CPT_{Pola} & \approx SD+I_{1st}^{Pola}\;\left[For\;Case;W=1\right]\;\;\;\;\;\;\;\;\;\;\;\;\;\; \end{array}$$

(A18b) $$\begin{array}{cc} I_{1st}^{Pola} & \approx CPT_{Pola}-SD-\left(TF\times \left(W-1\right)\right)\;\; \end{array}$$

(A18c) $$\begin{array}{cc} I_{1st}^{Pola} & =18.6-8-(17.26\times 0)=10.6\;\mathrm{ms} \end{array}$$

(A19a) $$\begin{array}{cc} CPT_{NoPola} & \approx SD+I_{1st}^{NoPola}\;\left[For\;Case;W=1\right]\;\;\;\;\;\;\;\;\;\;\; \end{array}$$

(A19b) $$\begin{array}{cc} I_{1st}^{NoPola} & \approx CPT_{NoPola}-SD-\left(TF\times \left(W-1\right)\right) \end{array}$$

(A19c) $$\begin{array}{cc} I_{1st}^{NoPola} & =13.1-8-(17.26\times 0)=5.1\;\mathrm{ms}\;\;\;\; \end{array}$$

Similarly, for W = 2 from Equations (A20c) and (A21c), $I_{1st}^{Pola}=4.24$ ms, and $I_{1st}^{NoPola}=5.14$ ms. And for W = 3, Equations (A22c) and (A23c) give $I_{1st}^{Pola}=4.08$ ms and $I_{1st}^{NoPola}=5.18$ ms. In both W cases, the first measured interrupts $I_{1st}$ are less than TF = 17.26 ms. This strongly confirms that these interrupts are triggered from the frame of the previous phase ID. The measured $I_{1st}$ interrupts are also illustrated in Figure 13b for better visual understanding and further use in Section 5.5.5, Section 5.5.6, Section 5.5.7, Section 5.5.8, Section 5.5.9, Section 5.5.10, Section 5.5.11 and Section 5.5.12.

(A20a) $$\begin{array}{cc} CPT_{Pola} & \approx SD+I_{1st}^{Pola}+I_{2nd}^{Pola}\left[For\;Case;W=2\right]\;\; \end{array}$$

(A20b) $$\begin{array}{cc} I_{1st}^{Pola} & \approx CPT_{Pola}-SD-\left(TF\times \left(W-1\right)\right)\;\; \end{array}$$

(A20c) $$\begin{array}{cc} I_{1st}^{Pola} & =29.5-8-(17.26\times 1)=4.24\;\mathrm{ms} \end{array}$$

(A21a) $$\begin{array}{cc} CPT_{NoPola} & \approx SD+I_{1st}^{NoPola}+I_{2nd}^{NoPola}\left[For\;Case;W=2\right] \end{array}$$

(A21b) $$\begin{array}{cc} I_{1st}^{NoPola} & \approx CPT_{NoPola}-SD-\left(TF\times \left(W-1\right)\right)\;\; \end{array}$$

(A21c) $$\begin{array}{cc} I_{1st}^{NoPola} & =30.4-8-(17.26\times 1)=5.14\;\mathrm{ms}\;\; \end{array}$$

(A22a) $$\begin{array}{cc} CPT_{Pola} & \approx SD+I_{1st}^{Pola}+I_{2nd}^{Pola}+I_{3rd}^{Pola}\left[For\;Case;W=3\right] \end{array}$$

(A22b) $$\begin{array}{cc} I_{1st}^{Pola} & \approx CPT_{Pola}-SD-\left(TF\times \left(W-1\right)\right)\;\;\;\;\;\;\;\; \end{array}$$

(A22c) $$\begin{array}{cc} I_{1st}^{Pola} & =46.6-8-(17.26\times 2)=4.08\;\mathrm{ms}\;\;\;\;\; \end{array}$$

(A23a) $$\begin{array}{cc} CPT_{NoPola} & \approx SD+I_{1}^{NoPola}+I_{2nd}^{NoPola}+I_{3rd}^{NoPola}\left[For\;Case;W=3\right] \end{array}$$

(A23b) $$\begin{array}{cc} I_{1st}^{NoPola} & \approx CPT_{NoPola}-SD-\left(TF\times \left(W-1\right)\right)\;\;\;\;\;\;\;\;\;\;\;\;\;\;\;\;\; \end{array}$$

(A23c) $$\begin{array}{cc} I_{1st}^{NoPola} & =47.7-8-(17.26\times 2)=5.18\;\mathrm{ms}\;\;\;\;\;\;\;\;\;\;\;\;\;\;\;\;\; \end{array}$$

In addition, due to the use of the SoC warm-up stage in the SBC Algorithm A2, a consistent $I_{1st}^{NoPola}$ was found within a narrower range of 4.06 to 5.16 ms. Similarly, $I_{1st}^{Pola}$ was derived and computed for $CPT_{Pola}$ found for the polarizer setting and is illustrated in Figure 13b. For $I_{1st}^{Pola}$, a similar consistency in the interrupt period was observed, except for just one outlier occurrence (i.e., 10.6 ms).

Finally, the values computed for $I_{1st}$ in different experimental configurations in the final capture confirm the findings of the previous experiment. For all W = 1, 2, 3 configurations, $I_{1st}<TF$ was observed, demonstrating that the first interrupt consistently originates from the frame of the previous phase ID. The means of the measured CPT values and their corresponding $I_{1st}$ values (using the general Equation (A17b)) were computed. The results are expressed in milliseconds and plotted with 95% confidence intervals (CIs). These plots are presented in Figure 13a,b for all experimental capture configurations.

#### Appendix C.3.3. Supplementary Details on Framework for Phase Synchronization and Projection Analysis

The HyperSI system operates as a tuner-based, interrupt-driven synchronizer that implements configurable frame delays through the configurable W parameter. For an in-depth demodulation evaluation of the modulated captures corresponding to each W configuration, four key processes must be considered together:

- The behavior of the system when the phase-change request is sent from the SBC to the SoC while the SoC is generating frames;
- How the DLP projector receives and manages these frames within its internal dual buffer;
- The reorientation and stabilization behavior of the DMD micromirrors for each frame;
- How the camera perceives and records the projected frames.

To unify these processes under a single analytical perspective, a frame-sequence timeline is introduced to establish a phase-synchronization and projection analysis framework that ensures the demodulation evaluation accounts for every key event occurring during the experimental capture. This timeline provides a unified representation of how all components interact across phases for any W configuration. This section discusses these processes all together using one representative configuration, W = 1. The same analytical framework is then applied to evaluate the demodulation for all other selected W configurations (Section 5.5.5, Section 5.5.6, Section 5.5.7, Section 5.5.8, Section 5.5.9, Section 5.5.10, Section 5.5.11 and Section 5.5.12).

Figure A16a shows exactly how the HS camera observes the frames projected by the DLP projector. It is difficult to combine all the ideas in this one diagram, such as how the SoC board generates frames at high speed (i.e., ≈58 Hz), changes the phases, and halts the ISR until it finishes the count of W interrupts to compensate for the one-frame display lag introduced by the projector, and finally how the camera records the stable frames projected by the DLP projector in its exposure window. In addition, if an interrupt is emitted from a previous phase, it is also difficult to explain it with this diagram.

Hence, Figure A16b is introduced, based on the concept illustrated in Figure 7b, to consolidate and explain the aforementioned ideas using an example phase transition from P0 to P1. A tuner configuration of W = 1 has been chosen for the HyperSI system, because this setting initiates the interrupt count process. This example shows how the camera observes DMD-projected (i.e., DLP projector) frames corresponding to SoC frame generation for each specific phase ID. There are a total of three timelines: SoC frame generation, DLP projector frame input, and DMD micromirror load, respectively, as denoted in rows (i), (ii) and (iii). Row (i) represents the SoC frame-generation timeline. At first, phase P0 generation and stream were in progress before the SBC made a GPIO request to change the phase to P1. Immediately afterward, the SoC board received the phase-change request (i.e., P1) from the SBC. While the request was received, there was still another frame from the previous phase (i.e., P0(F1)) generation in progress, as already proved in Section 5.5.3, partially hidden behind the SD static delay, which can be seen in the SoC frame-generation timeline. Hence, the first interrupt was received from the previous phase, P0. The measurements of different first-interrupt arrival times for different W have been calculated from mean CPT values and are shown in Figure 13b. The second timeline, row (ii), illustrates the one-frame display lag, as described in Section 4.1, Figure 7b. The diagonal dotted arrows connecting row (i) to row (ii) indicate that the buffer cannot have a frame of a particular phase unless the SoC finishes the generation. The DLP projector frame input timeline for row (ii) simplifies the frame management process of the internal dual memory buffer, which introduces a delay of one frame to the actual projection timeline by DMD micromirrors. The diagonal dotted arrows connecting row (ii) to row (iii) indicate that the micromirror cannot initialize the display unless the frame has been loaded into the buffer. The camera actually observes and records the projection timeline (i.e., row (iii)) throughout the exposure window. Finally, the camera only observes in the DMD micromirror load timeline; first it records the incorrect phase P0 and P0(F1) and then partially the initialization of the correct phase P1(F2). Reorientation and stabilization occur throughout the entire period of P1(F2). Each increment in W (i.e., for W≥1) of one interrupt shifts the entire frame sequence uniformly by one position to the left out of the exposure window across all the given timelines.

The frame initialized by the micromirror is marked as a dotted diamond in Figure A16b. Micromirror reorientation and stabilization occupy the entire frame initialization period; i.e., during the end-to-end initialization of each frame, the DMD mirrors transition between their bi-stable ON and OFF tilt states and settle using the entire TF (Section 4.1). Understanding this projector behavior is necessary to know what the camera observes and records during its exposure window. In Section 5.2.2, the FRR was measured at 57.94 Hz, giving a frame period TF=17.26 ms. Consequently, the combined time slots for the green, red and blue channels (Section 4.1) fill the entire frame period (i.e., 17.26 ms), as controlled by the DLPC350 controller. Using Equation (A3), the per-frame time slots are 7.77 ms for green (45%), 6.90 ms for red (40%), and 2.59 ms for blue (15%). The MSB of each of the channels (i.e., 8 bits per channel) contributes the most to the pixel intensity (i.e., brightness) and is therefore exposed for the longest duration, while the LSB contributes the least and is exposed for the shortest time. Thus, the time slots taken for each bit-plane for each color channel were computed using Equations (A7b)–(A7d), and they are listed in Table A4.

**Table A4:** This table presents the 8 bit-plane indices from 0 to 7 (first column), the corresponding binary weights (second column), and the approximate time durations for each bit-plane of the green, red, and blue channels (third, fourth, and fifth columns). The sixth column (GRB Total) shows the row-wise sum across three color channel durations for each bit-plane. The last row shows the total binary weight of 255, and the total time for each channel (green ≈ 7.77, red ≈ 6.90 and blue ≈ 2.59 ms, calculated using Equations (A7b), (A7c), and (A7d), respectively). A GRB total time of 17.26 ms is obtained by summing the eight bit-plane durations across all channels.

8-Bit-Plane Information		Bit-Plane Time			
Bit Index (b)	Weight ($2^{b}$)	Green ($T_{\mathrm{bg}}$)≈	Red ($T_{\mathrm{br}}$)≈	Blue ($T_{\mathrm{bb}}$)≈	GRB Total≈
0	1	0.0305	0.0271	0.0102	0.0677
1	2	0.06095	0.0541	0.0203	0.1355
2	4	0.1219	0.1082	0.0406	0.2709
3	8	0.2438	0.2165	0.0813	0.5418
4	16	0.4876	0.4329	0.1625	1.0835
5	32	0.9752	0.8659	0.3251	2.1672
6	64	1.9505	1.7318	0.6502	4.3345
7	128	3.901	3.4635	1.3003	8.6693
**Total Sum**	**=255**	**7.77** ms	**6.90** ms	**2.59** ms	**17.26** ms

Similarly to the SOTA HSPy-SI demodulation evaluation, a detailed analysis will be presented across all four selected bands (i.e., 714 nm, 730 nm, 742 nm and 755 nm) if any particular configuration satisfies the evaluation criteria in at least one of these bands. Among them, 742 nm has been chosen for demonstration, as it provides better signal strength and visual contrast compared to the others. The quality of the demodulation was assessed using the criteria defined in Section 5.5.1. Each criterion was evaluated in the context of the timing of SoC frame generation and the change of phase, the behavior of the projection with respect to the DMD micromirror load timeline, and the exposure window of the HS camera that sees the frames of different phases. The phase transition case P0 to P1 has been selected to analyze the demodulation with respect to the incrementing W. The image slices were extracted from the top-left corner of the demosaiced modulated and demodulated images for reference and final captures.

The E-39-W-0-S-30 configuration (i.e., no-polarizer setting) was chosen to assess demodulation as the initial evaluation case. Since I≈TF≈17.26 ms, the exposure window of 39 ms is slightly more than double the TF, which clearly reveals that the DLP’s one-frame display lag and the interrupt carried over from the previous phase were seen within the camera exposure window. The rest of the capture configurations, such as E-39-W-1-S-30, E-39-W-2-S-30, E-39-W-3-S-30, E-213-W-0-S-30, E-213-W-1-S-30, E-213-W-2-S-30 and E-213-W-3-S-30, will be discussed for the same phase transition and band. Since the core frame generation and control part of the HyperSI system is managed by the SoC board (i.e., a pure HW/SW system), the frame generation and projection behavior through the DLP projector for the respective W configuration will remain the same; e.g., for W = 1, the behavior will remain the same regardless of the exposure settings (i.e., 213 ms or 39 ms). Furthermore, some projected frames can appear only partially within the exposure period of the camera. Since the exact interleaving sequence for color channels and bit-planes performed by the DLPC350 controller is unknown (Section 4.1 and Section 5.5.9), the analysis adopts a sequence-agnostic view to explain these partially visible frames (e.g., frame P1(F2) in Figure A16b).

#### Appendix C.3.4. Evaluation of E-39-W-2-S-30

Since this configuration met the acceptance criteria, a further analysis was performed for the remaining three chosen spectral bands, 714 nm, 730 nm and 755 nm, together with 742 nm. Figure A17 shows the demodulated capture alongside the phase-shift comparison with the reference.

The demodulated, demosaiced (409×217) images in Row I show clean demodulation without visible noise. In Rows II–III, the phase shifts indicated by markers 1, 2, and 3 (i.e., Row III) closely align with the reference SPP (i.e., Row II) for all bands except at 730 nm, which shows reduced signal strength and slight marker shifts (Section 5.5.2). Overall, the demodulated images remained artifact-free across the four bands. The SPP markers were closely aligned with the reference. Therefore, the results of the demodulation of the HyperSI system for E-39-W-2-S-30 meet the demodulation criteria (Section 5.5.1) and were considered acceptable.

#### Appendix C.3.5. Evaluation of E-39-W-3-S-30

Figure A18 illustrates the captures taken with three frame interrupts for the projection–capture synchronization delay. The first interrupt measured for the previous phase P0(F1) was 5.18 ms (Figure 13b), and then the second and third interrupts were counted for the frames of the correct phases, P1(F2) and P1(F3), respectively. In contrast to W = 2, the correct phase P1(F2) was initialized and stabilized, and it was visible on the micromirror timeline (iii). Consequently, from the very beginning of the exposure window, the camera sees frames of the correct phase. The entire frame sequence in the projection timeline was uniformly shifted to a position to the left of the exposure window. Up to this point, the behavior described for the SoC and the DLP projector for W = 3 remains the same regardless of exposure time settings.

The HS camera sequentially recorded all the correct phases: initialized and stabilized P1(F2), P1(F3) and a partial P1(F4). Consequently, the demodulated image slice was free of visible noise, and the modulated image was identical to the reference. SPP markers 1, 2, and 3 were also aligned with the reference guides and exhibit comparably strong signal strength. These findings validate that the increment in W improves demodulation quality. The E-39-W-3-S-30 configuration meets both demodulation acceptance criteria; it is accepted.

As this configuration satisfied the acceptance criteria, additional analysis was performed for the remaining three chosen spectral bands at 714 nm, 730 nm, and 755 nm, along with 742 nm. Figure A19 presents the demodulated captures together with the phase-shift comparison with the reference.

The demodulated, demosaiced (409×217) images exhibit clean demodulation without any visible noise. In Rows II–III, the phase shifts shown by markers 1, 2, and 3 (Row III) align closely with the reference SPP (Row II) across all bands, except 730 nm (Section 5.5.2). However, the demodulated images were free of artifacts across all four bands, and the SPP markers aligned closely with the reference. Therefore, the results of the demodulation of the HyperSI system for E-39-W-3-S-30 are considered acceptable, as they meet the demodulation criteria (Section 5.5.1).

#### Appendix C.3.6. Evaluation of E-213-W-1-S-30

Since this configuration met the acceptance criteria, a further analysis was performed on the remaining three chosen spectral bands, 714 nm, 730 nm and 755 nm, together with 742 nm. Figure A20 shows the demodulated capture alongside the phase-shift comparison with the reference.

The demodulated, demosaiced (409×217) images show clean demodulation without visible noise. In Rows II–III, the phase shifts marked by markers 1, 2, and 3 (i.e., Row III) align closely with the reference SPP (i.e., Row II), with only tiny misalignments for 714 nm, 730 nm, and 755 nm. In particular, the 730 nm band shows a slightly larger deviation at marker 3 and reduced signal strength (Section 5.5.2) compared to the other bands. Overall, the demodulated images remained artifact-free across the four bands. The SPP markers were well aligned with respect to the reference. Therefore, the results of the demodulation of the HyperSI system for E-213-W-1-S-30 satisfy the demodulation criteria (Section 5.5.1) and are considered acceptable.

#### Appendix C.3.7. Evaluation of E-213-W-2-S-30

Figure A21 illustrates the captures taken with two frame interrupts for the projection–capture synchronization delay. For W = 2, the first interrupt was recorded at 4.24 ms (Figure 13b). The SoC frame generation and DLP projector visualization timeline sequence (i.e., the DMD micromirror load timeline) described in Section 5.5.7 was repeated for this configuration, except for the difference in the exposure window.

For W = 2, the entire frame sequence of the micromirror load timeline uniformly shifted by one frame to the left compared to W = 1. Thus, the HS camera recorded ≈13 sequential image frames, one by one: the incorrect phase P0(F1); the new correct-phase initialization and stabilization P1(F2); P1(F3) to P1(F12); and a partial P1(F13). As two interrupt-count-based delays had been introduced for this configuration, the camera had sufficient time to observe the correct-phase modulations from the sample. Consequently, the demodulated image slice shows no visible noise, and the modulated image matches the reference. A barely visible blur was found in the previous configuration, E-213-W-1-S-30, which had no effect on demodulation. However, just the left shift of an incorrect phase out of the exposure window resulted in a clean and correct modulated image. This finding validates that the polarizer caused sensitivity to the diffuse reflectance acquired by the camera. SPP markers 1, 2, and 3 align with the reference guides and exhibit signal strengths equivalent to those of the reference. Therefore, the findings for the E-213-W-2-S-30 configuration meet both acceptance criteria, and it is accepted.

As this configuration satisfied the acceptance criteria, additional analysis was performed for the remaining three chosen spectral bands at 714 nm, 730 nm, and 755 nm, along with 742 nm. Figure A22 shows the demodulated captures alongside the phase-shift comparison with the reference.

The demodulated, demosaiced (409×217) images show clean demodulation with no visible noise. In Rows II–III, the phase shifts indicated by markers 1, 2, and 3 (Row III) closely align with the reference SPP (Row II), with small misalignments at 714 nm, 730 nm, and 755 nm. For 730 nm, a slight increase in deviation was observed at marker 3, and a weaker signal strength was observed (Section 5.5.2) compared to the other bands. The demodulated images were free of artifacts across all four bands. All SPP markers are closely aligned with the reference. As the results of the demodulation of the HyperSI system for E-213-W-2-S-30 satisfy the demodulation criteria (Section 5.5.1), it is considered acceptable.

#### Appendix C.3.8. Evaluation of E-213-W-3-S-30

Figure A23 illustrates the captures taken with three frame interrupts for the projection–capture synchronization delay. For W = 3, the first interrupt was recorded at 4.08 ms (Figure 13b). The SoC frame generation and DLP projector visualization timeline (i.e., the DMD micromirror load timeline) sequence described in Section 5.5.8 was repeated for this configuration, except for the difference in the exposure window.

For W = 3, the entire sequence of frames of the micromirror load timeline uniformly shifted by one frame to the left compared to W = 2. The HS camera recorded ≈13 sequential image frames: just initialized and stabilized correct phase P1(F2); all correct phases P1(F3) to P1(F13); and a partially visible correct phase P1(F14) in the exposure window. As three interrupt-count-based delays had been introduced for this configuration, the camera had sufficient time to observe the correct-phase modulations from the sample. Consequently, the demodulated image slice shows no visible noise, and the modulated image matches the reference. SPP markers 1, 2, and 3 align with the reference guides and exhibit signal strengths equivalent to those of the reference. Therefore, the findings for the E-213-W-3-S-30 configuration meet both acceptance criteria, and it is accepted.

Since this configuration passes the acceptance criteria, further analysis was performed for the remaining three spectral bands (i.e., 714 nm, 730 nm, and 755 nm, with 742 nm). Figure A24 shows the demodulated captures alongside their phase-shift comparisons with the reference.

The demodulated, demosaiced (i.e., 409×217) images show clean demodulation without visible noise. In Rows II–III, the phase shifts shown by markers 1, 2, and 3 (Row III) align closely with the reference SPP (Row II), except for a tiny misalignment that was observed for the bands at 714 nm, 730 nm, and 755 nm. For the specific behavior of the 730 nm band, see Section 5.5.2. The demodulated images remain artifact-free across all four bands, and the SPP markers aligned with the reference. Therefore, the results of the demodulation of the HyperSI system for E-213-W-3-S-30 are considered acceptable.
